# Cytological, transcriptome and miRNome temporal landscapes decode enhancement of rice grain size

**DOI:** 10.1186/s12915-023-01577-3

**Published:** 2023-04-19

**Authors:** Arunima Mahto, Antima Yadav, Aswathi P. V., Swarup K. Parida, Akhilesh K. Tyagi, Pinky Agarwal

**Affiliations:** 1 National Institute of Plant Genome Research, New Delhi, India; 2 Interdisciplinary Centre for Plant Genomics and Department of Plant Molecular Biology, University of Delhi South Campus, New Delhi, India

**Keywords:** Endosperm, Grain size, Histology, miRNome, *Oryzasativa*, Rice, Seed development, Transcriptome

## Abstract

**Background:**

Rice grain size (GS) is an essential agronomic trait. Though several genes and miRNA modules influencing GS are known and seed development transcriptomes analyzed, a comprehensive compendium connecting all possible players is lacking. This study utilizes two contrasting GS *indica* rice genotypes (small-grained SN and large-grained LGR). Rice seed development involves five stages (S1–S5). Comparative transcriptome and miRNome atlases, substantiated with morphological and cytological studies, from S1–S5 stages and flag leaf have been analyzed to identify GS proponents.

**Results:**

Histology shows prolonged endosperm development and cell enlargement in LGR. Stand-alone and comparative RNAseq analyses manifest S3 (5–10 days after pollination) stage as crucial for GS enhancement, coherently with cell cycle, endoreduplication, and programmed cell death participating genes. Seed storage protein and carbohydrate accumulation, cytologically and by RNAseq, is shown to be delayed in LGR. Fourteen transcription factor families influence GS. Pathway genes for four phytohormones display opposite patterns of higher expression. A total of 186 genes generated from the transcriptome analyses are located within GS trait-related QTLs deciphered by a cross between SN and LGR. Fourteen miRNA families express specifically in SN or LGR seeds. Eight miRNA-target modules display contrasting expressions amongst SN and LGR, while 26 (SN) and 43 (LGR) modules are differentially expressed in all stages.

**Conclusions:**

Integration of all analyses concludes in a “Domino effect” model for GS regulation highlighting chronology and fruition of each event. This study delineates the essence of GS regulation, providing scope for future exploits. The rice grain development database (RGDD) ( www.nipgr.ac.in/RGDD/index.php; https://doi.org/10.5281/zenodo.7762870) has been developed for easy access of data generated in this paper.

**Supplementary Information:**

The online version contains supplementary material available at 10.1186/s12915-023-01577-3.

## Background

Rice is the primary food source for over 3.5 billion people [1], and the present rate of rice grain production will not meet food demands of the population by 2050 [2]. Growth in rice production is expected to be contributed more by yield enhancement than expansion in cultivable land [3]. Since grain size/weight (GS) is the most dependable parameter of grain yield [4], understanding its regulation can help in enhancing crop productivity significantly. Typically, rice grain length varies from 3 to 11 mm, while grain width ranges from 1.2 to 3.8 mm [5]. Increase in GS involves an increase in cell number [6, 7] or cell size [8] or both [9]. This increases sink area for acquiring storage compounds, leading to increased grain weight, thus, establishing a positive correlation between seed size and grain yield [10]. The quest for understanding GS regulation in rice is challenging. As a quantitative trait, it undergoes polygenic regulation and is under environmental influence. Furthermore, various traits of rice grain, namely, length, width, and weight are associated with each other and one gene/quantitative trait locus (QTL) may affect more than one such trait [11].

Grain development in rice generally occurs over a month and can be distinguished by three land mark events viz., cell division, initiation of organs, and maturation [12, 13]. The first land mark event extends from 0 to 2 days after pollination (DAP) where extensive cell division occurs immediately after anthesis followed by triple fusion to form a middle-globular embryo and a syncytial endosperm. Organ initiation on the embryo and cellularization of endosperm begin by 3–4 DAP and are marked as the second landmark process. Maturation involves organ enlargement and maturation of embryo and endosperm, including grain filling, and extends from 5 to 29 DAP. Further, maturation can be divided into distinct sub-stages which include individual organ enlargement on embryo and endoreduplication of endosperm (5–10 DAP), embryo maturation and programmed cell death (PCD) in endosperm (11–20 DAP), and dormant embryo and dehydration of endosperm (21–29 DAP). Storage accumulation occurs during maturation phase. Storage is related to grain filling, which is majorly contributed by carbohydrate and seed storage protein accumulation. Altogether, the process of rice seed development has been defined by five stages as S1 (0–2 DAP), S2 (3–4 DAP), S3 (5–10 DAP), S4 (11–20 DAP), and S5 (21–29 DAP) [12, 14].

GS regulation in rice is extremely diverse with involvement of genes controlling hormones [15], G-protein signaling [16–18], ubiquitin proteasome pathway [19, 20], and starch metabolism [21]. They can also be transcriptional activators [11], microtubule-associated proteins [22], chromatin and histone modifiers [23], or sucrose transporters [24]. Any disruption in the timeline of seed development, such as duration of cellularization [25] and programmed cell death [26], directly affects GS. Spikelet and cell size of husk also regulate GS [8, 15, 27]. GS and weight also depend on endosperm cell size [13], which undergoes four structural changes, namely, coenocyte, cellularization, endoreduplication, and PCD [28]. Coenocyte stage (0–2 DAP) involves rapid cell division, with freely distributed nuclei. During cellularization (3–5 DAP), cell walls are laid centripetally [12, 13]. Transcriptome of rice developmental stages [29] has been analyzed to understand plant and seed development for about a decade [14, 30, 31]. Information available till now describes processes, pathways, and genes in isolation [32]. Comparative transcriptome studies have been employed to study dormancy [33], stress tolerance [34, 35], grain quality, and hybrid vigor [36]. Comparative studies covering the entire span of seed development, from the day of pollination up to maturity, generating a complete picture of GS regulation during seed development, are lacking. This necessitates usage of high-throughput studies focusing on GS regulation in rice.

To elucidate mechanism of GS variation, we have performed comparative transcriptome and miRNome analysis, by RNAseq, of two *indica* rice genotypes, sonasal (SN) and long grain rice (LGR) with contrasting seed sizes, during five seed developmental stages (S1–S5) [12, 37], and flag leaf, thus, covering the entire course of seed development. Morphology and histology of seeds throughout their growth, tracing developmental variations between these two genotypes, has been examined. The expression pattern of important regulatory factors, including transcription factors (TFs) and hormone signaling genes, and genes related to cell cycle, carbohydrate, and seed storage protein (SSP) accumulation has been analyzed, elucidating differences in their temporal regulation between the two genotypes. Molecular singularity of the developmental stage pivotal to GS increment has been defined by stage-wise comparative analyses. Patterns of miRNA and expression of their targets during seed development in both genotypes have been compared. Putative miRNA-target modules essential for controlling GS have been elucidated. Finally, the transcriptome and miRNome data have been integrated and a “Domino effect” model has been postulated summarizing GS regulation. A comprehensive database has been developed and is accessible at www.nipgr.ac.in/RGDD/index.php and 10.5281/zenodo.7762870. Our study amalgamates developmental variations with gene expression and biological pathways, thus, explicating the regulatory networks controlling GS to generate novel comprehensive knowledge, expanding the scope for future endeavors in yield enhancement.

## Results

### Rice genotypes SN and LGR have contrasting seed morphology with distinct developmental timelines

**Fig. 1:**
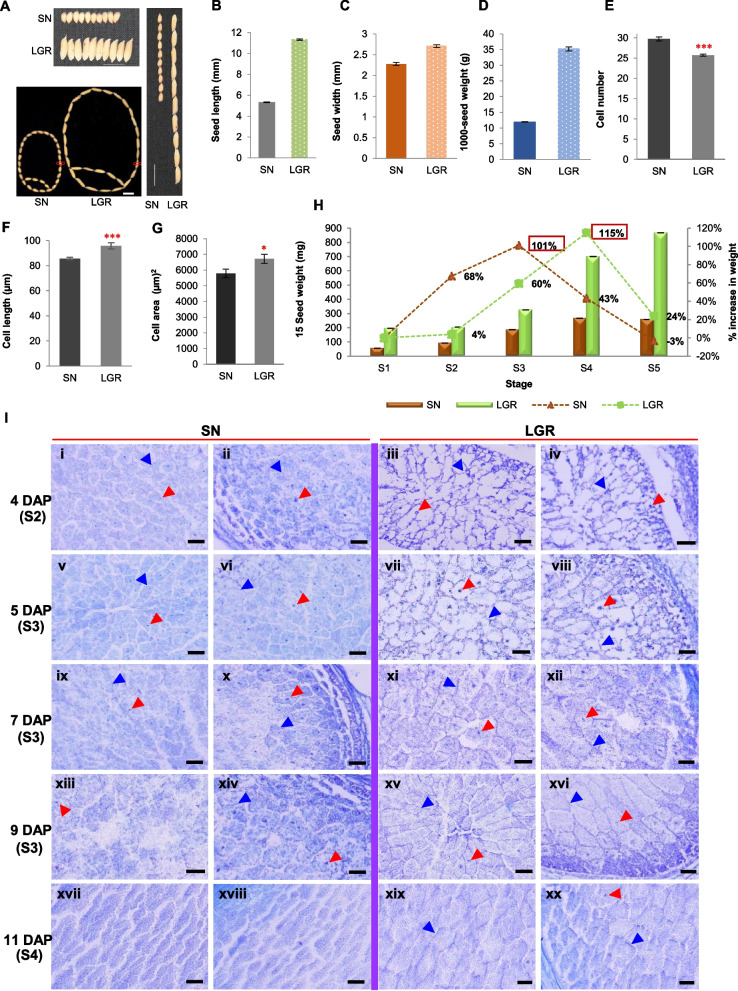
Variation in seed morphology and seed development amongst SN and LGR. **A** Mature seeds of SN and LGR arranged width-wise (top panel) and length wise (right panel, scale bar = 2 cm) and an equal number of mature dry husked seeds of SN and LGR, (scale bar = 1 cm) arranged to resemble a rice seed, show LGR seeds are bigger in size and cover more area. Red box represents middle seed portion used for SEM analysis. Graphs representing **B** length (mm) and **C** width (mm) of a mature seed of SN and LGR (*n* = 100). **D** Average 1000-seed weight (grams) of mature dried seeds (*n* = 3) (See replicate data in Additional file 2: Table S18). Graphs showing **E** cell number, **F** cell length (μm), and **G** cell area (μm^2^) from middle portion of SN and LGR husks (*n* = 6*3). Asterisks are as determined by Student’s *t* tests, one-sided (*, **, *** = *p* value ≤ 0.05, ≤ 0.01, ≤ 0.001, respectively). **H** Grain filling rate of SN and LGR. Bars represent total weight of 15 seeds from each stage (*n* = 3) (See replicate data in Additional file 2: Table S18). Dotted lines indicate per cent increase/decrease in seed weight in one stage with respect to previous stage. The triangles and squares represent SN and LGR, respectively. Error bars represent ± SD in all the graphs. **I** Endosperm sections of SN (left) and LGR (right) at selected DAP, representing S2, S3, and S4 stages of seed development stained with toluidine blue-O. *i, v, ix, xiii, xvii* and *iii, vii, xi, xv, xix* show central endosperm of SN and LGR, respectively, while *ii, vi, x, xiv, xviii *and *iv, viii, xii, xvi, xx* show peripheral endosperms of SN and LGR, respectively. *i-iv, v-viii, ix-xii, xiii-xvi* and *xvii-xx* represent 4, 5, 7, 9, and 11 DAP (as labelled). Red and blue triangles indicate nuclei and cell wall, respectively. Scale bar = 50 µm

For comparison of GS, two *indica* rice genotypes, SN and LGR, showing marked morphological variations (Fig. 1A), were selected. Mature seeds of LGR were twice as long and 1.2 times as wide and weighed 3.5 times more than SN seeds (Fig. 1B,D). Scanning electron microscopy (SEM) analysis of lemma (Fig. 1E, Additional file 1: Figure S1A) revealed that LGR spikelet husk cells were larger than SN (Fig. 1F, G). Full seed dimensions were obtained by 7 DAP (mid S3) in SN, and 13 DAP (early S4) in LGR (Additional file 1: Figure S1B). Accordingly, maximum increment in seed weight occurred from S2-S3 and S3-S4 stages, in SN and LGR, respectively (Fig. 1H). In SN, cell walls and nuclei were distinctly visible in both central and peripheral regions of endosperm at 4 DAP (S2), suggesting complete cellularization (Fig. 1I and Additional file 1: Figure S1C; i, ii, v, vi). At 4–5 DAP in LGR, complete cell walls were more clearly visible towards periphery, indicating ongoing cellularization (Fig. 1I and Additional file 1: Figure S1C; iii, iv, vii, viii), which was completed by 7 DAP, suggesting a longer cell division phase (Fig. 1I and Additional file 1: Figure S1C; xi, xii). At 7 DAP, SN endosperm showed a loss of cellular organization, indicated by the absence of cell walls from the central portion, suggesting an onset of PCD (Fig. 1I; ix, x). Contrastingly, LGR had an increased cell size at 9 DAP unlike SN, highlighting the importance of S3 stage for cell size increment (Fig. 1I and Additional file 1: Figure S1C; xiii, xiv, xv, xvi). In addition, PCD was prominent at 11 DAP in LGR (Fig. 1I and Additional file 1: Figure S1C; xvii, xviii, xix, xx). Hence, cytologically, cell expansion as indicated by larger cell size of endosperm and husk; extension of early cell division to S2 stage; delayed cellularization and PCD appeared important for an increased GS in LGR.

### Comparative transcriptome analyses imply variation in advancement of seed development between SN and LGR

A total of 330.4 Gb of clean data was generated by the sequencing of 36 cDNA libraries, covering three biological replicates of the five stages of seed development and flag leaf (as vegetative control) from each genotype (Additional file 2: Table S1). qRT-PCR of five genes in both SN and LGR validated the RNA sequencing data. Principal component analysis (PCA) analysis confirmed the average Pearson’s correlation coefficients of 0.94 and 0.90 obtained between the biological replicates of SN and LGR, respectively (Additional file 1: Figure S2A, B, C; Additional file 2: Table S2). A total of 5565 and 5453 genes were expressed specifically (FPKM ≥ 1 in any seed stage and FPKM < 1 in flag leaf of that genotype) in the five seed developmental stages of SN and LGR, respectively (Additional file 2: Table S3). Of these, 3563 genes were expressed during seed development in both SN and LGR. Enrichment of functional categories related to growth and development, particularly reproductive development, was seen in them (Additional file 1: Figure S3).

A comparative transcriptome study of the five stages of seed development in SN and LGR elucidated the molecular mechanisms which caused delayed progression of seed development in LGR. Differentially expressed genes (DEGs) were identified with respect to flag leaf (as a vegetative control) in both genotypes (Additional file 2: Table S4) to identify changes in transcriptome during seed development. Flag leaf of either genotype served as a pivot to remove genotypic differences between SN and LGR, against which all DEGs were calculated. These DEGs, responsible for seed development in SN and LGR, were subsequently compared between the two genotypes to extract any changes relevant to GS. SN seeds had more DEGs than LGR, suggesting that its transcriptome underwent more changes than LGR during seed development (Additional file 1: Figure S4A). To highlight pathways crucial for seed development and seed size regulation, differentially expressed genes with log_2_fc ≥ 10 in the two genotypes (1413 upregulated and 543 downregulated; and 1150 upregulated and 95 downregulated genes of SN and LGR, respectively) were functionally annotated (Additional file 1: Figure S4B). Pathways including cell wall, development, and RNA regulation of transcription had ≥ 50 upregulated genes. In the cell wall category, majority of the genes were associated with cell wall modification, degradation, and cellulose synthesis in both SN and LGR. Genes for cell wall associated arabinogalactan proteins (AGPs) were more in number in LGR (7 genes) than SN (2 genes). Under the category “development,” SSPs and late embryogenesis abundant proteins (LEA) encoding genes numbers were high in both SN and LGR. In RNA regulation of transcription category, ten TF families had at least five DEGs (Additional file 1: Figure S4C). Their abundance has been observed in previous seed transcriptome studies suggesting their importance in seed development [12, 14, 37–40]. Additionally, more Gly-Asp-Ser-Leu (GDSL) motif lipases were upregulated in LGR (13 genes) than SN (9 genes).

**Fig. 2:**
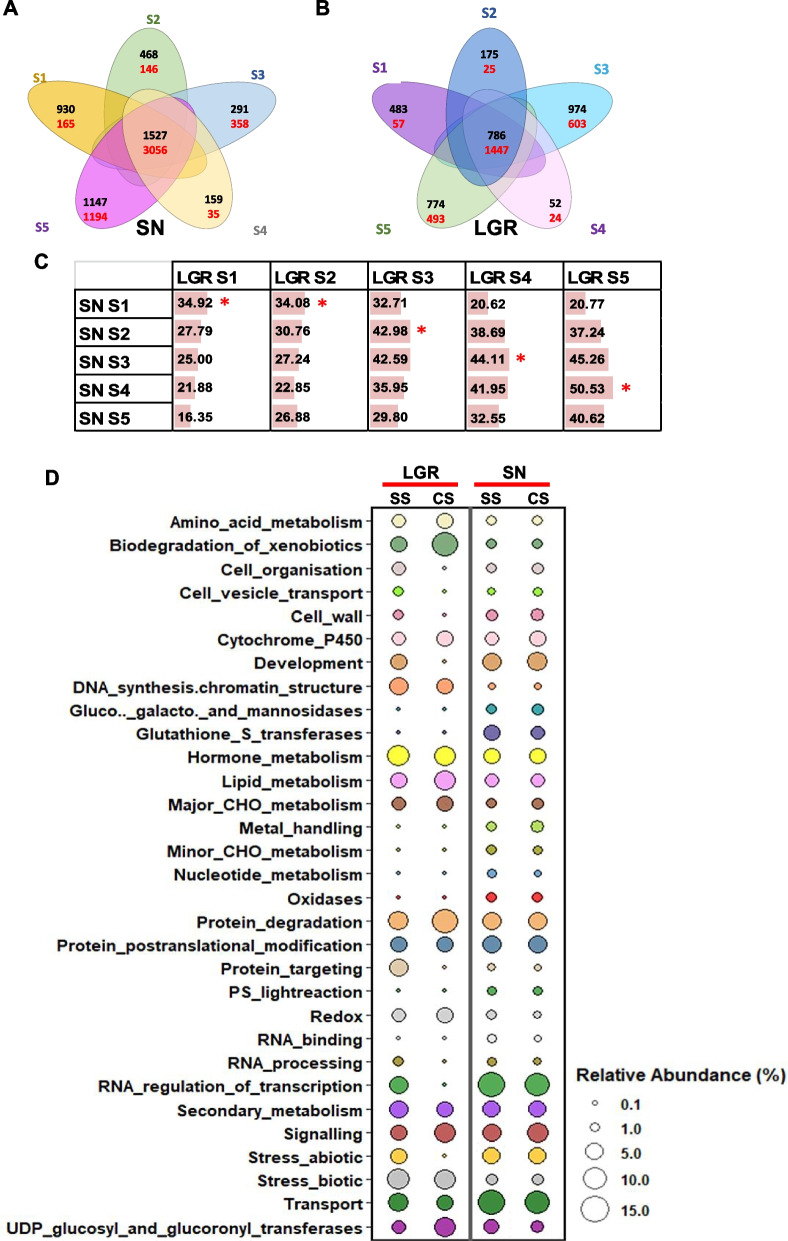
Comparative analysis of differential expression profiles of SN and LGR. **A, B** Total number of differentially expressed genes specific to a seed developmental stage and genes common to all five stages were identified for SN and LGR, respectively (up- and downregulated genes have been written in black and red color in the Venn diagrams, respectively). **C** Figure representing percent similarity observed between S1 and S5 stages of LGR with SN. Maximum percent similarity observed for each stage of LGR with SN has been marked with asterisks beside the bars. **D** Pathway annotation of DEGs with opposite regulation (upregulated in SN and downregulated in LGR and vice versa) in seeds of SN and LGR. DEGs showing opposite regulation in same seed developmental stages (SS; i.e., SN S1-LGR S1, SN S2-LGR S2) and comparable seed developmental stages (CS; i.e., SN S1-LGR S2, SN S2-LGR S3, SN S3-LGR S4, SN S4-LGR S5) of SN and LGR were identified and assigned pathways. Relative percentage of total number of DEGs falling in various pathways for the abovementioned SS and CS comparisons were plotted. Left and right panels represent pathways obtained for SS and CS comparisons for DEGs upregulated in LGR and downregulated in SN, and DEGs upregulated in SN and downregulated in LGR, respectively. Circle radius is proportional to relative percentage of DEGs present in the pathway, as indicated in the figure legend

On examination of stage-specific DEGs (with log_2_fc ≥ 1 in one stage and log_2_fc < 1 in other stages) and DEGs common to all stages (with log_2_fc ≥ 1 in all five stages) of SN and LGR, SN S5 and LGR S3 were found to have the maximum stage-specific DEGs (Fig. 2A, B). There was only slight percentage similarity amongst the stage-specific DEGs of SN and LGR (Additional file 1: Figure S5, Additional file 2: Table S5), indicating considerable transcriptome variations between the same seed development stage of SN and LGR. Each stage of LGR was most similar to the previous stage of SN as indicated by the comparison of DEGs between all stages amongst SN and LGR. This suggested a slower pace of transcriptome changes in LGR (Fig. 2C). For simplicity, SN S1—LGR S2, SN S2—LGR S3, SN S3—LGR S4, and SN S4—LGR S5 pairs will be now called as comparable stages (CS). Comparisons for stages same by name will be called SS (same stage). Most of the oppositely regulated DEGs between SN and LGR belonged to the same pathways as shown by their pathway annotation (for both SS and CS pairs). This implied that similar functions are modulated by different genes in the two genotypes. A greater number of genes were upregulated in the DNA synthesis category, in LGR, in both SS and CS (Fig. 2D). Further, the importance of cell related processes, development, and RNA processing was reiterated in GS control by more numbers of upregulated genes in LGR, in SS than CS.

### GS is increased by transcriptome reprogramming in LGR S3 stage and cell cycle extension

**Fig. 3:**
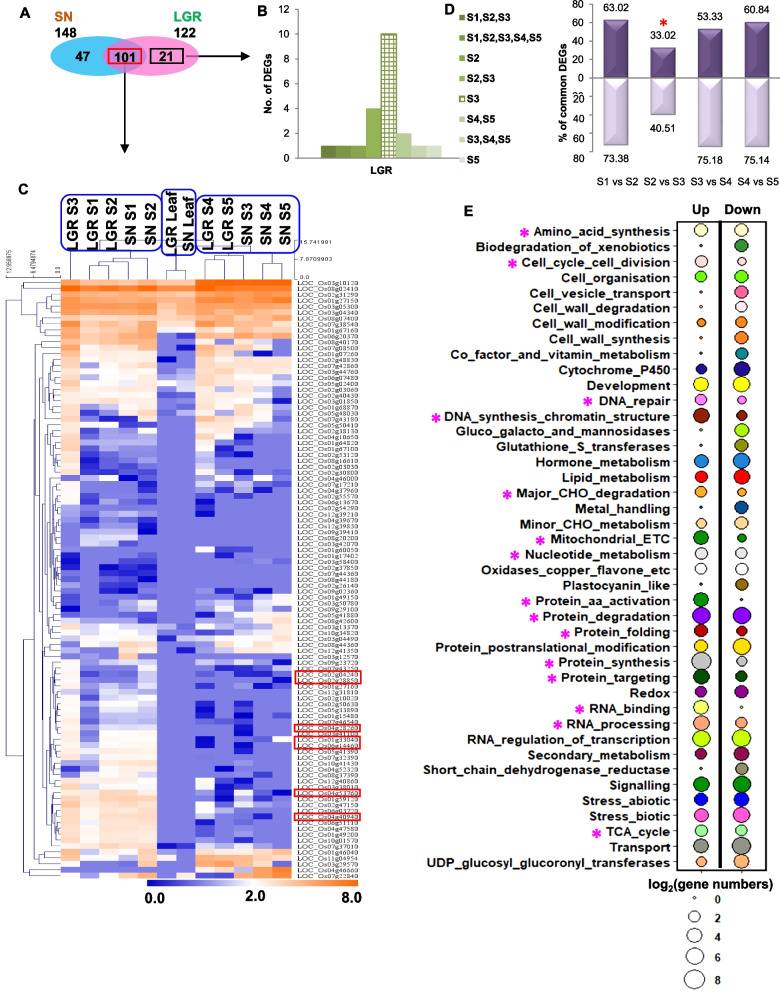
Expression pattern of cell cycle genes in SN and LGR and genes preferential to LGR S3 stage. **A** Venn diagram showing the number of upregulated cell cycle transcripts common and specific to the seed tissues of SN and LGR. **B** The distribution of the LGR-specific genes (denoted by black box in a) was plotted (as represented in the color legend). S3 stage of LGR had the maximum number of cell cycle-related upregulated genes (marked by asterisk in the graph). **C** Heat map by hierarchical clustering was prepared to study the expression pattern (log_2_FPKM) of the cell cycle genes commonly upregulated in the seed tissues in SN and LGR (denoted by red box in a; S1–S5 represent seed developmental stages, Leaf represents flag leaf). LGR S1–S3 grouped with SN S1–S2, as marked with blue boxes. Genes that promote endoreduplication and have different expression patterns in SN and LGR have been marked with red boxes. **D** Percent similarity between DEGs of consecutive stages of LGR (bars above and below the axis represent up- and downregulated genes, respectively). Least similarity was observed between the transcriptomes of LGR S2 and S3 stages as marked by asterisk in the graph. **E** Pathway analysis of DEGs (using MapMan) that are preferential to LGR S3, i.e., upregulated in LGR S3 but not in LGR S2 and SN S3. Pathways related to cell enlargement and enhanced cellular activity, such as cell cycle, DNA synthesis and repair, degradation of starch, mitochondrial ETC, protein synthesis, nucleotide metabolism, and TCA cycle, were distinctly upregulated (≥ 50% genes upregulated out of total DE genes; demarcated with pink asterisks in the figure). Size of circle represents log_2_ gene number present in a category as indicated in the legend

The gene ontology (GO) categories of all upregulated genes in seeds of both the genotypes showed LGR-specific enrichment of cell cycle and DNA metabolic process, and higher enrichment of macromolecule biosynthetic process, gene expression, translation, and carbohydrate metabolic process, in LGR (Additional file 1: Figure S6A). These functions might have contributed to GS increment in LGR by enhancement of cell division and/or DNA content. In addition, the cell cycle-related genes which were specifically upregulated in the two genotypes were majorly contributed by LGR S3 and SN S5 stages (Fig. 3A, B, Additional file 1: Figure S6B, and Additional file 2: Table S6). Further, three clusters were formed by hierarchical clustering of 101 cell cycle-related DEGs which were commonly upregulated in the seed tissues of SN and LGR (Fig. 3C). This clustering implied similarity in expression pattern of cell cycle-related genes between SN S1-S2 and LGR S1-S3. A prolonged cell cycle was indicated by the prominence of cell cycle-related genes in LGR S3 stage. Alongside, promoters of endoreduplication [41, 42] showed peak expression in SN S2 and LGR S3, few even with higher values in LGR (Fig. 3C, and Additional file 1: Figure S6C).

In LGR, along with the maximum number of DEGs being present in S3 stage (Fig. 2B), there was a considerable change in the transcriptome between S2 and S3 stages, unlike SN (Fig. 3D, Additional file 1: Figure S7A). Furthermore, LGR S3-preferential DEGs (genes that were up- or downregulated in LGR S3 stage but not in SN S2/S3 and LGR S2) were identified to study transcriptome changes unique to LGR S3 (Additional file 2: Table S7). Pathways prominent amongst these DEGs were tricarboxylic acid (TCA) cycle and mitochondrial electron transport chain (ETC), which suggested enhanced energy production (Fig. 3E, and Additional file 1: Figure S7B); sucrose degradation, which provided precursors for TCA cycle [43]; cell wall modification, which was in consonance with cytological sections (Fig. 1I and Additional file 1: Figure S1C); amino acid biosynthesis, enhanced protein synthesis; DNA synthesis and repair as well as nucleotide metabolism which regulates plant growth and development. It is known that an ATP surge occurs during cell enlargement phase of seed development [44]. Hence, a transcriptome reprogramming stimulating DNA and protein synthesis, energy production, and cell expansion was evident in the LGR S3 stage.

### Reserve accumulation and PCD are delayed in LGR

**Fig. 4:**
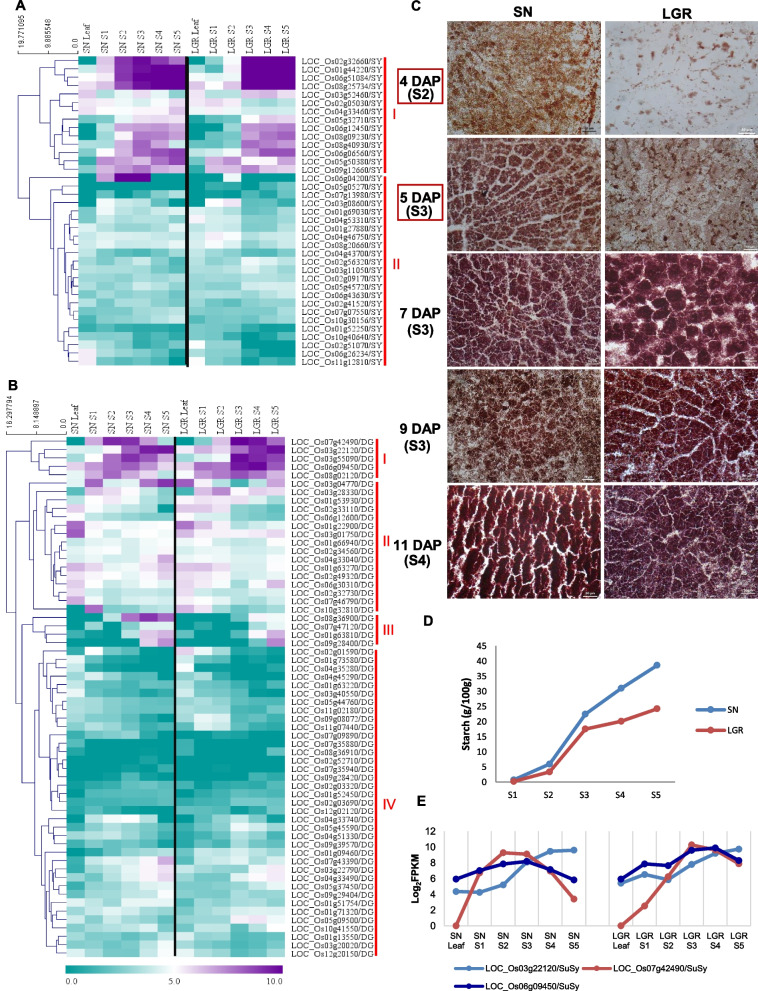
Carbohydrate metabolism in seed stages (S1–S5) and flag leaf (Leaf) of SN and LGR. **A, B** Heat map showing expression levels (log_2_FPKM) of genes related to carbohydrate biosynthesis (SY, two clusters) and degradation (DG, four clusters), respectively. **C** KI/I_2_ staining of the endosperm sections of SN and LGR. The representative DAP from three stages of seed development have been mentioned on the left side and corresponding stages have been mentioned below the DAP. Left and right panels show sections of SN and LGR seeds, respectively (as indicated on the top). Scale bar = 50 µm. **D** Starch estimation in different seed developmental stages of SN & LGR (*n* = 2). The five stages of seed development are represented on the *X*-axis. The *Y*-axis shows the amount of starch in grams per 100 g of seed. Blue line represents SN while red line represents LGR. Standard error bars have been shown (See replicate data in Additional file 2: Table S18). **E** Three sucrose synthase genes showed high expression (log_2_FPKM ≥ 5) in seed tissues of SN (upper panel) and LGR (lower panel). LOC_Os06g09450 and LOC_Os07g42490 showed peak expression in LGR S3, S4 and SN S2, S3, respectively, as marked with arrows

Carbohydrate and SSPs form the bulk of storage reserves in rice endosperm. Intensified expression levels (log_2_FPKM ≥ 5) were seen for both carbohydrate biosynthetic and degradation genes, from SN S2 and LGR S3 stages onwards (Fig. 4A, B, clusters I). This indicated delayed carbohydrate biosynthesis in LGR, which was further validated by I_2_-KI staining of the endosperm sections (Fig. 4C), starch content during five stages of seed development in SN and LGR (Fig. 4D), and expression patterns of three sucrose synthase genes (Fig. 4E). SN seeds had overall higher starch content. Though a surge in starch content was visible in S3 stages for both SN and LGR, the S2 stage of SN had more starch than LGR S2 stage, as was also evident from histological sections. Differences in energy supply and starch accumulation at different intervals between SN and LGR were suggested by patterns of carbohydrate degradation genes (Fig. 4B, Additional file 1: Figure S8A). Cluster II (Fig. 4B) genes had low expression (log_2_FPKM < 5) in SN seeds but high expression (log_2_FPKM ≥ 5) in LGR during initial stages (S1-S2), indicating the presence of sugars for energy production, and a higher metabolic activity.

**Fig. 5:**
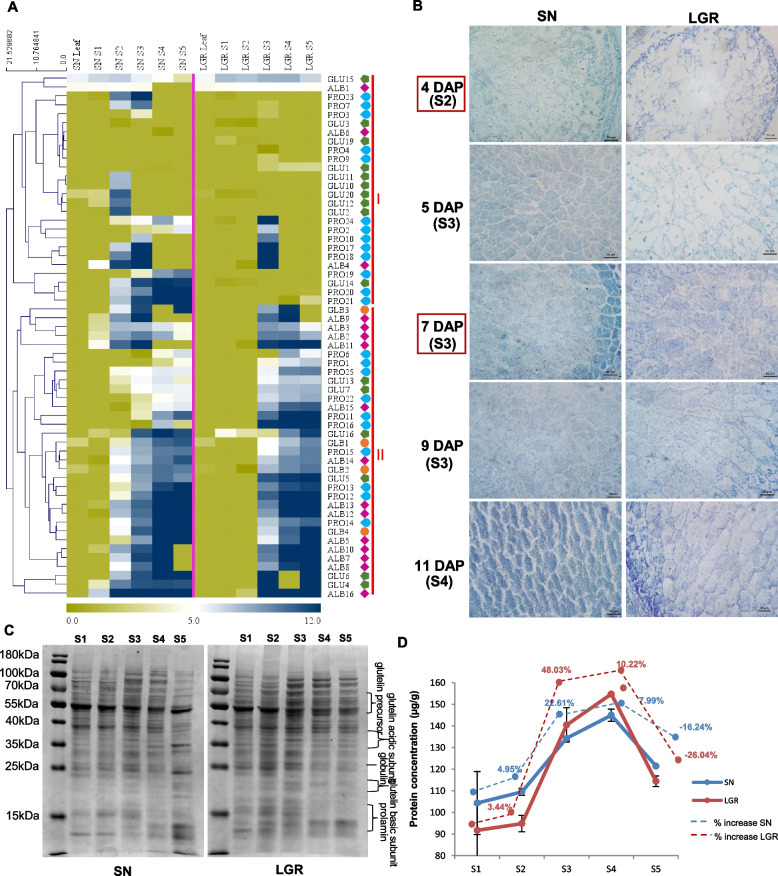
SSP synthesis in SN and LGR. **A** Heat map based on log_2_FPKM values of 65 rice SSP encoding genes. Pink diamonds, blue drops, green pentagons, and orange dots represent albumins (ALB), prolamins (PRO), glutelins (GLU), and globulins (GLB), respectively. **B** CBB staining of the endosperm sections of SN and LGR. The representative DAP from three stages of seed development have been mentioned on the left side and corresponding stages have been mentioned below the DAP. Left and right panels show sections of SN and LGR seeds, respectively (as indicated on the top). Scale bar = 50 µm. **C** Total seed protein isolated from five seed developmental stages in SN and LGR and run on 10% denaturing PAGE, with prominent bands marked. **D** Total protein concentration in each stage of SN (blue line) and LGR (red line) as estimated by Bradford’s assay. The dotted lines (blue for SN, red for LGR) indicate percentage increase/decrease in protein concentration in that stage with respect to the previous one (See replicate data in Additional file 2: Table S18)

SSP encoding genes were prominently expressed from SN S2 and LGR S3 stages onwards (Fig. 5A). In the endosperm sections, total protein staining was visible at 4 DAP in SN, which increased at 5 DAP (Fig. 5B). However, protein staining was significant in LGR only at 7 DAP. The same was reflected in the total protein isolated from SN and LGR S1–S5. Prominent polypeptide bands appeared in SN S2 and LGR S3, which intensified in the later stages (Fig. 5C). The total protein yield estimated from each stage of SN and LGR seed development showed more protein in SN S1 and S2 stages in comparison with same stages from LGR (Fig. 5D). However, the highest protein yield was seen for LGR S3 and S4 stages. Also, the percentage increment in the total protein concentration of a stage, with respect to the previous stage, amongst all comparisons, was highest for LGR S3. Collectively, storage accumulation started from SN S2 and LGR S3, which correlated with the grain filling rate (Fig. 1H), and indicated delayed starch and SSP biosynthesis in LGR. As regards to PCD (Fig. 1I, and Additional file 1: Figure S8B, C), four positive regulators, *OsVPE1, OsVPE3* [45]*, SDS2* [46], and *OsZHOUP1* [47], and a negative regulator, *OsSRT1* [48], exhibited differences in timing and levels of expression between SN and LGR. Hence, PCD was much more rapid in SN and may be an important contributor to GS.

### TFs and hormones regulate GS variation in SN and LGR

**Fig. 6:**
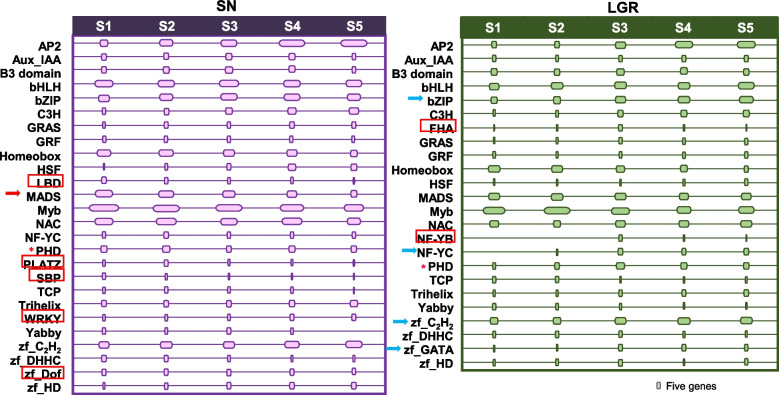
TF families with significant number of member genes upregulated in seeds of SN and LGR. TFs with log_2_fc ≥ 2 in any of the five seed developmental stages of SN and LGR were grouped into families. Those with ≥ 5 members in at least one stage were considered significant and their numbers represented diagrammatically for SN (left panel) and LGR (right panel). Families with at least three members in early (S1 and S2) than later stages (S3-S5), and vice versa, and showing genotype-preference (pattern seen only in SN or LGR) have been marked with red and blue arrows, respectively. For instance, MADS has been marked with red arrow as it has 25 and 22 genes in SN S1 and S2, respectively, while there are 18, 14, and 9 genes in SN S3, S4, and S5, respectively. However, in LGR, it has 16 and 17 genes in S1 and S2, respectively, while 16, 13, and 14 genes in S3, S4, and S5, respectively. TF families with ≥ 5 members in one genotype only have been marked in red boxes. PHD TF family with opposite pattern, i.e., minimum number of members in SN S3 stage and maximum in LGR S3 stage, has been marked with asterisks. The sizes of the boxes are proportional to the number of TF encoding genes in a seed developmental stage as denoted by the scale at the bottom

TFs and hormone signaling genes regulate GS in rice by affecting cell division [49], cell elongation [15], and/or seed filling [12, 50–53]. In our data, 813 and 130 DEGs coding for TFs showed similar or opposite regulation, respectively, between SN and LGR seeds (Additional file 1: Figure S9). The ones with log_2_fc ≥ 2 (Additional file 2: Table S8) were analyzed stage-wise (Fig. 6). Families with at least three more upregulated members in either early (S1–S2) or late (S3–S5) seed development stage of a genotype were called genotype-stage preferential and were considered important for GS regulation. bZIP, NF-YC, C_2_H_2_, and GATA families were LGR-late stage preferential, while MADS TF family was SN-early stage preferential. In addition, FHA and NF-YB families had ≥ 5 upregulated genes only in LGR S3 stage, marking their relevance in GS regulation. On the other hand, LBD, PLATZ, SBP, WRKY, and DOF families had ≥ 5 upregulated genes only in SN. Plant homeodomain (PHD) finger TF family members exhibited opposite trends with peak numbers in LGR S3 and least in SN S3.

**Fig. 7:**
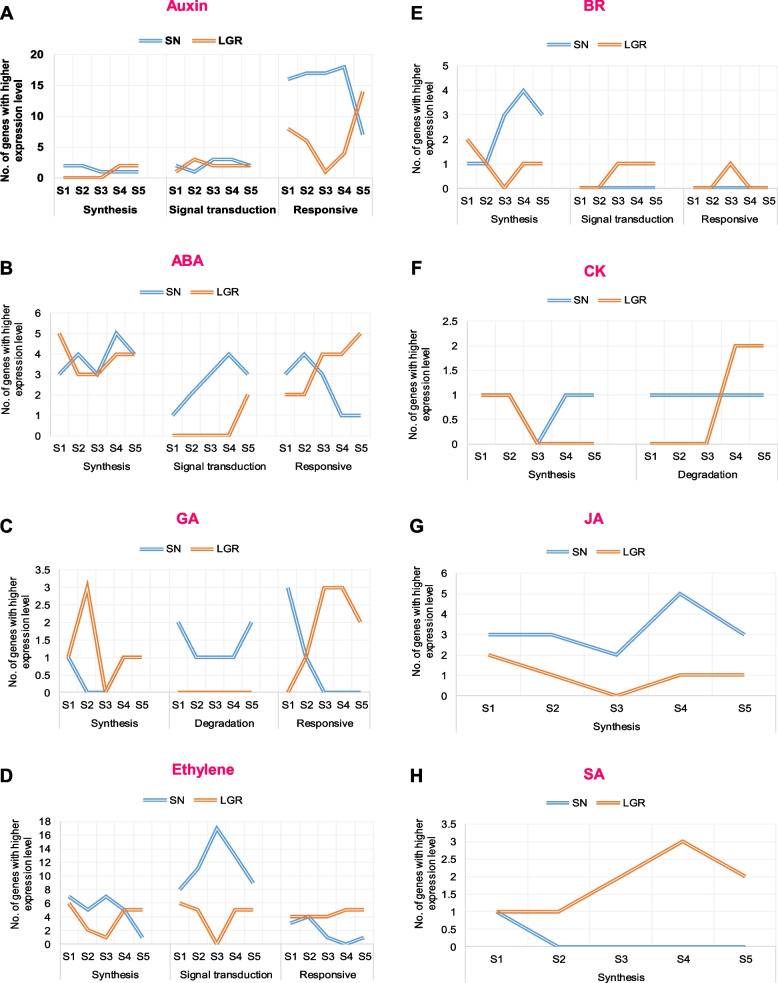
Variation in expression levels and pattern of hormone-related genes in SN and LGR. Genes related to hormone signaling that showed different expression pattern between SN and LGR and hence were present in separate clusters in Additional file 2- Figure S10, were extracted. Their expression levels (log_2_FPKM) in SN and LGR were compared in a stage-wise manner. Number of genes, which had higher expression values (difference in log_2_FPKM ≥ 0.5) in a stage in either SN or LGR, was counted. **A**–**H** Graphs represent the number of genes in different functional categories (biosynthesis, signal transduction, degradation, and response as mentioned below each graph) with higher expression in either SN or LGR for auxin, ABA, GA, ethylene, BR, CK, JA, and SA, respectively, in the five stages of seed development. This means in **A** there are 16 auxin responsive genes whose log_2_FPKM value is at least 0.5 more in SN S1 stage than in LGR S1 stage, while reverse is true for 8 genes. Blue and orange lines indicate SN and LGR, respectively, as shown in the figure legends in each graph

Genes related to eight phytohormones were grouped into co-expression clusters. The cluster numbers for auxin, abscisic acid (ABA), gibberellic acid (GA), cytokinin (CK), brassinosteroid (BR), ethylene, jasmonic acid (JA), and salicylic acid (SA) biosynthesis and signaling-related genes were 13, 10, 10, 9, 10, 15, 8, and 9, respectively. Same genes categorizing into different clusters between SN and LGR might be responsible for variation in GS, on account of differences in their expression pattern (Additional file 1: Figure S10). Out of these, genes showing higher expression (log_2_FPKM ≥ 0.5 than the other genotype) in the same stage when compared between the two genotypes should be significant for GS. The number of such genes were counted and plotted (Fig. 7, Additional file 2: Table S9) giving an indication of the difference in expression patterns and levels of phytohormone-related genes which could participate in GS regulation. Here, genes related to auxin, ABA, GA, and ethylene exhibited opposite patterns of higher expression between SN and LGR during seed development (Fig. 7A–D).

Under auxin-related genes, two biosynthesis genes showed higher expression in SN S1–S2 and LGR S4–S5; three signal transduction genes showed higher expression in SN S3–S4 and LGR S2; and 16–18 auxin responsive genes showed higher expression in SN S1-S4; and 14 showed higher expression in LGR S5 (Fig. 7A, Additional file 1: Figure S10A). This difference between SN and LGR indicated an opposite pattern of higher expression of auxin-related genes amongst the two genotypes—early stages (S1-S2) in SN and later stages (S3-S5) in LGR. Four to five ABA biosynthetic genes had higher expression in SN S2, S4, and LGR S1 stages. Genes encoding ABA signaling components had higher expression in all SN stages. More ABA responsive genes had higher expression in SN S1–S2 and LGR S4–S5 stages (Fig. 7B, and Additional file 1: Figure S10B). This suggested higher ABA signaling in SN early seed stages as opposed to later stages in LGR (Fig. 10). ABA and GA act antagonistically during seed development. ABA biosynthesis mutants produce more GA [54]. Three GA biosynthesis genes displayed higher expression in LGR S2 (early stage), one to two GA degradation genes had higher expression in all SN stages, and two to three GA responsive genes showed higher expression in SN S1 and LGR S3–S5 stages (Fig. 7C, and Additional file 1: Figure S10C). Hence, genes related to GA synthesis and response were promoted in LGR S2 and S3 stages, respectively, while degradation-related genes were promoted in SN. Genes related to ethylene pathway had five to seven biosynthesis genes with higher expression in SN S1–S4 and LGR S1 and S5; eight to seventeen signal transduction genes in all SN stages, with 17 genes in SN S3; four to five and three to four responsive genes in LGR S1–S5 and SN S1–S2 (Fig. 7D, and Additional file 1: Figure S10D). This suggests prominent ethylene signaling in SN S1–S3 and LGR S4–S5 (Fig. 10). Few pathway genes related to BR and CK, JA and SA showed difference in expression patterns and levels (Fig. 7E–H). Hence, genes which group into different co-expression clusters, and have higher expression in either SN or LGR, may be responsible for tweaking GS.

### Expression of known genes supports the transcriptome data

In the present study, the comparative expression patterns and levels of various genes has been considered as the basis for hypothesizing their roles in control of GS. To support this data, expression patterns of 42 functionally validated genes, including 35 positive and seven negative regulators of grain length, width, and weight were studied in the two genotypes [55, 56] (Additional file 1: Figure S11). Majority of the genes showed difference in expression patterns and/or levels in the five seed developmental stages between the two genotypes indicating their involvement in seed size regulation by performing similar functions at different time points. This meant that the difference in the time frame of seed developmental processes in SN and LGR was maintained. These genes include two transcriptional activators that promote seed filling in rice, *RISBZ1* and *RPBF*. Both expressed from SN S2–S5 and LGR S3–S5, with higher expression in LGR (Additional file 1: Figure S11D; a, b). *RAG2*, which enhances GS and seed filling, had peak expression at a later stage in LGR (Additional file 1: Figure S11D; c). *GE*, a positive regulator of cell division and endosperm size in rice expressed from SN S1–S4 and LGR S1–S5, with higher expression in LGR (Additional file 1: Figure S11D; d). *OsGS9*, which promotes cell division showed peak expression in SN S1 and LGR S2. *OsNF-YC10*, which is known to express in later stages of seed development and promote cell proliferation that also showed higher expression in LGR (Additional file 1: Figure S11D; f).

*OsHAP3E*, a negative regulator of embryo development and seed size, expressed from SN S2–S3 and LGR S3, with higher expression in SN (Additional file 1: Figure S11D; g). Similarly, *OsARF4*, which negatively regulates seed size and weight had higher expression in all five seed stages in SN (Additional file 1: Figure S11D; h). Genes with similar patterns but higher expression levels in LGR included *OsFIE2* (Additional file 1: Figure S11D; i), a PcG gene that positively regulates seed filling in rice. *GW8/OsSPL16* (Additional file 1: Figure S11D; j) which enhances endosperm cell size; *OsSRT1* (Additional file 1: Figure S11D; k), which promotes starch accumulation in endosperm; *GS5* (Additional file 1: Figure S11D; l), a positive regulator of cell cycle and *OsUBP15* (Additional file 1: Figure S11D; m), a positive regulator of cell proliferation and seed size.

### Genes generated by transcriptome analyses are localized within QTLs governing GS trait

QTL mapping in 286 individuals of a mapping population (LGR × Sonasal) using 56,783 genome-wide single-nucleotide polymorphisms (SNPs) identified 88 QTLs with major as well as minor effects controlling grain length, grain width, grain length/width ratio, and grain weight in rice (Additional file 2: Table S10). All these detected QTLs were mapped on 12 chromosomes explaining 1.5–30% phenotypic variation for grain size/weight with a significant logarithm of the odds score (LOD) (2.5–13.0) in rice. Further, genes from each of the abovementioned transcriptome analysis identified to delineate pathways controlling rice GS differentiation, which were either stage-specific or were highly differentially regulated between SN and LGR (Fig. 9, Additional file 2: Tables S5, S6, S7, S8 and S9), were overlayed with the grain size/weight QTLs. A total of 186 of these genes (Additional file 2: Table S11) were found to be located within QTL genomic intervals. These were contributed by 31 genes which were commonly differentially regulated in a stage-specific manner in SN and LGR, 15 cell cycle-related genes which were specifically upregulated in either SN S5 or LGR S3 stage, 38 LGR S3 preferential genes, 45 TFs belonging to families with stage-preferential expression in a stage of either SN or LGR, 50 phytohormone related genes which had a different pattern and level of expression between SN and LGR, and seven genes with opposite regulation in SS or CS comparisons whose upstream miRNAs also show opposite regulation. These 186 genes (including regulatory genes) present in the QTL genomic regions associated with GS traits give a strong indication of their roles in the process, and thus should be explored especially as promising candidate genes by their detailed functional characterization in rice.

### Novel miRNA-target modules in rice GS regulation

In rice seeds, miRNAs are known to function both in early seed development [57] and grain filling [58]. Using exactly the same seed development tissues (S1–S5) and flag leaf, as for transcriptome, miRNA expression profiles were generated for SN and LGR. Small RNA sequencing generated a total of 14 Gb clean data for 36 samples (Additional file 2: Table S12) with average Pearson’s correlation of 0.85 between biological replicates (Additional file 2: Table S13). miRNAs with TPM ≥ 50 [59, 60] were counted as expressed. The data was validated by stem-loop qRT-PCR of miR530-5p (Additional file 1: Figure S12A). A total of 193 (SN) and 196 (LGR) miRNAs were expressed, belonging to 70 and 76 families, of which four and 10 families were specific to SN and LGR, respectively (Additional file 1: Figure S12B, Additional file 2: Table S14). The numbers of total and specific miRNAs expressed in each stage were more in LGR (Additional file 1: Figure S12C). This was in conjunction with lesser number of expressed genes in LGR (Additional file 1: Figure S3). LGR also had a greater number of differentially expressed miRNAs (DEMs) than SN (Additional file 1: Figure S13A). Comparison of DEMs amongst CS and same SS stages of SN and LGR showed that most miRNAs exhibited similar regulation between SN and LGR in both comparisons (Additional file 1: Figure S13B, Additional file 2: Table S15).

Further, miRNAs and their targets with negative correlation in expression patterns were identified. DEMs present throughout seed development in SN or LGR and their targets were delineated to extract miRNA-target modules pertinent to GS (Additional file 1: Figure S14A, B, Additional file 2: Table S16). Thirteen miRNAs which were upregulated in all the five stages in SN had 85 targets which were downregulated in all stages in SN (Additional file 1: Figure S14A). These included osa-miR1848, osa-miR319a-3p, osa-miR535-3p, osa-miR1872, osa-miR1874, and osa-miR5504, which have been discussed later. Conversely, in SN itself, 13 miRNAs which were downregulated in all the five stages had 41 targets with negatively correlated expression patterns (Additional file 1: Figure S14B, Additional file 2: Table S16). This set included osa-miR1432 and osa-miR397, which have been discussed. Targets of these 13 miRNAs belonged to functional categories including cell wall synthesis, amino acid metabolism, TFs, protein post-translational modification, signaling, and transport. In LGR, 21 miRNAs that were upregulated in all the five stages had 61 negatively correlated targets (Additional file 1: Figure S14A, Additional file 2: Table S16). Majority of these were non-conserved miRNAs (specific to rice). These included osa-miR529 and osa-miR396, which have been discussed. Targets of these 21 miRNAs belonged to the functional categories including amino acid metabolism, hormone metabolism, secondary metabolism, TFs, stress response, signaling, and transport. Twenty-two miRNAs were downregulated in all the five stages of LGR and had 38 targets (Additional file 1: Figure S14B, Additional file 2: Table S16). In this set, osa-miR166, osa-miR398, osa-miR159, osa-miR167i-3p, and osa-miR172a have been discussed. Targets of these 22 miRNAs belonged to several functional categories relevant to GS, including cell cycle, cell division, cell wall modification, cell organization, DNA synthesis, fermentation, and starch synthesis.

**Fig. 8:**
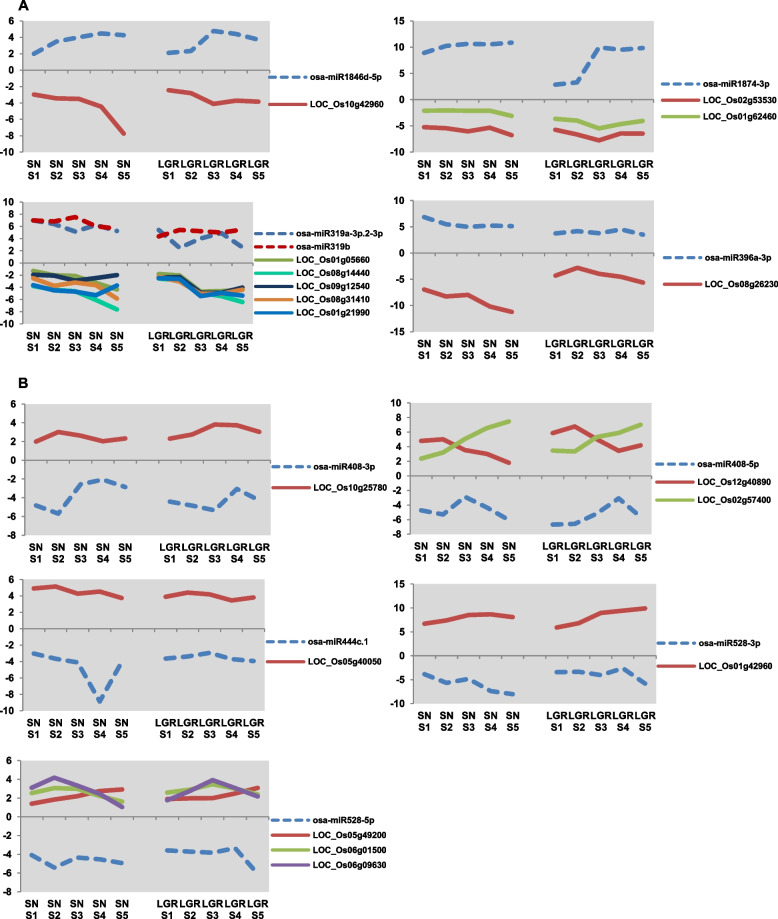
Expression analysis of miRNAs-targets pairs showing negative correlation in all five seed developmental stages of both SN and LGR. **A** Graphs showing differential expression levels (log_2_fc) of five miRNAs upregulated and their targets downregulated in all five stages of SN and LGR. **B** Graphs showing differential expression levels (log_2_fc) of five miRNAs downregulated and their targets upregulated in all five stages of SN and LGR. In each graph, dotted and solid lines represent miRNAs and their targets, respectively. Names of miRNAs and their targets have been mentioned in the legends in each graph

Five miRNAs were upregulated throughout seed development in both SN and LGR (Fig. 8A). They had a total of nine targets which showed negative correlation in expression pattern and were downregulated throughout seed development in both SN and LGR. Similarly, five miRNAs were downregulated throughout seed development in both SN and LGR (Fig. 8B), and targeted eight genes having negative correlation in expression. These targets belonged to functional categories which included TFs, hormone-related genes, signaling and transport, amino acid metabolism, and light reaction. These included osa-miR396, osa-miR408-3p, osa-miR408-5p, osa-miR444, and osa-miR528, which have been detailed further on. Of these, osa-miR319 and osa-miR528-5p had multiple novel predicted targets.

**Fig. 9:**
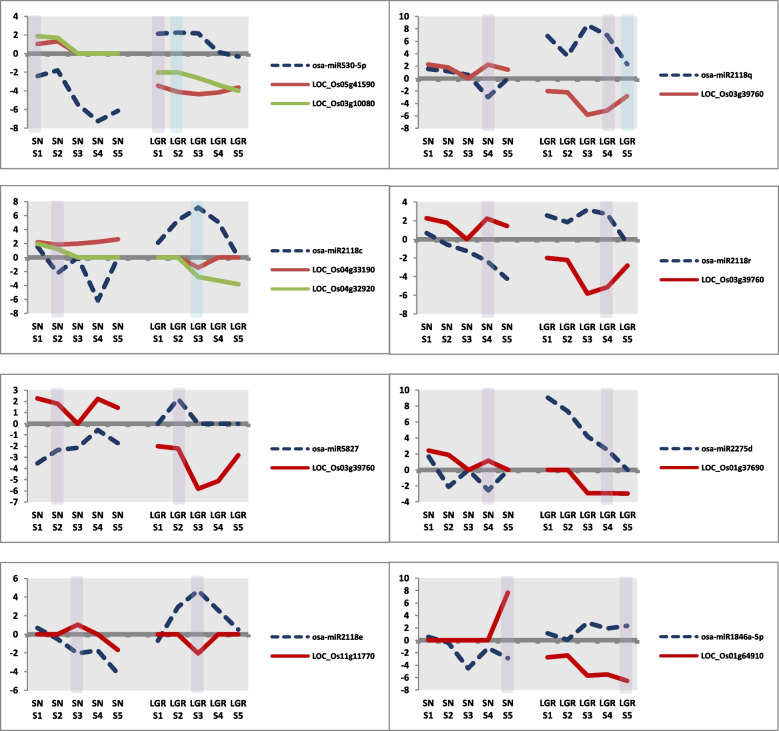
miRNA-target modules showing opposite regulation pattern in seed developmental stages of SN and LGR. Graphs showing differential expression levels (log_2_fc) of eight miRNAs upregulated in same stage (i.e., SN S1-LGR S1, SN S2–LGR S2) or comparable stage (i.e., SN S1-LGR S2, SN S2-LGR S3, SN S3-LGR S4, SN S4-LGR S5) of LGR and downregulated in same or comparable stage of SN, and their targets that show negative correlation in SN and LGR (downregulated in LGR and upregulated in SN in the same stage or comparable stage). IDs of miRNA and targets have been mentioned in the legends in each graph. Dotted blue line and solid lines represent expression of miRNA and targets, respectively. In each graph, negative correlation obtained between miRNA and its target in same stages and comparable stage has been marked with purple and blue bars, respectively

DEMs with opposite regulation between SN and LGR should also be important players in GS (Additional file 1: Figure S13B). In terms of SS, a total of 22 miRNAs were upregulated in any stage in LGR and downregulated in the same stage in SN. Similarly, five miRNAs were upregulated in any stage in SN and downregulated in the same stage in LGR. In terms of CS, nine miRNAs were upregulated in LGR and downregulated in a comparable stage in SN, while six miRNAs were regulated in a vice versa manner. Out of these, for eight miRNAs that were upregulated in LGR and downregulated in SN, nine targets showed negative correlation in expression (Fig. 9). A prominent family which was highlighted by this analysis was osa-miR2118, which has been detailed later. No targets were found showing negative correlation for miRNAs upregulated in SN and downregulated in LGR. The targets for eight miRNAs upregulated in LGR and downregulated in SN included two genes each for lipid metabolism genes and transport, and one gene each related to cytochrome P450, UDP glucosyl and glucoronyl transferases (UGTs), and biotic stress. Therefore, these miRNA-target modules exhibiting opposite behavior amongst LGR and SN might be essential regulators of GS in rice and should be examined further.

### Rice grain development database (RGDD) allows for easy data access

To make the transcriptome and miRNome data searchable, RGDD (www.nipgr.ac.in/RGDD/index.php) has been developed. In the transcriptome tab, Locus ID from RGAP (Rice Genome Annotation Project) can be directly entered in option 1. In case the Locus ID is not known, and the user wants to search using gene functions, then option 2 should be used. In this case, search can be made using options of the gene encoding for a TF or related to a phytohormone. Transposable elements can also be selected for or eliminated using option 2. The output shows both expression (average FPKM) and differential expression (Log_2_ fold change) values for all stages of both SN and LGR. The output also mentions if the gene is seed-specific or not (Additional file 1: Figure S16A). In the miRNome tab, option 1 allows for entering of a known miRNA ID. Option 2 allows to select an miRNA from a dropdown list. The output displays both the expression (average TPM) and differential expression (log_2_ fold change) values (Additional file 1: Figure S16B).

## Discussion

### Contrasting genotypes, SN and LGR

In the present study, two rice genotypes contrasting for GS have been considered for transcriptome and miRNome analyses, throughout five seed development stages, with the aim to elucidate genes and pathways contributing to increase in GS. In such extensive studies, there is a possibility of data dilution by genes reflecting genotypic differences. In order to minimize this, flag leaf for both genotypes has been used as a control to calculate DEGs involved in seed development, individually for SN and LGR. Subsequently, these DEGs have been compared amongst SN and LGR to elucidate the ones with different expression patterns and levels. Here, flag leaf has served as a control to remove genes which might have expressed at high levels in both leaf and seed in a particular genotype only. If not eliminated by this method, these genes would have been highlighted as DEGs and would be due to genotypic differences, and not represent an actual role in GS. The comparative methodology used in the present study has often been used to examine two plant varieties contrasting for a given trait. Temporal transcriptome analysis for cold tolerance, in two contrasting cultivars of tobacco, Tai tobacco (TT, cold susceptibility) and Yan tobacco (YT, cold resistance), has been used to identify DEGs in both cultivars after comparing with the corresponding control (without cold treatment) for each cultivar [61]. Comparative transcriptomics study in the root and shoot tissues of N-efficient (PBW677) and N-inefficient (703) cultivars of wheat have revealed the genes that regulate nitrogen use efficiency [62]. PBW677 has considerably more abundant DEGs compared to PBW703. Two contrasting peanut cultivars, Zhonghuahei 1 and Zhongkaihua 151, with high and low free amino acids in mature seeds, respectively, have been compared by metabolomics and transcriptomic approaches to identify the regulatory network of amino acid metabolism [63]. Recently, a study integrated metabolome with transcriptome analyses in order to have a better understanding of the metabolite profiles and molecular mechanisms regulating different cane traits, namely, brix, rind color, and textures in the stems and leaves of contrasting sugarcane varieties FN41 and 165402 [64]. Two contrasting teak cultivars, *T. grandis* “Xifei” and *T. grandis* “Dielsii,” with distinct oil content were used for transcriptome analysis to unravel profile of fatty acid accumulation during kernel development [65]. In light of all these, cultivars contrasting for a particular trait can be compared by transcriptome analyses to elucidate target genes controlling the trait under consideration.

The other imperative question that emerges from such a comparative analysis is the genetic background of the two varieties. A phylogenetic analysis of the genomic sequences of SN and LGR [66] shows that though they both fall in the major clade of *japonica* rice, SN lies at the edge of an aromatic sub-clade and LGR lies at the edge of a temperate sub-clade. Both SN and LGR are quite dissimilar to core *japonica* members, such as Nipponbare. SNPs in SN and LGR are mostly intergenic, and a large number of SNPs is shared between the two. This implies that SN and LGR can be compared for their contrasting grain traits, and the genes identified from this study can be used for functional analyses in future studies. The localization of 186 highly differentially expressed genes within QTL sequences governing various GS traits further validates our study and provides evidence that these genes may actually control GS and are not products of genotypic differences. Moreover, whole seeds, including the husk, embryo, and endosperm, have been used in the study. This is because the aim was to elucidate the overall scenario of genes, pathways, and events which regulate GS. Such an analyses are subsequently followed with detailed characterization and functional validation by generation of rice plants with altered expression of target gene/s, which can precisely identify the exact role of the gene/s in the trait. Hence, global transcriptome analysis of whole seed can be used for studying genes controlling GS. In line with the effectivity of this methodology, recently, a protocol has been developed to specifically examine both *indica* and *japonica* whole grains using an Agilent microarray platform [67]. Transcriptome of whole rice grains has been used to study grain filling under soil drying conditions [68]. Entire panicles have been used for studying transcriptome rhythms due to warm night temperature [69]. Transcriptome analyses have been performed on mutant and wild-type 12 DAP whole grains to understand role of *Fernonia-like receptor* genes in GS [70]. Complete caryopsis has been used to examine the transcriptome and phytohormone changes contributing to grain chalkiness [71]. These studies support the fact that the whole seed of contrasting genotypes SN and LGR can be used for comparative transcriptome analyses to elucidate genes regulating GS. Though the actual role of the genes emerging from this study can be confirmed upon functional validation, the data presented should be able to be extrapolated to other rice varieties. This is because the genes which have been priorly known for their roles in the control of GS (Additional file 1: Figure S11) and have their expression levels and patterns amongst SN and LGR, in conjunction with their roles (Additional file 1: Figure S11D), are from an array of rice genotypes. The genotypes in which the roles of these genes in GS control have been elucidated are Kitaake for *RISBZ1*, *RPBF*, and *OsUBP15*; Zhonghua 11 for *RAG2*, *OsNF*-*YC10*, *OsNF*-*YB7*, and *OsFIE2*; Taichung 65, Nipponbare, Zhonghua 11, and Hejiang 69 for *GE*; Nipponbare for *OsGS9* and *OsARF4*; *indica* Basmati 385 for *OsSPL16*; Minghui 63 for *OsSRT1*; and Zhenshan 97 for *GS5* [11, 23, 72–78]. Since 13 genes have concomitant expression as their role in GS, amongst SN and LGR, in eight different genotypes, it can be hypothesized that the roles of the genes enlisted in the present study could represent a general situation in rice. A gene emerging as essential for a particular genotype, more often than not, also behaves similarly in another genotype. A recent example for this is *GW2*, which has been well characterized to be a negative regulator of GS in *japonica* rice [79]. Despite the regulatory SNP being absent in *indica* rice, this gene functions in a similar manner [80].

Amongst DEMs during entire seed development in both SN and LGR (Fig. 8A), miR1846 was discovered as a novel miRNA in *japonica* cultivars Zhonghua 11 and Nipponbare developing seeds [57, 81]. miR1874 was upregulated throughout seed development in both SN and LGR (Fig. 8A). It specifically expresses in developing seeds of both *japonica* cultivar Nipponbare [82] and Baifeng B, an *indica* landrace [83]. Similarly, miR528 which is highly expressed in Nipponbare grains [81], is also highly expressed in both SN and LGR (Additional file 2: table S14). Another case is of miR396, which is a negative regulator of GS in different genetic backgrounds in both *indica* and *japonica* rice [84]. Hence, it will be interesting to extrapolate the information generated in the present study to other genotypes and identify molecular markers of rice grain development and GS control.

### Transcriptome changes regulating GS

Transcriptome transitions control developmental progression. The morphological data of seeds and endosperm sections showed the full seed size and weight were obtained later in LGR (Fig. 1, Additional file 1: Figure S1). Developmental events also occurred later in LGR seed development. Hence, it can be hypothesized that slower progression of seed development might positively affect GS. To elucidate genes/processes responsible for this, the transcriptomes of SN and LGR during seed development were compared. Amongst the pathways with ≥ 50 genes with log_2_fc ≥ 10 (Additional file 1: Figure S4B), AGPs (under cell wall category) and GDSL motif lipases were more in LGR. AGPs are glycoproteins present on the cell wall and function in plant growth and development, including cell division and expansion. They express in various rice tissues, including seeds and seedling that are subjected to rapid changes in cell morphology [85, 86]. GDSL are multifunctional enzymes involved in fatty acid metabolism during seed germination and have been found to be downregulated in *RGE1* mutants of *Arabidopsis* which exhibit smaller seeds [87, 88]. Abundance of these proteins in LGR might indicate their involvement in cell size increment. SSPs and LEAs were abundant in both SN and LGR. This is because SSPs are second major storage products in rice [14, 89]. LEA proteins form up to 4% of total cellular proteins in seeds and are associated with imparting drought tolerance during seed drying phase [90, 91]. The comparative transcriptome data shows an insight into the genes and pathways that might be responsible for GS increment.

#### LGR S3 stage is important for increase in GS

CS and SS pair-wise comparisons for oppositely regulated DEGs, categorized into the same pathways (Fig. 2D). This could imply that similar functions could be modulated by different genes in the two genotypes. LGR had higher genes in the DNA synthesis category. DEGs had an LGR-specific enrichment of cell cycle and DNA metabolism (Additional file 1: Figure S6A), and the number of cell cycle-related DEGs were highest for S3 stage of LGR (Fig. 3B). This was the stage where the endosperm cell size increased in LGR (Fig. 1I, xv, xvi), unlike SN. DEGs preferential to LGR S3 (Additional file 1: Figure S15) had a high number of cell cycle-related genes. Also, 38 LGR S3 stage-specific or preferential DEGs were located within QTLs governing GS. S3 stage in LGR overlapped with endoreduplication phase during cereal seed development [92, 93]. Expression of endoreduplication-related genes peaked in LGR S3 stage (Additional file 1: Figure S6C). Direct correlations exist between endoreduplication and cell size in plants [94]. It is known that endoreduplication increases cell size by increasing nuclear volume, driving cells to increase cell volume to maintain nucleo-cytoplasmic ratio [95], and by increasing gene expression to enhance reserve accumulation [96]. Hence, it can be hypothesized that higher endoreduplication levels in LGR might be positively contributing to GS, which can be verified further.

The process of grain filling, once completely understood, can be used to increase yield [97]. After starch, the second most abundant nutrient component of rice grain are SSPs [98]. Quality of SSPs and starch have a direct influence on each other [99, 100]. The eating quality of rice is determined by starch and SSP composition [101], and rice flour is increasingly being looked at as a product to alleviate malnutrition [102]. Almost 50% SSPs expressed at very high levels in both SN and LGR with FPKM ≥ 500 (Additional file 1: Figure S15), as also observed in previous studies [14, 103]. Hence, understanding the pattern of the genes controlling reserve accumulation in SN and LGR will aid in correlating grain filling with GS. LGR seeds showed delayed starch biosynthesis and lesser starch yield (Fig. 4C, D). Though considerable starch content was seen in SN S2 stage, it increased exponentially in S3 stage of both SN and LGR. This was supported by expression patterns of sucrose synthase and carbohydrate metabolism genes (Fig. 4E, Additional file 1: figure S8A). Sucrose synthase genes preferably catalyze sucrose degradation in vivo and provide sugars for respiration and starch [104] and cellulose synthesis to mediate organ elongation in plants under different stimuli [105, 106]. Phosphorylation of fructose after cleavage of sucrose by fructokinases drives carbon metabolism towards starch synthesis and respiration. *OsFKII*, a rice fructokinase gene, expresses at high levels in endosperm [107] suggesting involvement in starch accumulation. Mutation in rice plastid phosphorylase gene, *Pho1*, reduces starch synthesis and causes abnormal seed morphology [108]. There are innumerable examples to show that GS and starch content have a direct correlation and starch has a forbearance on grain quality [109–111]. In SN and LGR seeds, the expression of *SSP*s (Fig. 5A) was validated by histological sections (Fig. 5B), protein gel (Fig. 5C), and total protein yield estimation (Fig. 5D). The highest increment in protein concentration between LGR S3 and S2 also supported our hypothesis of an intense transcriptome reprogramming in LGR S3 stage (Fig. 3). The key proteins in rice grain are glutelins and prolamins [112], which was also reflected in the protein gel (Fig. 5C). GS is directly correlated with grain filling, as indicated by NF-YC12 mutants [113]. Attempts are being made to identify QTLs which can positively contribute to the protein content of seed [114]. Hence, the genes related to carbohydrate biosynthesis and metabolism and *SSP* biosynthesis play an important role in control of GS and should be targeted for crop improvement through molecular breeding.

#### Regulators of GS

TFs regulate grain size and shape [55]. When the genes participating in/coding for TFs, hormone-related, cell cycle and growth, SSPs and carbohydrate pathway (Additional file 1: Figure S15) were sorted on the basis of their differential expression or specificity, amongst the seed-specific genes commonly expressed in all seed stages of SN and LGR, maximum genes were TFs indicating their regulatory importance during seed development. TF families (≥ 10 members) included Myb, AP2, NAC, zfC_2_H_2_, bZIP, Homeobox, MADS, B3, PHD, and Aux/IAA, in that order. For DEGs with log_2_fc ≥ 10, TF encoding genes were most abundant, again, highlighting their regulatory importance. Genes from many of these families regulate seed development [12, 37, 38, 51, 53]. Comparison of expression of TFs between SN and LGR showed that members of bZIP, NF-YC, C_2_H_2_, GATA, MADS, FHA, NF-YB, PHD, LBD, PLATZ, SBP, WRKY, and DOF families might regulate GS in various capacities and are also located within QTLs (Fig. 6, Additional file 2: Table S11). Of these, *RISBZ1*, a bZIP TF is involved in starch synthesis in rice seeds [115]. bZIPs regulate amylose biosynthesis in wheat grain [116]. NF-Ys regulate rice grain quality [117]and control endosperm development [118]. *OsNF*-*YB1* regulates nutrient transport via sucrose transporters in endosperm during grain filling [113, 119]. OsMADS14 and NF-YB1 interact with each other to regulate starch biosynthesis [120]. Many other MADS box TFs are known to regulate seed development, including MADS29 [50, 121]. C_2_H_2_ zinc finger TFs affect grain quality [122] and amylose content [111]. FHA domain-containing proteins are members of kinase-mediated signaling pathway, associated with DNA repair and cell cycle [123]. PHD finger TF members regulate transcription via chromatin remodeling/histone acetylation in animals and yeast [124, 125]. PHD and C_2_H_2_ zinc finger families exhibit epigenetic control [126]. Amongst TFs upregulated in SN and downregulated in LGR (Additional file 1: Figure S15), NAC (10 members) and Myb (9 members) were prominent. These included *OsMYB30* and *OsMYBS3*, modulating starch breakdown [127, 128]; *OsNAC4*, a positive regulator of hypersensitive cell death (a type of PCD) [129]; *OsSWN3*, a NAC TF involved with secondary cell wall formation [130]; and ABA responsive *OsNAC52* [131]. Regulation of cell cycle, cell expansion, grain filling, and epigenome-related genes differently by TFs, especially by members of families mentioned here, would be important in modulating GS in LGR.

Hormones play an essential role in endosperm development [132]. Amongst phytohormones, auxin, ABA, GA, and ethylene-related genes exhibited prominent opposite patterns of higher expression between SN and LGR (Fig. 7). Genes related to all phytohormones were located on QTLs governing GS (Additional file 2: Table S11). This data was supported by *SAUR39*, a negative regulator of auxin signaling in rice [133]. This gene had higher expression in early stages of LGR. Mutation in an auxin biosynthetic pathway gene reduces starch accumulation and seed size in pea [134]. A novel miR167a-OsARF6-OsAUX3 module indicates that auxin positively regulates grain length and width in rice [135]. Auxin also exerts a maternal regulation on seed size [56]. In SN and LGR, higher expression of auxin biosynthesis genes overlapped with the period of cell elongation and starch accumulation (Fig. 4C). Hence, the expression of auxin-related genes in SN and LGR indicated its role in cell size, starch production, and GS regulation. In our data, *OsNCED1*, an ABA biosynthetic gene, peaked in SN S3 but dropped in LGR S3. Reduced ABA levels delay cellularization and increase seed mass in *Arabidopsis* [136]. On the other hand, GA assists attainment of proper seed size in pea and *Arabidopsis*. It promotes endoreduplication and cell expansion. GA-insensitive rice mutants produce smaller seeds [137]. *OsGA3ox2*, a GA biosynthesis gene, had higher expression in LGR S1-S2. It regulates cell elongation under increased BR levels [138]. GA responsive gene, *OsGSR1*, which promotes BR biosynthesis and regulates cell elongation [139], showed higher expression in SN S1–S2 and LGR S3–S5. *OsGA2ox1*, which encodes for an enzyme catabolizing active GAs and aborts seeds on overexpression [140], expressed in SN S1 but not in LGR. This interplay of higher expression of ABA-related genes in SN and GA-related genes in LGR could contribute to differences in their GS. Inferior spikelets of rice have higher ethylene levels and exhibit reduced cell division, seed filling, and GS [141], and ethylene promotes maize endosperm PCD [142]. Higher activity of ethylene biosynthesis and signal transduction genes during early SN seed development probably accelerates PCD (Fig. 1I and Additional file 1: Figure S1C), thus reducing GS. Since the expression of phytohormone-related genes was in sync with their known roles in seed development, it can be hypothesized that the other genes listed in the study can be utilized in future studies to understand their roles in GS control.

### Certain miRNA-target pairs regulate the process of GS in SN and LGR

A higher miRNA number is related with increased seed filling and grain weight in superior rice spikelets, as compared to inferior spikelets [143]. Hence, more miRNAs were expressed in LGR (Additional file 1: Figure S12C) probably to optimize gene expression in favor of GS. This is also because resources for both plant vegetative growth and reproduction are limiting [144]. Hence, it can be implied that genotype-specific miRNA families (Additional file 1: Figure S12B) must be crucial for GS regulation. Amongst these, osa-miR1848, which promotes smaller GS, was specific to SN [145]. On the other hand, osa-miR397, which positively regulates GS, was specific to LGR [146]. This raised the possibility of an involvement of the other previously uncharacterized genotype-specific miRNA families such as osa-miR5806 and osa-miR3979 in regulation of GS trait in rice.

#### miRNA-target modules differentially regulated in all stages of seed development in a genotype

miRNAs upregulated in all stages of SN, with oppositely correlated targets (Additional file 1: Figure S14A) included osa-miR1848-*OsCYP51G3* module known to reduce seed size [145], corroborating relevance of the identified modules in seed size regulation. Some of the miRNAs in this set regulate abiotic and biotic stress responses, including osa-miR319a-3p, osa-miR535-3p [147], osa-miR1872, osa-miR1874, and osa-miR5504 [148], but their role in seed development has not been identified yet. Conversely, the other set of miRNAs which were downregulated in all stages of SN included osa-miR1432 and osa-miR397, which are known regulators of seed size in rice [58, 146]. Another example for SN is osa-miR1848-*OsASR6* module which optimizes auxin levels to reduce cell enlargement and starch accumulation [149]. miR1432 is known as a negative regulator of grain filling by targeting *OsACOT* [58]. It works through another module with EFH1 to negatively control blast resistance [150]. However, in our data, miR1432 was downregulated in all stages of SN (Supplementary table 13). The five predicted targets for miR1432 (Supplementary table 13), with negative correlation of expression, have not been characterized previously. Hence, it is possible that miR1432 might be controlling grain size in SN through these new modules, which need to be characterized further. This also implies that many new predicted miRNA-target modules in this data should be validated for their roles in grain development.

In LGR, miRNAs upregulated in all stages (Additional file 1: figure S14A) had rice-specific miRNAs which are known to primarily exhibit low or tissue-specific expression in developing rice grains [81]. Conserved miRNAs included osa-miR529 and osa-miR396, which are involved in seed size regulation [151, 152]. In the contrasting set of miRNAs downregulated in all stages of LGR, several miRNAs associated with seed size and grain filling were present, including osa-miR166 [153], osa-miR398, and osa-miR159 [154]. Few of the targets were cyclin-related proteins (targeted by osa-miR167i-3p) and FtsZ2-*1* (targeted by osa-miR172a). *OsFtsZ2* is homologous to bacterial cytokinesis-related FtsZ and is implicated positively in amyloplast division in rice [44]. There was also an expansin precursor, which mediates cell wall expansion [155]. These miRNAs are downregulated throughout seed development in LGR to enhance GS by promoting numerous aspects of seed development, including cell proliferation, cell elongation, energy production, and grain filling [9, 44, 96, 134]. Thus, new potential miRNA-target modules identified here should be explored further to establish their roles in GS regulation.

#### miRNA-target pairs with similar differential regulation in both SN and LGR

miRNA-target modules with similar regulation throughout both SN and LGR (Fig. 8) can be pivotal regulators of seed development. miR396a-3p is one such molecule. There are 9 members in osa-miR396 family. Of these, miR396c, miR396e, and miR396f are negative regulators of grain length, width, and weight [156, 157] while miR396b negatively affects grain yield, but does not affect 1000-grain weight, a direct indicator of grain size [158]. MIM396-5p plants, where all of the above are downregulated, have increased grain length but decreased grain width [84]. Grains of Baifeng B (an *indica* landrace) and *japonica* cultivar Zhongua 11 show high expression of miR396 [57, 83]. In SN and LGR, miR396a-3p was upregulated throughout grain development, though to a higher extent in SN (Fig. 8A). Its putative target is novel. Hence, miR396a-3p might be an important regulator of rice seed development and should be explored further. Also, in Arabidopsis leaf, the levels of miR396 limit cell proliferation [159], indicating a similar role in grain. Additionally, in our data, 13 miRNAs belonging to this family (including -3p and -5p forms) are differentially expressed, and regulation of GS might be a combination of these. miR396 functions through miR408, a positive regulator of grain size [84]. In concordance, both miR408-5p and miR408-3p were downregulated throughout grain development in SN and LGR (Fig. 8B). However, it is known that osa-miR408-3p targets constitutively expressed *BRD2*/*LTBSG1* and is involved in BR-mediated cell elongation in rice seeds [160]. Often genes regulate multiple aspects of rice grain development, including grain size, starch, and seed storage protein biosynthesis. Since miR396/miR408 module is differentially regulated throughout seed development, and with novel targets, it is possible, these miRNA-target pairs are major regulators of rice grain development, and need to be explored further. In addition, osa-miR319b which was commonly upregulated during seed development in SN and LGR (Fig. 8A) is known to target *OsCAF2*, which regulates normal chloroplast development [161]. This suggests suppression of chloroplast development in rice seeds. miR319 has been shown to target TCPs in leaf development [162]. It is highly upregulated in our data (Fig. 8A), and with novel targets, hinting at new avenues to explore. miR444 is known to regulate tillering and ovule development [163, 164]. It is downregulated in both SN and LGR (Fig. 8B) though to varying extents. miR528 is known to control flowering time, pollen formation, and plant height [165–167]. With the prediction of novel targets in seed (Fig. 8B), its function should be examined here.

#### miRNA-target modules with opposite differential regulation between SN and LGR

Amongst the targets of miRNA-target modules with opposite expression between SN and LGR, cytochrome P450 family members are known to regulate GS in rice via BR-mediated cell expansion [168–170], suggesting their relevance in GS regulation. miRNA targets also included lipid metabolism genes one of which was an acyl CoA ligase/synthetase and another a glycerol-3-phosphate dehydrogenase. Acyl CoA ligases express in developing rice seeds and provide substrates for triacylglycerol (TAG) synthesis, which are major storage lipids and energy source [171, 172]. GPDH is involved in energy production from gluconeogenesis. Inhibition of sucrose production via gluconeogenesis triggers compensated cell enlargement in *Arabidopsis* cotyledons [173], and hence, these targets might have a role in GS regulation. Four members of miR2118 family and their targets were oppositely regulated between SN and LGR in either CS or SS stages (Fig. 9). miR2118 is essential for proper reproductive development by formation of anther wall [174]. Since its members are upregulated in LGR stages, this family can be studied further for their role in GS control.

Briefly, it appears that genes associated with BR signaling, cell expansion, and stress tolerance experience opposite regulation, via miRNAs, in SN and LGR. Furthermore, miRNAs with opposite regulation between SN and LGR, seem to favor cell enlargement by modulating BR signaling and lipid metabolism [168, 173], thereby increasing GS in LGR. In addition, the localization of these targets on QTLs associated with GS strengthens the probability of their involvement in the process. Thus, novel miRNA-target modules identified for SN and LGR can provide suitable avenues for future studies in rice GS regulation.

### The Domino effect model of seed size regulation

**Fig. 10:**
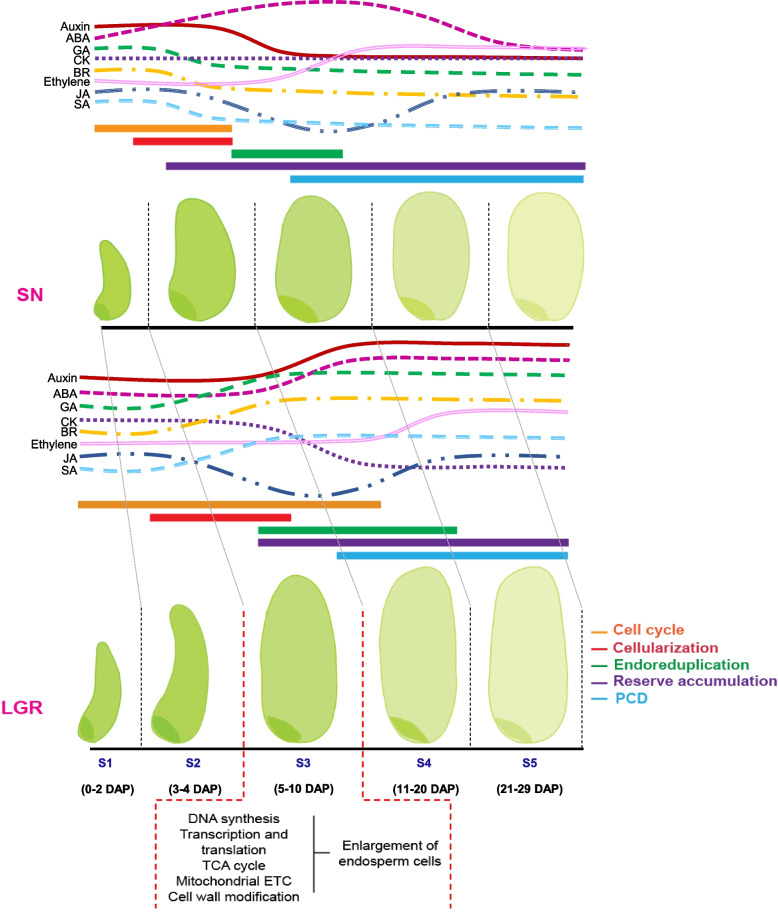
The “Domino effect” model of seed size regulation. Diagram represents progression of the major seed developmental events in SN and LGR. Upper and lower panels represent SN and LGR, respectively (as mentioned on the left of the diagram). S1–S5 represent five stages of seed development. Orange, red, green, violet, and blue bars represent progression of cell cycle, cellularization, endoreduplication, storage reserve accumulation, and PCD during seed development (as indicated in the color legend). Lines represent accumulation pattern of hormones during seed development in SN and LGR based on genes showing different expression pattern in the two genotypes. Pathways specific or preferential to S3 stage in LGR contributing to enhanced cell size have been mentioned between the dotted red lines below S3 stage. Comparable seed developmental stages have been connected with dotted gray lines

This extensive comparative morphological, histological, and transcriptome analysis of SN and LGR seeds throughout seed development concludes with a “Domino effect” model of seed size regulation (Fig. 10), emphasizing significance of the chronology of seed developmental events in governing GS. Just as falling of one domino triggers the next one, completion of one seed development event initiates the next. This process is strictly overseen by TFs, hormonal interplay, and miRNA regulation. Stage-wise comparison of the transcriptome of five seed stages showed that any given stage of LGR was most similar to the preceding stage in SN, also validated by endosperm sections. Delayed cellularization in LGR indicated a longer period of initial cell division phase (Figs. 1 and 2), supported not only by enrichment of cell cycle-related genes in its transcriptome, but also continuation of cell cycle till S3 stage (Fig. 3). Maximum increment in seed weight in SN and LGR occurred in S3 and S4 stages, respectively, indicative of delayed storage reserve accumulation in LGR (Figs. 1H, 4 and 5), as a consequence of predominant expression of carbohydrate and SSP biosynthetic genes from SN S2 and LGR S3 stages onwards. Storage reserve accumulation occurs after cellularization [13]and enhances seed size and weight [23, 119]. As cellularization is prolonged in LGR, accumulation of storage compounds is procrastinated (Fig. 10). Cells in LGR spikelets were larger in size (Fig. 1; Additional file 1: Figure S1A), suggesting enhanced cell expansion. Subsequently, enhanced endosperm cell size and prominent nuclei were apparent in S3 stage, marking it as the period of cell elongation, coinciding with endoreduplication phase [12]. Markers of endoreduplication showed peak expression at LGR S3 (Additional file 1: Figure S6C). Additionally, LGR S3 transcriptome was most unique in comparison with other stages (Figs. 2B and 3), signifying intense transcriptome reprogramming to increase seed size. Thus, larger seed size in LGR appears to be a result of enhanced cell expansion via endoreduplication during S3 stage (Fig. 10). Lastly, PCD started early in SN endosperm cells (Fig. 1I and Additional file 1: Figure S1C), induced probably by expression of positive regulators of PCD from S2 stage onwards (Additional file 1: Figure S8B and C). Precocious cellularization and PCD are known to reduce GS in rice [25, 26], thus restricting GS in SN, and in turn supporting the postulated “Domino effect”. Essential events occurring during seed development, namely cell cycle, cell expansion, storage accumulation, and PCD, appear to be modulated by temporal regulation of phytohormones in the two genotypes creating differences in GS (Figs. 7 and 10). Moreover, extensive miRNA regulation of genes throughout seed development (Fig. 8) adds another level of regulation in this process. Our study also suggests the presence of new miRNA-target modules that need to be functionally validated for their roles in rice seed development. The postulated Domino effect model is also supported by the transcriptome analyses of seed development in IR64, which has a medium-sized grain [14]. Thus, a “Domino effect” influences seed development wherein one process/pathway is overlapped by the next one, and it is the extent of one process that determines the occurrence of subsequent one, thereby regulating seed size.

## Conclusions

Comparison of transcriptome of five seed development stages from SN and LGR highlights the importance of S3 (5–10 DAP) stage in LGR, for increment of rice grain size. S3 stage of LGR has the most unique transcriptome amongst all comparisons. This is the stage where maximum number of cell cycle genes specifically express, and the increment in total protein content is highest. All events of seed development, including grain filling, occur later in LGR. Genes involved in phytohormone pathways (136 genes) and members from nine transcription factor families contributing to temporal changes have been elucidated. The DEGs underlying the QTLs will have functional relevance for genetic dissection of GS trait in rice. Novel miRNA-target pairs which might contribute to seed development or GS increment have been determined. Out of these five miRNAs show upregulation throughout seed development in both SN and LGR and target nine genes. Also, five miRNAs are downregulated throughout seed development in both SN and LGR and target eight genes. Eight miRNAs and their nine targets have opposite regulation between SN and LGR and could potentially regulate GS. The analyses have led us to propose a “Domino effect” model for rice grain increment. In this, the attainment of completion of one step of grain development triggers the next one. Since each event is slower in LGR, there is a temporal lag leading to increased cell size and subsequently higher grain filling, and eventually bigger grain size. The expression data for all genes and miRNAs from this study are available to RGDD. Availability of such information on a single platform will not only be useful for rice yield enhancement but can be extrapolated to other crops as well.

## Methods

### Plant materials

Long-grained and short-grained *indica* rice, LGR [66], and Sonasal (SN), respectively, were grown in the kharif season in the field conditions at NIPGR, New Delhi, India. Once panicle emergence started, individual panicles were observed for anthesis. Panicles took 3–4 days to complete emergence and anthesis. Each panicle on the plant was tagged on the day of its anthesis (by mentioning the date) before noon, particularly the region where freshly dehisced anthers are visible, as the process follows a basipetal direction. The pollinating spikelets on the lowermost part of the panicle were often left untagged, due to staggered anthesis. The day of anthesis was counted as 0 DAP, as pollination occurs in a few hours. The tagged panicles were left on the plant to mature. Individual seeds were separated and collected for each DAP, as they matured and harvested in liquid nitrogen. While harvesting, empty seeds were discarded. Seeds from each DAP were stored at − 80 °C. At the time of RNA isolation, equal weights of seeds for each DAP were pooled according to the categories, S1 (0–2 DAP), S2 (3–4 DAP), S3 (5–10 DAP), S4 (11–20 DAP), and S5 (21–29 DAP). Each biological replicate was made from a separate pool of seeds. Dried mature seeds of SN and LGR were harvested for estimation of grain length, width, and weight.

### Seed trait measurements

Freshly harvested seeds of 0, 1, 2, 3, 4, 7, 10, 13, 18, 21, and 29 DAP were collected in 30% ethanol. Images of husked and dehusked seeds of SN and LGR were captured at × 4 magnification under a stereozoom microscope (AZ100, Nikon; Japan). Grain length and width were quantified per seed by taking average of 100 seeds using WinSEEDLE™ software (Regent Instruments Inc.; Canada). One thousand-grain weight was measured in biological triplicates. Grain filling rate was estimated by measuring fresh weight of 15 seeds in triplicates from each DAP after removing stalks and awns. The measurements from each DAP constituting a stage were added to obtain the weight of 15 seeds/stage.

### SEM analysis of spikelet

Freshly harvested spikelets belonging to 7 DAP harvested from SN and LGR were immersed completely in FAE fixative solution [10% formaldehyde, 5% acetic acid, and 50% absolute ethanol] in 15 ml Borosil® glass vials. These samples were vacuum infiltrated for 30 min and incubated at 4 °C overnight in dark. The tissue samples were subjected to dehydration series with increasing gradient of ethanol (60%, 70%, 80%, 95%, and 100%) at room temperature for 1 h. The outer surface cells of lemma were observed under EVO® LS10 scanning electron microscope (ZEISS, Germany) at × 500 magnifications. The images were analyzed using ImageJ [175] to estimate cell length and cell area of the outer epidermal cells in the middle region of the lemma.

### Histological study

Freshly harvested seeds of 4, 5, 7, 9, and 11 DAP were dehusked, fixed, and dried in ethanol series (as mentioned in the previous section). Ethanol was gradually replaced with xylene by keeping the tissues in increasing concentration of xylene and decreasing concentration of ethanol in the percentages of 25:75, 50:50, and 75:25. Next, two rounds of treatment with 100% xylene were given to the seeds at room temperature for 1 h on the rocker, followed by infiltration with paraplast X-TRA (Sigma-Aldrich; USA) by adding molten paraplast every 2–3 h at 60 °C for 3 days. The wax-infiltrated tissues were then embedded into molds (Yorko®; India), and 10-μm sections were cut for 4, 5, and 7 DAP and 15-μm sections were cut for 9 and 11 DAP using rotary microtome (Leica Biosystems, Germany). Paraplast was removed from the sections by immersing the slides in HistoChoice® clearing agent (Sigma-Aldrich; USA) for 1 h. Sections were stained with 0.01% toluidine blue-O for 10 min or 0.1% Coomassie brilliant blue/CBB [0.25% CBB in 50% methanol and 10% acetic acid] for 20 min or 2% I2/KI solution (2 g KI and 0.2 g Iodine in 100 ml MQ) for 2–5 min. The sections were mounted using D.P.X. mountant (Himedia®, India) and were visualized under light microscope (Eclipse 80i, Nikon; Japan) at × 20 magnification.

### Total RNA isolation from seed tissue and flag leaf

Total RNA was isolated from five seed developmental stages of the two rice genotypes LGR and SN, namely, S1 (0–2 DAP), S2 (3–4 DAP), S3 (5–10 DAP), S4 (11–20 DAP), and S5 (21–29 DAP) using a seed-specific protocol [176] with few modifications, as previously described [37, 52, 80, 177]. Briefly, 100 mg tissue was taken by pooling equal amounts of seed tissue from the respective DAP constituting a stage. The tissue was ground to a fine powder in liquid nitrogen and mixed with extraction buffer [50 mM Tris–HCl pH 9.0, 20 mM EDTA pH 8.0, 150 mM NaCl, 1% N-lauryl sarcoyl (sodium salt), and 5 mM DTT], followed by phenol:chloroform:isoamyl alcohol (25:24:1) treatment. Then, GH buffer [8 M guanidine hydrochloride, 20 mM 2-[N-Morpholino] ethanesulfonic acid, 0.5 M EDTA, 50 mM β-mercaptoethanol] and phenol:chloroform:isoamyl alcohol were added to the supernatant, followed by chloroform treatment. RNA was precipitated by adding 3 M sodium acetate (pH 5.2) and twice volume of chilled ethanol. The pellets were washed twice with 70% ethanol, dried, and dissolved in 40 μl DEPC-treated deionized water. RNA from flag leaves of SN and LGR were isolated using TRI Reagent® Solution (Invitrogen™; USA) as per manufacturer’s protocol. DNase treatment was given to the RNA samples using RNase-Free DNase Set (Qiagen; Germany). The purity and concentration of RNA samples were checked by NANODROP 2000c Spectrophotometer (Thermo Scientific; USA) and on 1% denaturing agarose gel in 1 × MOPS buffer [400 mM MOPS, 99.6 mM sodium acetate, 20 mM EDTA] with 1.1% formaldehyde. Integrity of RNA samples was checked using 1 μl RNA sample (25–500 ng/μl) by Agilent 2100 Bioanalyzer.

### RNA-Seq library preparation and sequencing

The RNA isolated from the seed and the leaf samples (in biological triplicates) were used for cDNA library preparation according to the TrueSeq® RNA Sample Preparation v2 Guide (Illumina®; USA) according to the manufacturer’s protocol as described previously [53, 80]. Briefly, poly-A mRNA purified using oligo(dT)-attached magnetic beads were fragmented and primed, followed by double-stranded cDNA synthesis using SuperScript II Reverse transcriptase (Invitrogen™; USA), Second Strand Master Mix, End Repair Mix and Resuspension Buffer. Blunt-ended ds cDNA fragments were then adenylated and adapters were ligated. Following this, DNA fragments were enriched by PCR and paired-end sequencing was performed using Illumina HiSeq™ 2000.

### Whole transcriptome sequencing data analysis

Data analysis was done as mentioned earlier [53, 80]. Low-quality reads were trimmed using Cutadapt (v1.8.1) (Martin, 2011). The unwanted sequences, including non-polyA tailed RNA, rRNAs, tRNAs, adapter sequences, mitochondrial genome, were removed using Bowtie2 (v2.1.0) (Langmead, 2010), in-house perl scripts and picard tools (v1.85). The clean reads thus obtained were used for expression and differential expression analysis. The clean reads were aligned to the reference genome (MSU 7) by TopHat (v2.0.8) [178]. The uniquely mapped reads were used for estimation of gene expression using Cufflinks program (v2.0.2) [179]. Pearson’s correlation coefficient and PCA between the biological replicates was calculated and visualized using corrplot and pca3d packages of R (version 3.2.0; https://cran.r-project.org/) to estimate the relatedness between the biological replicates and the tissue samples. The normalized gene expression data was represented as FPKM (fragments per kilobase per million of reads mapped). All genes with an FPKM ≥ 1 were counted as expressed and considered for downstream analyses after removal of transposable element (TE)-related genes. Differential expression in the seed developmental stages was calculated by Cuffdiff program (v2.0.2) [180] with respect to flag leaf at *p*-value ≤ 0.05 and *q*-value ≤ 0.05. DEGs during seed development in SN and LGR were determined with respect to flag leaf (as vegetative control) from each genotype. Stringent cutoffs of FPKM value ≥ 1, log_2_fold change (log_2_fc) value ≥ 1 and *q*-value ≤ 0.05 were used for identification of DEGs. GO enrichment analysis of the expressed and DEGs was performed using agriGO software (v1.2) [181] and BiNGO plugin [182] of Cytoscape (version 3.4.0). Heatmaps and *k*-means cluster diagrams were prepared using MeV_4_6_0 [183] employing Euclidean distance method. Pathway annotations were performed using MapMan software (version 3.6.0RC1) [184]. Functional annotation of genes was performed using RGAP version 7 and funRiceGenes (https://funricegenes.github.io/). Bubble plots were prepared in R software using ggplot2 (Wickham, 2016) and reshape2 [185] packages.

### Real-time PCR validation

cDNA for qPCR validation were prepared from the RNA isolated from the five stages of seed development and flag leaf in two biological replicates as detailed previously, using Superscript™ III first-strand cDNA synthesis kit (Invitrogen™; USA) [37, 52, 53, 80, 177]. Primers for real-time PCR were made from the unique regions of the selected genes using Primer Express 3.0. miRNA sequence was taken from miRBase (http://mirbase.org). The assay was carried out with Fast SYBR® Green Master Mix (Applied Biosystems; USA) as mentioned previously [177]. Real-time PCR was done on 7500 Fast Real-Time System (Applied Biosytsems; USA), and 7500 software v2.0.1 was used for data analysis. Housekeeping gene, *OsACT1*, was used as the endogenous control for real-time PCR. Fold change was calculated by ΔΔCT method. List of primers has been given in Additional file 2: Table 17.

### QTL mapping

Molecular mapping of QTLs was performed to establish the correspondence amongst grain size/weight QTLs with the genes having pronounced expression especially during seed developmental stages in rice. For this, SNPs exhibiting differentiation between high (40 g) and low (10 g) 1000-grain weight parental genotypes of a F_5_ mapping population (LGR × Sonasal) were genotyped using the genomic DNA of 286 mapping individuals using the Illumina Infinium assay (https://www.illumina.com). The SNP genotyping information showing goodness-of-fit towards the expected Mendelian 1:1 segregation ratio was analyzed using the JoinMap 4.1 (http://www.kyazma.nl/index.php/mc.JoinMap) at a higher LOD threshold (3.0) with Kosambi mapping function. SNPs were mapped into defined linkage groups/chromosomes according to their centiMorgan (cM) genetic distances and physical positions (bp). The individuals along with parents of a mapping population were phenotyped for 2 years, for grain size/weight grown in the field at NIPGR, New Delhi. To identify grain size/weight QTLs, the genotyping data of SNPs mapped on 12 rice chromosomes was correlated with grain size/weight trait phenotypic data of mapping individuals and parental genotypes using composite interval mapping function of MapQTL 6 at a LOD threshold score > 2.5 with 1000 permutations (*p* < 0.05 significance). The phenotypic variation explained by each major grain size/weight QTL at a significant LOD was estimated. Further, the genes showing high differential expression in SN and LGR in each analysis were delineated. The physical positions of these genes were matched with the QTL genomic regions. The ones overlapping with a QTL were separated.

### Small RNA library preparation

Total RNA was isolated from the five seed developmental stages and flag leaf tissue of SN and LGR. cDNA library was prepared according to the TrueSeq™ Small RNA library preparation kit (Illumina®; USA). Briefly, adapters were ligated sequentially at 3′ and 5′ end of the RNA, respectively. cDNA was prepared to selectively enrich the adapter ligated RNA fragments by performing PCR with two primers that anneal to the ends of the adapters. Next, the cDNA prepared was amplified by PCR and indexed with RNA PCR primer 1 (RP1) and RNA PCR primer Index (RPIX), respectively. The cDNA libraries were gel purified, and the quality of the libraries was checked by running 1 μl of the products on Agilent tape station with DNA HS Screen tape. The obtained libraries were then normalized to 2 nM concentration for cluster generation on Illumina sequencing platforms by adding Tris HCL 10 mM, pH 8.5. Following this, single-end sequencing was performed using Illumina MiSeq® system.

### Small RNA sequencing and data analysis

Low-quality reads were trimmed using Cutadapt (v1.3) (Martin, 2011). The adapter removed reads were aligned against siRNA, snRNA, snoRNA, tRNA, and rRNA using nc-RNA databases (siRNAdb; http://sirna.sbc.su.se/sirnadb_050915.txt; NCBI Genbank; http://www.ncbi.nlm.nih.gov/genbank/; deepBase; http://deepbase.sysu.edu.cn/download.php; GtRNAdb; http://gtrnadb.ucsc.edu/; Rfam; http://rfam.xfam.org/) with Bowtie2 program v2.1.0 [186]. The unaligned clean reads of 17–35 bp length were used for miRNA identification by aligning to mature miRNAs of *Oryza sativa* in miRBase release-21 (http://www.mirbase.org/) using Bowtie program (version 0.12.9). The expression data of miRNAs were normalized using the TPM (transcripts per million) method. The differential expression analysis of the miRNAs was estimated using a negative binomial method with the DESeq package [187] of R (https://cran.r-project.org/) with default parameters. The targets of the known miRNAs were identified using psRNA Target [188] tool with default parameters.

### Stem-loop qRT-PCR

Stem-loop qRT-PCR was done to detect the levels of miR530-5p in SN and LGR seed developmental stages (S1–S5). Total RNA was isolated from S1–S5 stages and flag leaf of SN and LGR as mentioned above. The total RNA was purified using acid phenol (Ambion®; Naugatuck, CT, USA) as per manufacturer’s protocol. For stem-loop qRT-PCR, miR530-5p specific cDNA was synthesized using its specific stem-loop reverse transcription primer (miR530-5p SL primer) (Additional file 2: table 17). This cDNA was synthesized from 200 ng of total RNA by using Superscript™ III first-strand cDNA synthesis kit (Invitrogen™; USA) according to the manufacturer’s protocol. This cDNA was used for qRT-PCR assay using miR530-5p forward primer and a universal reverse primer. The conditions for qRT-PCR were maintained same as mentioned previously. snRNA U6 was used as an internal control. Three biological replicates were used for the assay and significance was calculated using Student’s *t* test with *p* value˂0.001 denoted as double asterisk (**).

### Total starch and protein isolation and quantification

A total of 100 mg of developing rice seeds from each of S1–S5 stages of SN and LGR were finely ground to powder in liquid nitrogen and incubated for 10 min at 4 °C after homogenizing with 1 mL of lysis buffer (20 mM Tris pH 7.6, 150 mM NaCl, 1 mM EDTA, 1 mM DTT, 1 mM PMSF). The insoluble debris was removed from the mixture by spinning at 100 g for 10 min at 4 °C. Supernatant was centrifuged at 12,000* g* for 10 min at 4 °C to obtain the soluble protein fraction [189]. Total isolated proteins from each sample were quantified according to the Bradford method [190] (Amresco M173-KIT).

Seeds from all developmental stages (S1–S5) of SN and LGR were crushed using liquid nitrogen. One hundred milligrams of the crushed sample was used for isolating starch. Starch isolation and determination was done by using starch assay kit (Megazyme, Wicklow, Ireland, http://www.megazyme.com/), according to the manufacturer’s protocol. Starch estimation was done in two independent biological replicates.

### Database development

RGDD has been developed using Tableau Software (https://www.tableau.com/), which is a leading data visualization software used for reporting and analyzing vast volumes of data. The background data input for this database was in CSV format. It was obtained subsequent to the abovementioned transcriptome and miRNome analyses. The programming was done within Tableau Software itself.

## Supplementary Information

**Additional file 1: Figure S1.** (related to Fig. 1). Morphological changes in seeds of SN and LGR during development. (A) SEM images of husk of SN (left) and LGR (right) showing the middle portion of lemma. Individual cells have been marked by asterisks (scale bar = 100μm). (B) Images of seeds of SN and LGR. S1–S5 represent five stages of rice seed development. Left and right panels contain seeds (husked and dehusked) of SN and LGR from representative DAP of the five seed stages. (C) An additional set of endosperm sections of SN (left) and LGR (right) at DAP mentioned on the left side, representing S2, S3 and S4 stages of seed development stained with toluidine blue-O. i, v, ix, xiii, xvii and iii, vii, xi, xv, xix show central endosperm of SN and LGR, respectively, while ii, vi, x, xiv, xviii and iv, viii, xii, xvi, xx show peripheral endosperms of SN and LGR, respectively. Red and blue triangles indicate nuclei and cell wall, respectively. Scale bar = 50 µm. **Figure S2.** Correlation between biological replicates of SN and LGR. (A) Validation of RNA sequencing data by qPCR of five genes in SN and LGR. In each box (gene names are mentioned at the top), the upper panel indicates SN and lower panel indicates LGR. The light brown and light green bars represent relative expression (log2fold change) values from RNA sequencing (*n*=3; q value≤0.05) and the dark brown and dark green bars represent relative qPCR expression values (*n*=2; error bars represent ± SD), respectively. S1–S5 represent five stages of seed development and Leaf represents flag leaf. See replicate data in Additional file 2: Table S18. (B) Pearson’s correlation between the three biological replicates (_1, _2, _3) from five seed stages (S1–S5) and flag leaf (Leaf) of SN (top) and LGR (bottom). Color legend represents the value of Pearson’s correlation coefficient ranging from -1 to +1. Blue color represents high correlation. (C) PCA plot representing grouping patterns of biological replicates. The plot depicts the relatedness between the biological replicates of a sample as well as between the seed stages and leaf tissue in terms of their transcriptome. The color coding used for denoting the samples in the plot has been shown in the color legend. **Figure S3.** Expression profiles of SN and LGR. The Venn diagram represents the total number of genes expressed specifically in seeds of SN and LGR, i.e., these genes are not expressed (FPKM<1) in the flag leaf. GO enrichment analysis of seed-specific genes common between SN and LGR (as marked in red in the figure) was performed using BINGO plug-in of Cytoscape. Purple and violet colors indicate enriched GO categories at p values mentioned in the color legend. White color indicates no significant enrichment. Highly enriched GO terms have been encircled. Interaction between seed-specific GO terms has been shown by means of edges (arrows) in the network diagram. Nodes = GO terms, edges = interactions. **Figure S4.** Analysis of differential expression profiles of seeds of SN and LGR. (A) Total number of differentially expressed genes obtained in seed developmental stages of SN and LGR. The genes were filtered at the cutoffs of FPKM≥1, log2fc≥1 and q value≤0.05. Violet and green bars indicate SN and LGR, respectively. (B) Genes with log2fc≥10 in SN and LGR were annotated using MapMan (violet and green bars indicate SN and LGR, respectively as indicated in the color legend; bars on the right and left sides represent up- and downregulated genes, respectively). Pathways with ≥50 genes upregulated in both SN and LGR have been marked with red arrows. Pathways with significant number of genes differentially expressed only in SN and LGR have been marked with asterisks (blue = SN, pink = LGR). (C) Transcription factor families with ≥5 upregulated members with log2fc≥10 in both SN and LGR have been plotted (solid blue bars represent SN and bars with pattern filling represent LGR as shown in the color legend). **Figure S5.** Comparison of stage-specific DEGs. Stage-specific DEGs (log2fc≥1 in one stage and log2fc<1 in rest of the four stages in a genotype) were compared to study the similarity in the transcriptomes of SN and LGR. Bars above and below the X-axis represent up and down regulation, respectively, as indicated. **Figure S6.** (related to Fig. 3). Differential expression analysis of genes related to cell cycle in SN and LGR. (A) GO enrichment analysis of all upregulated genes of SN and LGR, respectively, was performed by AgriGO. Yellow, orange and red colors indicate significant enrichment as denoted in the color legend. The relationship between GO terms is as indicated in the color legend by arrows. GO terms enriched specifically and enriched at higher p-value in LGR have been demarcated with red and blue dotted boxes, respectively. (B) The distribution of the cell cycle genes specifically upregulated in SN (denoted by the black box) was studied in the seed developmental stages of SN (as represented in the color legend). S5 stage of SN had the maximum number of cell cycle related DEGs (marked by asterisks in the graph). (C) FPKM values of genes, which promote endoreduplication, and are commonly upregulated in SN and LGR but exhibit different expression patterns (such as variation in peak expression) between the two genotypes. These included genes encoding Wee1 kinases and mitotic spindle checkpoint protein MAD2, which inhibit anaphase progression. **Figure S7.** (related to Fig. 3). Functional annotation of LGR S3-preferential DEGs. (A) Per cent similarity between DEGs of consecutive stages of SN (bars above and below the axis represent up- and downregulated genes, respectively). (B) Pathway analysis of DEGs (by MapMan) that are LGR S3 preferential, i.e up regulated in LGR S3 but not in LGR S2 and SN S2. Genes related to degradation of starch, TCA cycle, mitochondrial ETC and cell wall modification were upregulated (demarcated with pink boxes in the figure). **Figure S8.** (related to Fig. 4). Expression pattern of carbohydrate and PCD-related genes in seeds of SN and LGR. (A) A phosphofructokinase and an α-glucan phosphorylase gene showed high expression levels (log2FPKM≥5) in the seed tissues of SN and LGR. Arrows indicate peak in expression values (log2FPKM). (B) Heatmap representing log2FPKM values of known genes related to PCD in seed and leaf tissues of SN and LGR (color legend at the bottom). The positive and negative regulators of PCD showing difference in expression levels and pattern between SN and LGR have been marked in red and green boxes, respectively. (C) Graph showing log2FPKM values of four positive and one negative regulator of PCD, marked in (B). Positive and negative regulators have been denoted by round and square markers, respectively. **Figure S9.** (related to Fig. 6). Categorization of differentially expressed TFs based on their regulation patterns amongst SN and LGR. TFs that were DE (815 and 545 upregulated while 545 and 480 downregulated in SN and LGR, respectively) in SN and LGR seeds were divided into four categories [up in any stage in both SN and LGR (473 TFs; from 59 families), down in any stage in both SN and LGR (340 TFs; from 54 families), up in any stage in SN and down in LGR (102 TFs; 30 families), up in any stage in LGR and down in SN (28 TFs; 18 families); pie chart in the center] based on their regulation. Left and right panels indicate TF families with similar and opposite regulation pattern, respectively. Prominent TF families (with ≥20 members) showing similar regulation pattern in SN and LGR have been marked with asterisks in the color legend (number of members has been written on the pie charts). **Figure S10.** (related to Fig. 7). Expression analysis of hormone signaling genes with different expression patterns in SN and LGR. (A-H) Heat maps showing expression levels (log2FPKM) of genes related to auxin, ABA, GA, ethylene, BR, CK, JA and SA signaling, respectively, that showed different expression pattern between SN and LGR, and hence, were present in separate co-expression clusters. In each heat map, the upper and lower panels show expression levels in five stages of seed development (S1–S5) and flag leaf (Leaf) in SN and LGR, respectively, as mentioned along with the locus ID of the gene. Red dots indicate genes that have higher expression (log2FPKM≥0.5) in any stage in either genotype, and have been plotted in Fig. 7. SY = synthesis, DG = degradation, SG = signal transduction, RS = response. **Figure S11.** Expression analysis of genes reported for regulating seed size and weight in rice. (A, B) Regulation pattern of genes reported in literature in seed developmental stages of SN and LGR as mentioned in the figure. (C) Heat map based on log2FPKM values and patterns of genes regulating seed size and weight in the seed developmental stages (S1–S5) and leaf tissues of SN and LGR. Red dot = negative regulator of seed size and weight, green dot = positive regulator of seed size and weight, blue dot = affects both seed length and width. The arrows indicate comparison of expression patterns and levels between SN and LGR. Genes with similar expression pattern between SN and LGR have been marked with an orange or black arrow. In this, dark orange arrow marks genes with higher expression level (Log2FPKM≥1) in SN while light orange arrow marks the same genes with lower expression in LGR. Gray arrow marks genes with higher expression (Log2FPKM≥1) in LGR while black arrow marks the same genes with lower expression level in SN. Since these genes have similar expression pattern, the homologs from SN and LGR mostly appear close to each other in the hierarchy. Green arrows mark genes with different expression pattern between SN and LGR, with dark and light green indicating SN and LGR, respectively. Blue box is for genes which do not express in both SN and LGR (as indicated by color legend on top). (D) Graphs representing expression (FPKM) of genes known to regulate seed size and weight in rice in the five seed stages of SN and LGR. Graphs a-h show genes with different expression patterns between SN and LGR; graphs i-m show genes with similar expression patterns but different expression levels amongst SN and LGR. S1–S5 represent five seed stages, locus IDs and gene names have been mentioned above each graph, brown and green lines represent SN and LGR, respectively. **Figure S12.** Expression profiles of miRNAs in the seeds of SN and LGR. (A) Validation of sRNAseq data by stem-loop qRT-PCR of miR530-5pin SN (left panel) and LGR (right panel) (n=3). The lower panel represents log2 fold change by sRNAseq (See replicate data in Additional file 2: Table S18). (B) Total number of miRNAs expressed (TPM≥50) in seed developmental stages of SN and LGR (ovals on top) and corresponding number of families (ovals at bottom). The number of families specific (miRNAs with TPM≥50 in one genotype and TPM<50 in the other) to and common (TPM≥50 in both genotypes) between SN and LGR have been written in black and red, respectively. Families with miRNAs expressed specifically in SN and LGR have been mentioned below the Venn diagram. (C) Number of miRNAs that are specific to (miRNAs with TPM≥50 in one stage and TPM<50 in the other stages) and common (TPM≥50 in all five stages) between seed developmental stages (S1–S5) of SN and LGR. Green and orange bars represent specific miRNAs in SN and LGR, respectively (as indicated in the color legend). Blue ovals represent miRNAs common between SN and LGR. **Figure S13.** Differential expression profile of miRNAs in the seeds of SN and LGR. (A) Total number of differentially expressed miRNAs (DEMs, log2fc≥2) obtained in seed developmental stages of SN and LGR with respect to flag leaf. Blue bars represent SN and orange bars represent LGR (as indicated in the color legend). (B) Comparison of DEMs between same stages/SS and comparable stages/CS of SN and LGR. Numbers of DEMs for SS are in blue while for CS are in brown. **Figure S14.** miRNAs commonly differentially expressed in all five stages of SN or LGR and their target mRNAs. Graphs show differential expression levels (log2fc) of miRNAs (A) up- and (B) downregulated in all five stages of each genotype. The targets have negative correlation in expression and are also down or upregulated, respectively, in all stages of the genotype. For both A and B, upper panel is for SN while lower one is for LGR. In A, bars above and below the X axis represent miRNAs up- and their targets downregulated, respectively. In B, bars above and below the X axis represent miRNAs down- and their targets upregulated, respectively. The horizontal trendlines in each graph depict negative correlation amongst differential expression of miRNAs and their targets. **Figure S15.** Functional overlay of whole transcriptome data. Functional annotation of genes derived from seven analyses (conditions) of whole transcriptome sequencing to find out genes involved in/coding for TFs, hormone signaling, cell cycle, cell growth, SSPs and carbohydrate metabolism with the overall analyses. The description of the analyses is given in the first column. Description of the genotype and stage is given in the next column. The total numbers of genes present in that particular category, which were used for subsequent functional annotation, have been mentioned in parenthesis. For each analysis, the numbers of genes falling in different functional categories have been mention in respective rows. **Figure S16.** Screenshot of Rice Grain Development Database (RGDD). (A) The transcriptome tab allows search either by locus ID (option 1) or by function (option 2). The functions enlisted are transcription factors and phytohormones (related genes). Transposable elements can also be selected for. A gene list is displayed for option 2, from which locus IDs can be selected. Both expression and log2 fold change data are shown as graphs. (B) The miRNome tab allows for search as miRNA ID (option 1) or from a list of expressed miRNAs (option 2). Both expression and log2 fold change data are displayed as line graphs.

**Additional file 2: Table S1.** Summary of reads obtained from total RNA sequencing of 36 cDNA libraries from the five seed developmental stages (S1-S5) and flag leaf (L) of SN and LGR. **Table S2.** Pearson’s correlation between biological replicates of the five seed developmental stages (S1-S5) and flag leaf (L) of SN and LGR. **Table S3.** FPKM values of all rice genes (transposable elements removed) in flag leaf and five seed development tissues of SN and LGR. The higlighted ones indicate significant DEGs in at least one seed stage. **Table S4.** Log2fold change values in seed development stages, of significant DEGs (Log2FC≥1, q value≤0.05, p value ≤0.05, FPKM≥1). Non-significant differential expression is indicated by ‘-’. **Table S5.** Stage-specific DEGs common between SN and LGR. Log2 fold change values of DEGs which are specific (log2FC≥1 in that stage and <1 in rest) to the same stage of SN and LGR. **Table S6.** FPKM values of cell cycle related genes specifically up regulated in seeds of SN and LGR. **Table S7.** List of DEGs preferential to S3 stage of LGR by comparison with SN S3/LGR S2 and SN S2/LGR S2. Genes which are LGR S3 preferential in both analyses have been highlighted. **Table S8.** List of TF encoding genes (with their log2fold changes), significantly differentially expressed at log2fc≥1 in at least one seed developmental stage of SN and/or LGR. “-” indicates non-significant values. **Table S9.** FPKM values of phytohormone encoding genes, which were present in different expression clusters (variation in expression pattern) and had higher expression values (difference in log2FPKM≥0.5; variation in expression level) in a stage in either SN or LGR. **Table S10.** Summary of grain size QTLs identified using a mapping population (Sonasal x LGR) in rice. **Table S11.** Log2 fold change values of genes which are significantly differentially expressed amongst SN and LGR seed development stages and are located within QTLs associated with a seed size-related trait. **Table S12a.** Summary of reads obtained from small RNA sequencing of each library from the five seed developmental stages (S1-S5) and flag leaf (L) of SN. In all samples, _1, _2, _3 represent biological replicates. **Table S12b.** Summary of reads obtained from small RNA sequencing of each library from the five seed developmental stages (S1-S5) and flag leaf (L) of LGR. In all samples, _1, _2, _3 represent biological replicates. **Table S13.** Pearson's correlation between biological replicates of the five seed developmental stages (S1-S5) and flag leaf (L) of SN and LGR obtained from small RNA sequencing. **Table S14.** Expression of all miRs in seed and flag leaf of SN and LGR. The TPM values of 604 miRs detected in at least one of the tissues used has been mentioned. The TPM values ≥50 have been highlighted in gray. **Table S15.** Comparison of DEMs between SN and LGR. The orange colored cells indicate the log2 fold changes of 467 DEMs for each stage of seed development. The yellow color indicates comparison between similar stages (by developmental event) of SN and LGR. The grey color indicates comparison between same stage (by DAP) of SN and LGR. U implies 'up regulation' while D implies 'down regulation' in the respective stage mentioned. - indicates non-significant differential expression. The up regulated genes have been marked in green while down regulated ones are in red. **Table S16.** List of miRNA-target modules for miRNAs up and down-regulated in all five seed developmental stages of either SN or LGR. **Table S17.** List of primers for validation. **Table S18.** Details of replicate data.

## Data Availability

All data generated or analyzed during this study are included in this published article, its additional files and publicly available repositories. High-throughput RNAseq data has been deposited in the Gene Expression Omnibus (GEO) database under the SRA accession number PRJNA605919, ID 605,919—BioProject—NCBI (nih.gov). The small RNAseq data has been deposited under SRA accession number PRJNA616068, ID 616,068—BioProject—NCBI (nih.gov). The analyzed data can be accessed at the Rice Grain Development Database at www.nipgr.ac.in/RGDD/index.php.

## Abbreviations

ABA: Abscisic acid
AGPs: Arabinogalactan proteins
BR: Brassinosteroid
CBB: Coomassie Brilliant Blue
CK: Cytokinin
cM: Centi Morgan
CS: Comparable stages
DAP: Days after pollination
DEGs: Differentially expressed genes
DEMs: Differentially expressed miRNAs
ETC: Electron transport chain
FPKM: Fragments per kilobase per millions of reads mapped
GA: Gibberillic acid
GDSL: Gly-Asp-Ser-Leu
GO: Gene ontology
GS: Grain size
JA: Jasmonic acid
LEA: Late embryogenesis abundant proteins
LGR: Large grained rice
LOD: Logarithm of the odds
PCA: Principal component analysis
PCD: Programmed cell death
PHD: Plant homeo domain
QTL: Quantitative trait locus
RGAP: Rice Genome Annotation Project
RGDD: Rice Grain Development Database
RP1: RNA PCR Primer1
RPIX: RNA PCR Primer Index
SA: Salicylic acid
SEM: Scanning electron microscopy
SN: Sonasal
SNPs: Single-nucleotide polymorphisms
SS: Same stages
SSP: Seed storage protein
TAG: Triacyl glycerol
TCA: Tricarboxylic acid
TE: Transposable element
TFs: Transcription factors
TPM: Transcripts per million
TT: Tai tobacco
UGT: UDP glucosyl and glucoronyl transferase
YT: Yan tobacco
