# Supplementary material for: Cytological, transcriptome and miRNome temporal landscapes decode enhancement of rice grain size
Source: BMC Biol. 2023 Apr 19;21:91. doi: 10.1186/s12915-023-01577-3 (PMC10116700; doi:10.1186/s12915-023-01577-3)
Supplement: Supplementary file 1 — Additional file 1: Figure S1. (related to Fig. 1). Morphological changes in seeds of SN and LGR during development. (A) SEM images of husk of SN (left) and LGR (right) showing the middle portion of lemma. Individual cells have been marked by asterisks (scale bar = 100μm). (B) Images of seeds of SN and LGR. S1–S5 represent five stages of rice seed development. Left and right panels contain seeds (husked and dehusked) of SN and LGR from representative DAP of the five seed stages. (C) An additional set of endosperm sections of SN (left) and LGR (right) at DAP mentioned on the left side, representing S2, S3 and S4 stages of seed development stained with toluidine blue-O. i, v, ix, xiii, xvii and iii, vii, xi, xv, xix show central endosperm of SN and LGR, respectively, while ii, vi, x, xiv, xviii and iv, viii, xii, xvi, xx show peripheral endosperms of SN and LGR, respectively. Red and blue triangles indicate nuclei and cell wall, respectively. Scale bar = 50 µm. Figure S2. Correlation between biological replicates of SN and LGR. (A) Validation of RNA sequencing data by qPCR of five genes in SN and LGR. In each box (gene names are mentioned at the top), the upper panel indicates SN and lower panel indicates LGR. The light brown and light green bars represent relative expression (log2fold change) values from RNA sequencing (n=3; q value≤0.05) and the dark brown and dark green bars represent relative qPCR expression values (n=2; error bars represent ± SD), respectively. S1–S5 represent five stages of seed development and Leaf represents flag leaf. See replicate data in Additional file 2: Table S18. (B) Pearson’s correlation between the three biological replicates (_1, _2, _3) from five seed stages (S1–S5) and flag leaf (Leaf) of SN (top) and LGR (bottom). Color legend represents the value of Pearson’s correlation coefficient ranging from -1 to +1. Blue color represents high correlation. (C) PCA plot representing grouping patterns of biological replicates. The plot depicts [file 12915_2023_1577_MOESM1_ESM.pptx]

## Slide 1
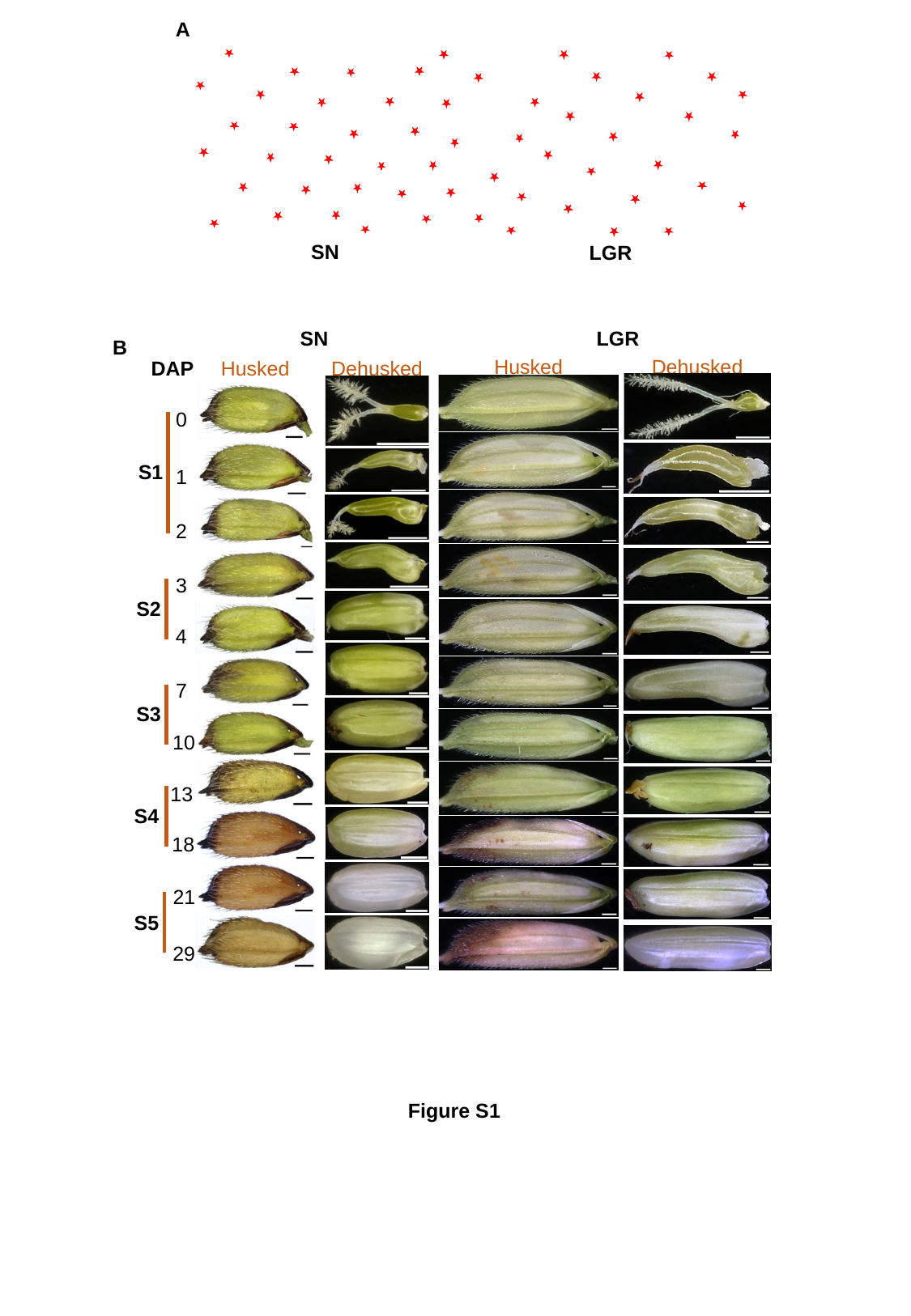

A
SN
LGR
SN
LGR
B
Husked
Dehusked
Husked
Dehusked
DAP
0
S1
1
2
3
S2
4
7
S3
10
13
S4
18
21
S5
b.
29
Figure S1

## Slide 2
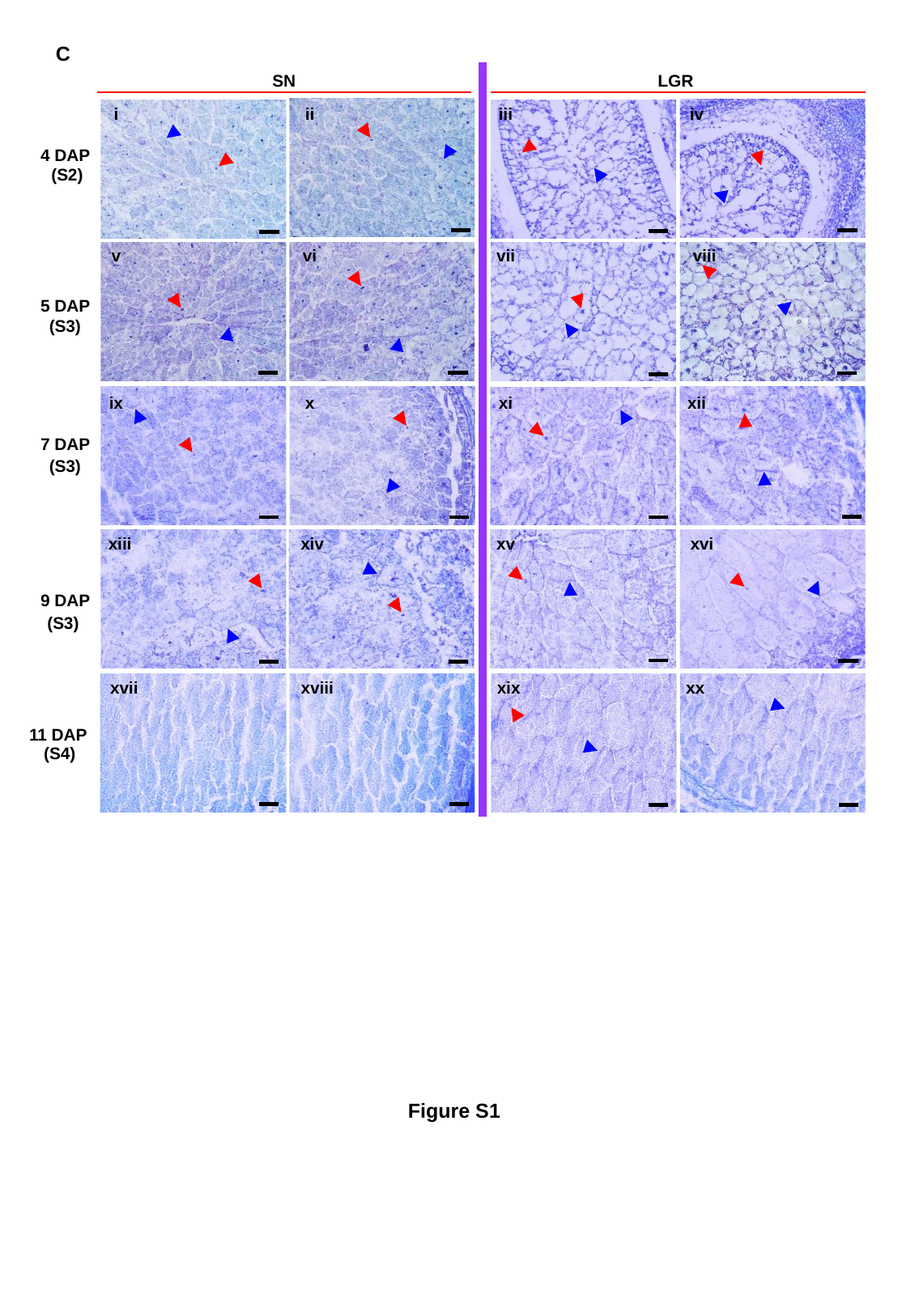

C
SN
LGR
i
ii
iii
iv
4 DAP
(S2)
v
vi
vii
viii
5 DAP
(S3)
ix
x
xi
xii
7 DAP
(S3)
xiii
xiv
xv
xvi
9 DAP
(S3)
xvii
xviii
xix
xx
11 DAP
(S4)
Figure S1

## Slide 3
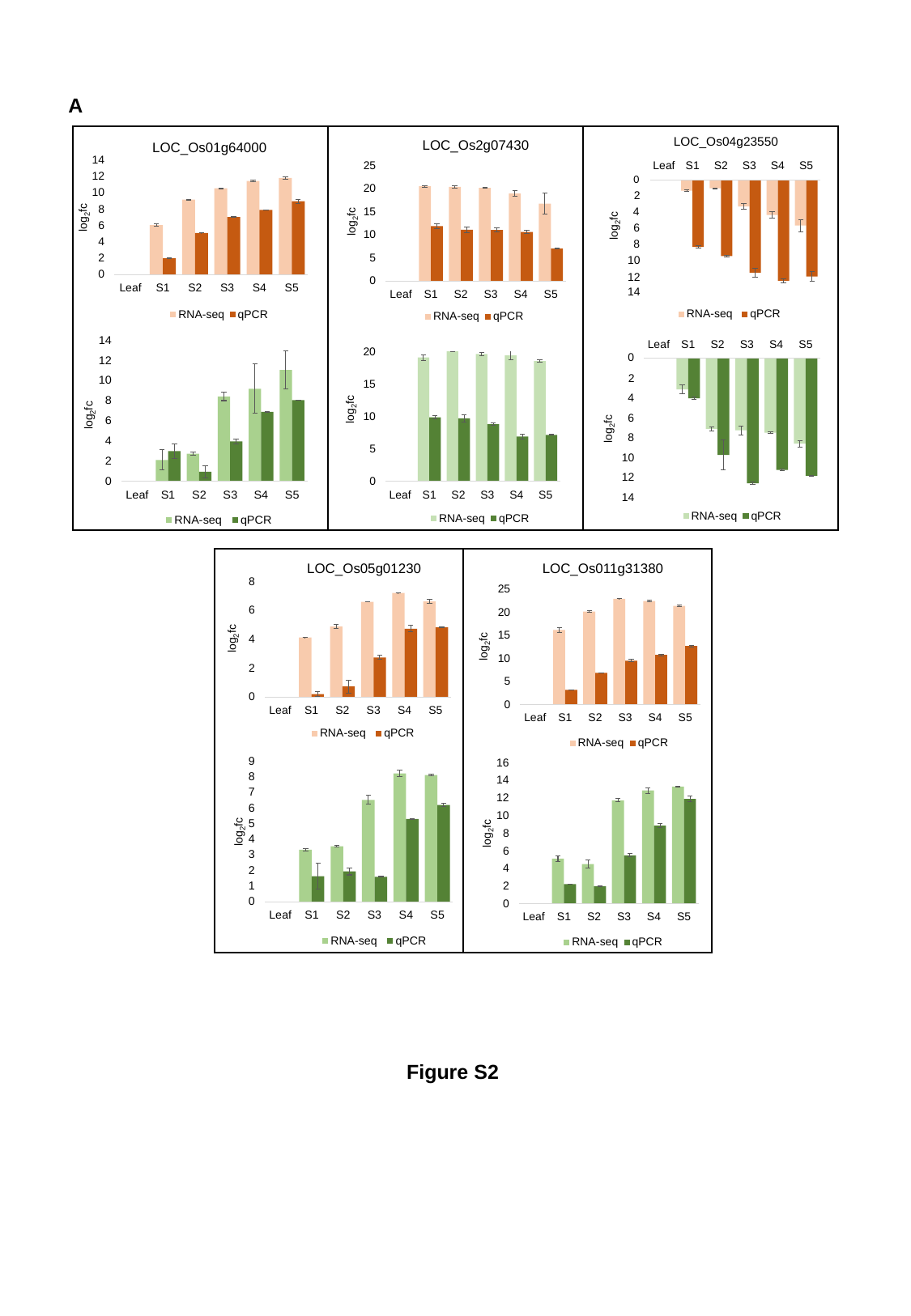

A
Figure S2

## Slide 4
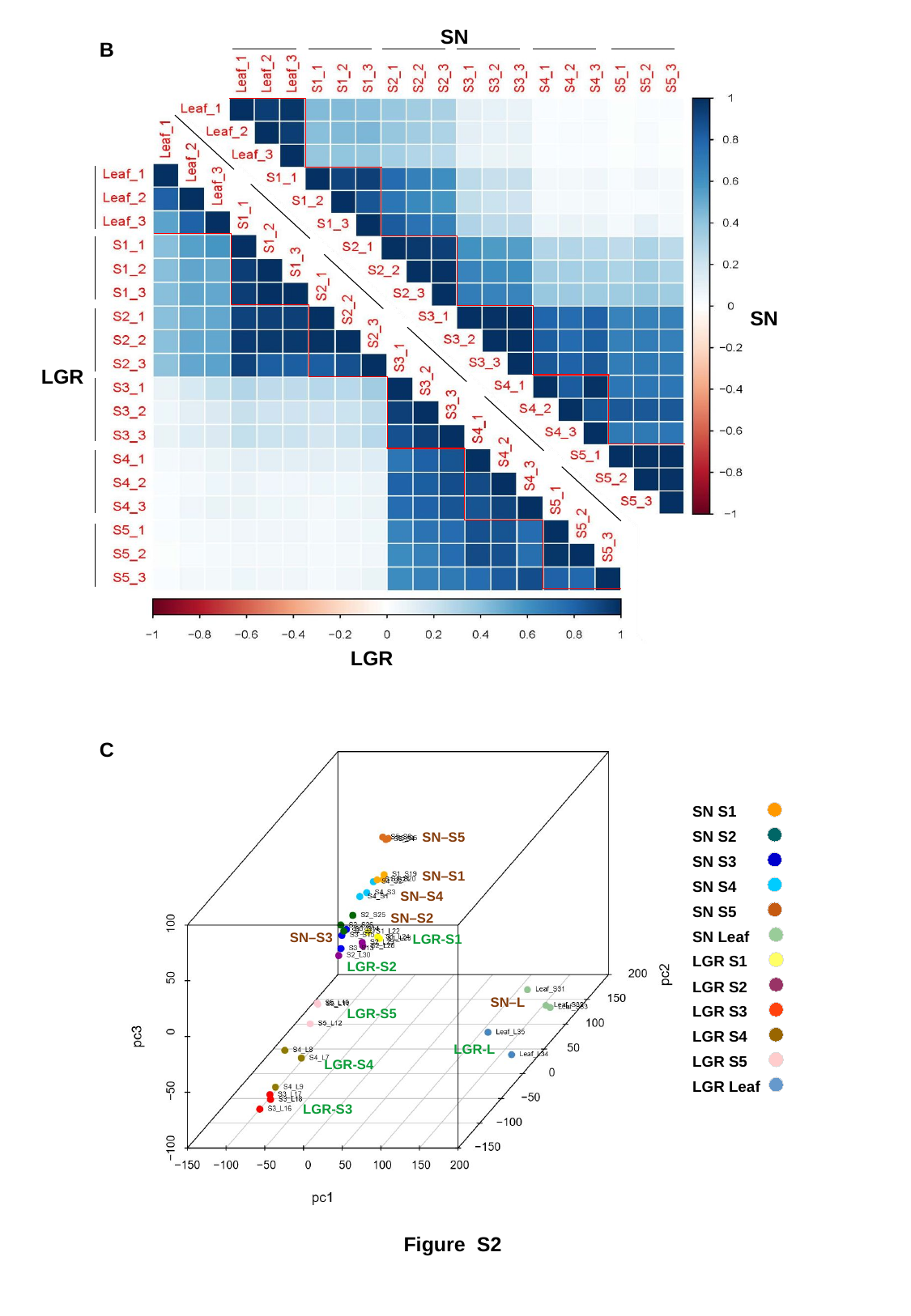

SN
B
SN
LGR
LGR
C
SN S1
SN S2
SN S3
SN S4
SN S5
SN Leaf
LGR S1
LGR S2
LGR S3
LGR S4
LGR S5
LGR Leaf
SN–S5
SN–S1
SN–S4
SN–S2
SN–S3
LGR-S1
LGR-S2
SN–L
LGR-S5
LGR-L
LGR-S4
LGR-S3
Figure S2

## Slide 5
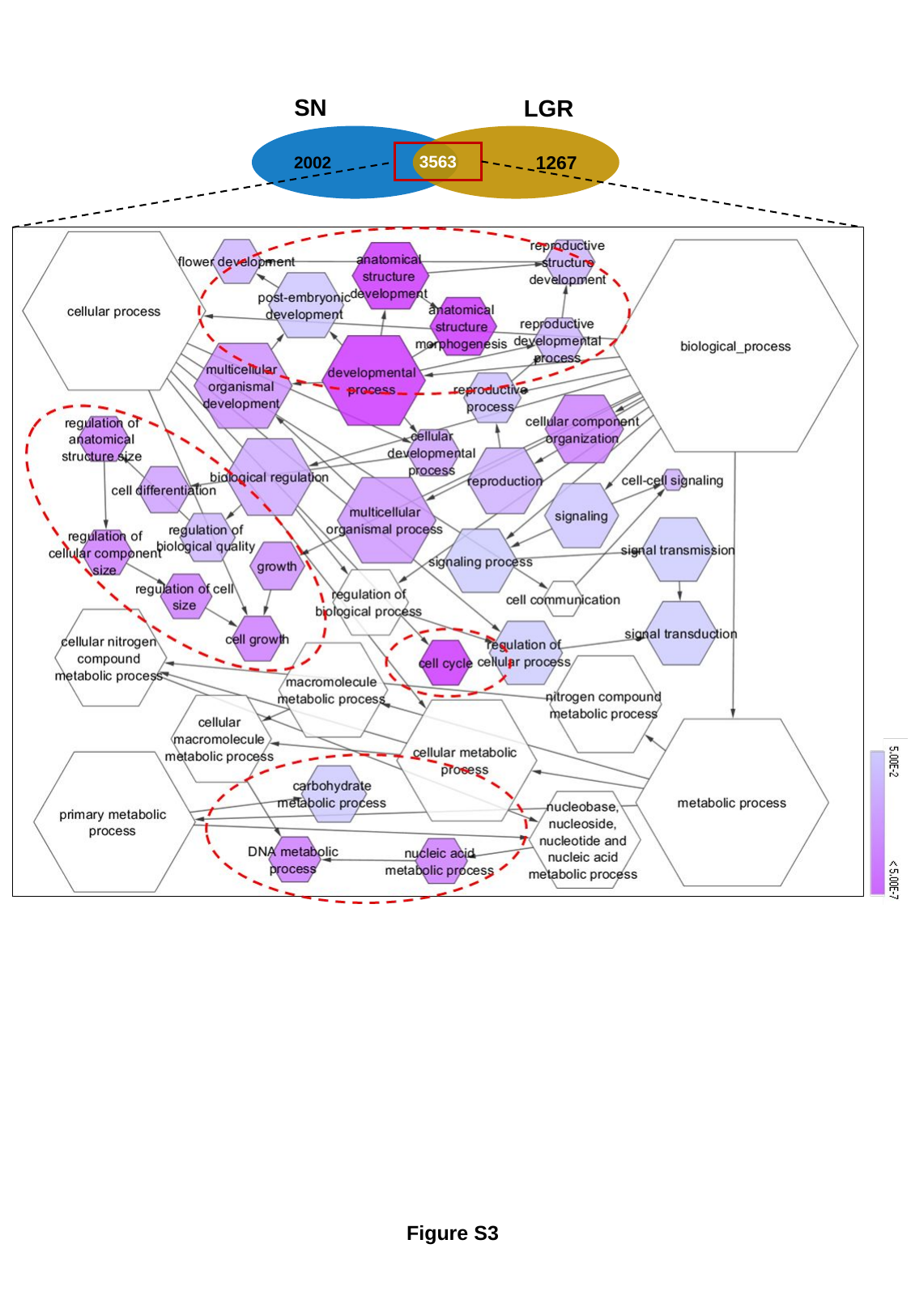

SN
LGR
2002
1267
3563
Figure S3

## Slide 6
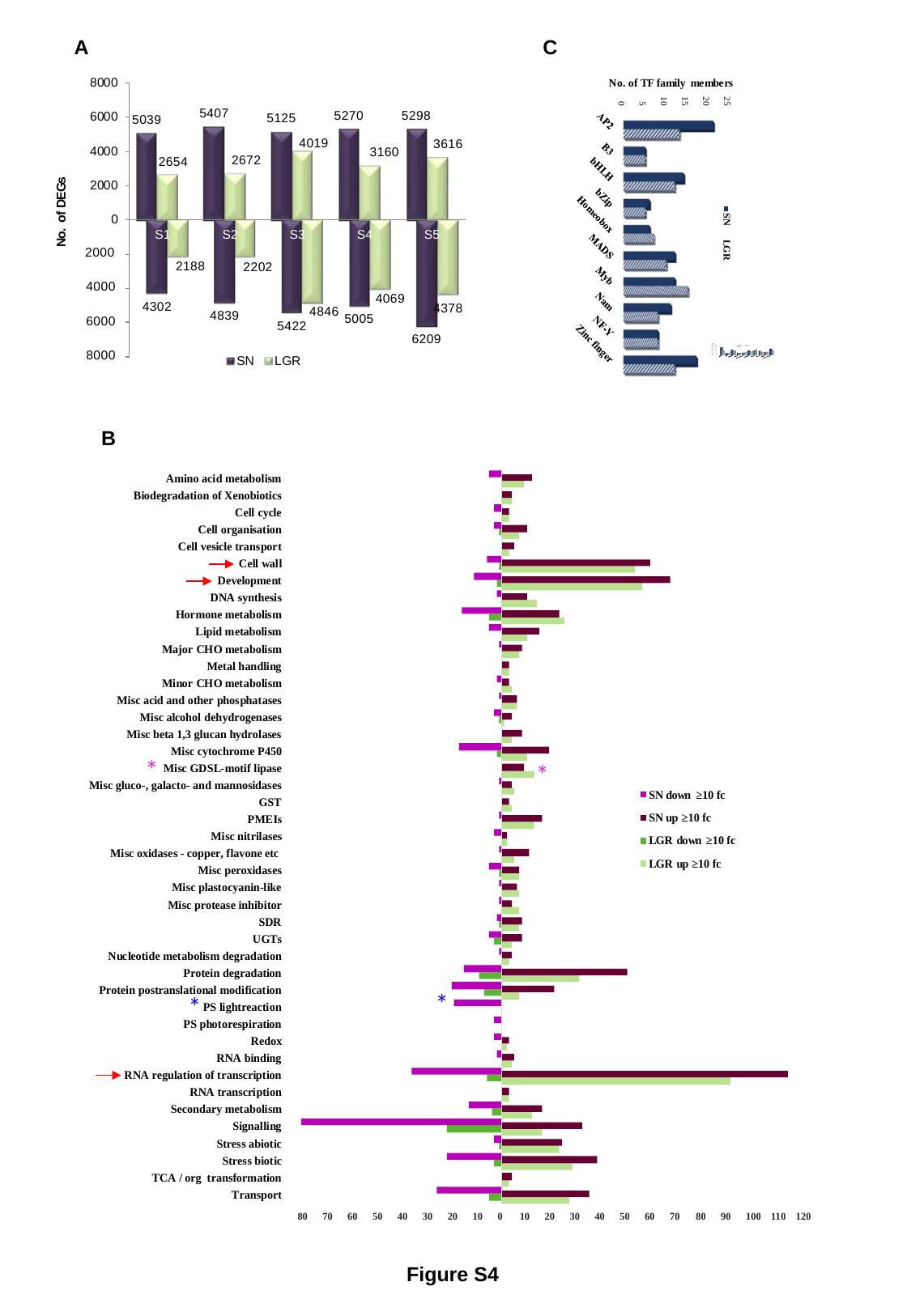

a
A
C
B
Figure S4

## Slide 7
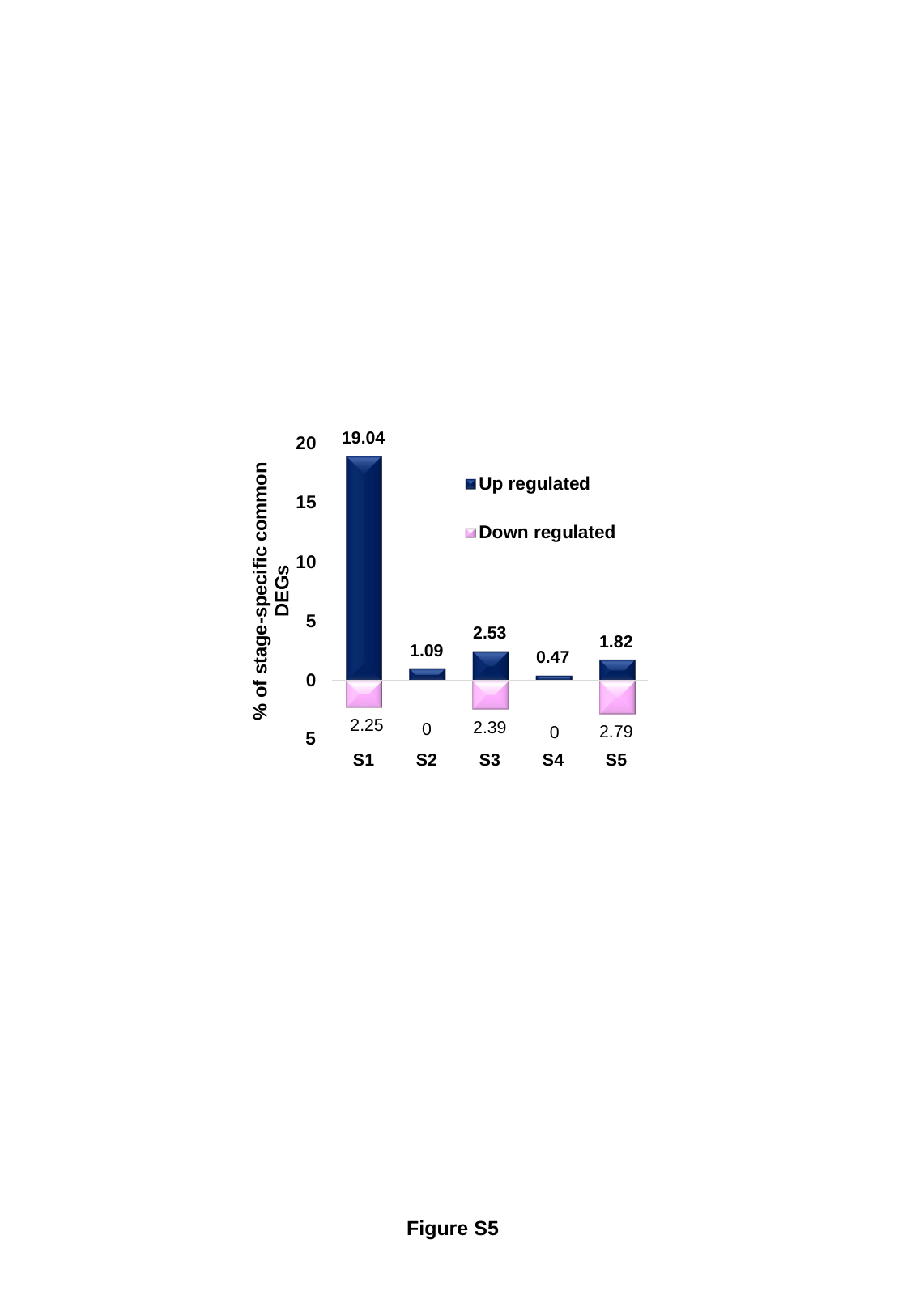

a
c
d
Figure S5

## Slide 8
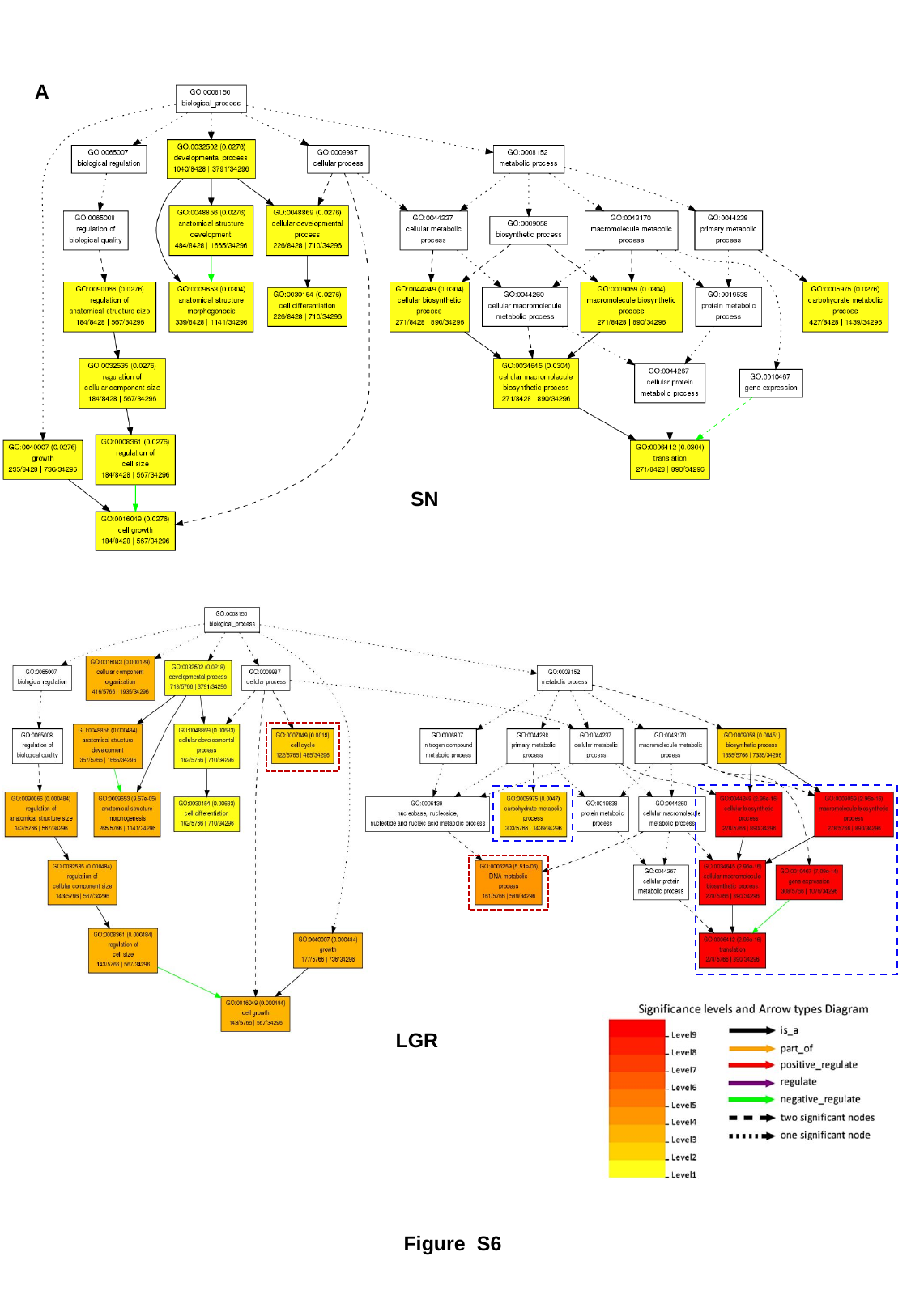

A
SN
LGR
Figure S6

## Slide 9
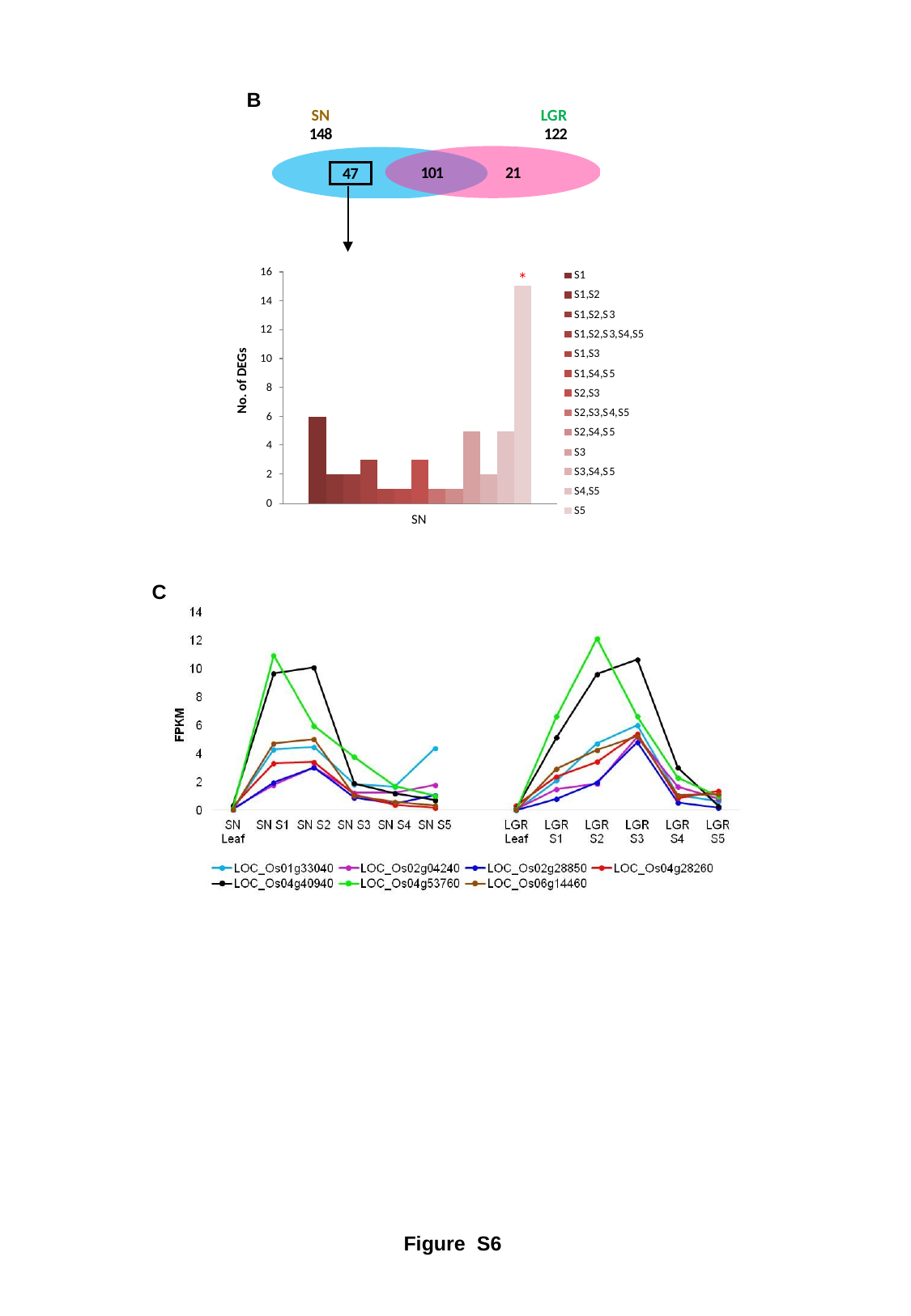

B
C
Figure S6

## Slide 10
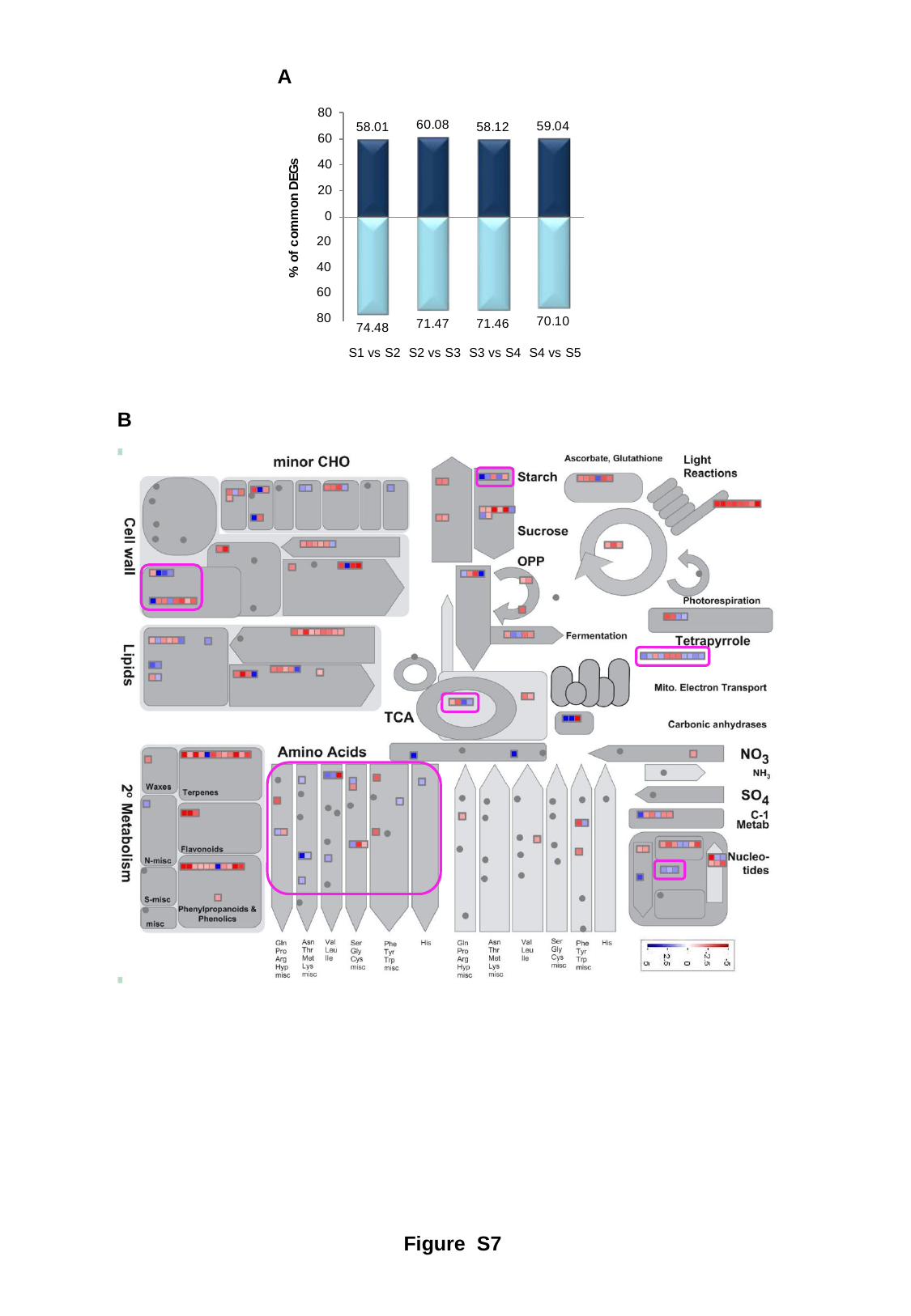

A
B
Figure S7

## Slide 11
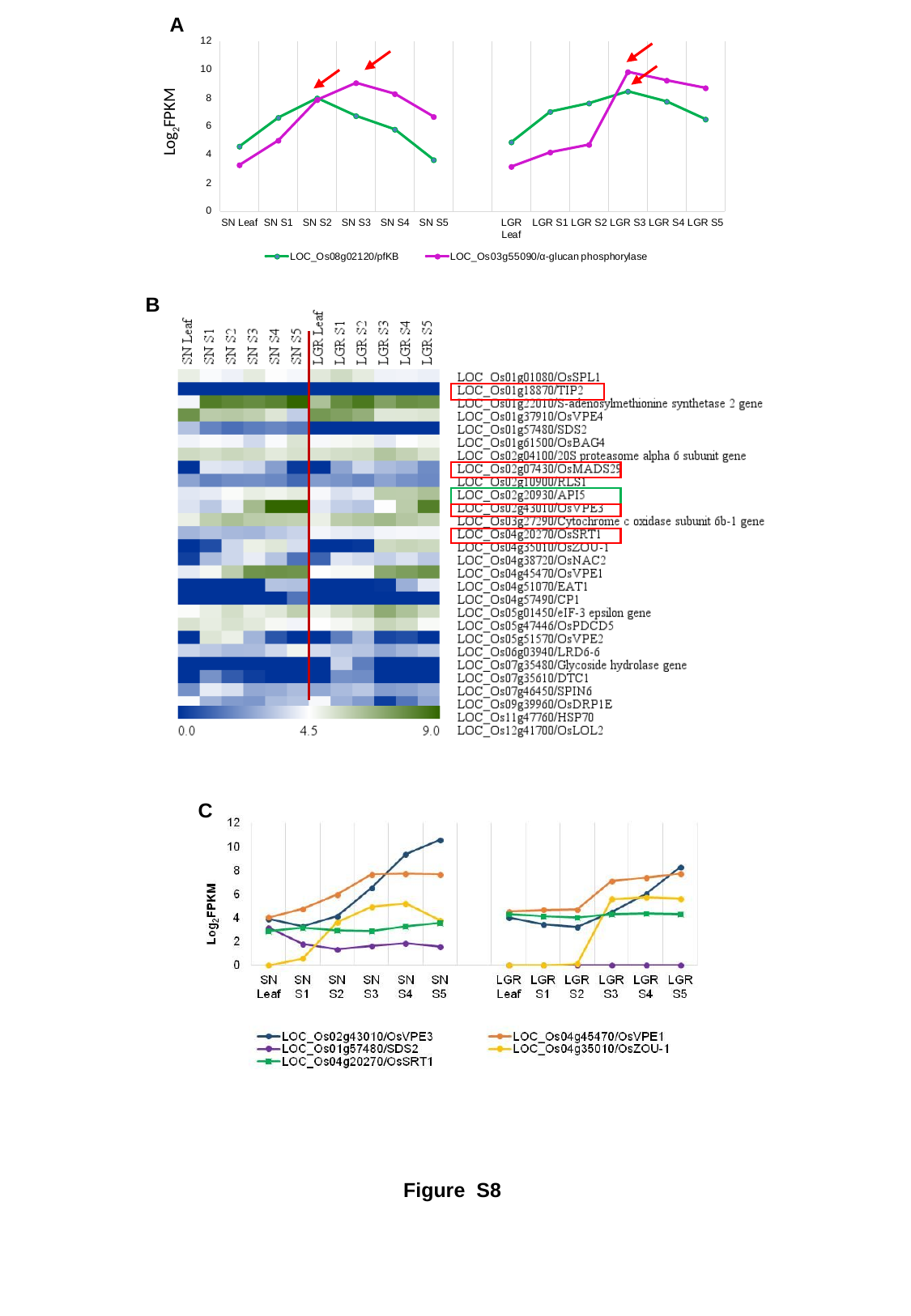

A
Log2FPKM
B
C
Figure S8

## Slide 12
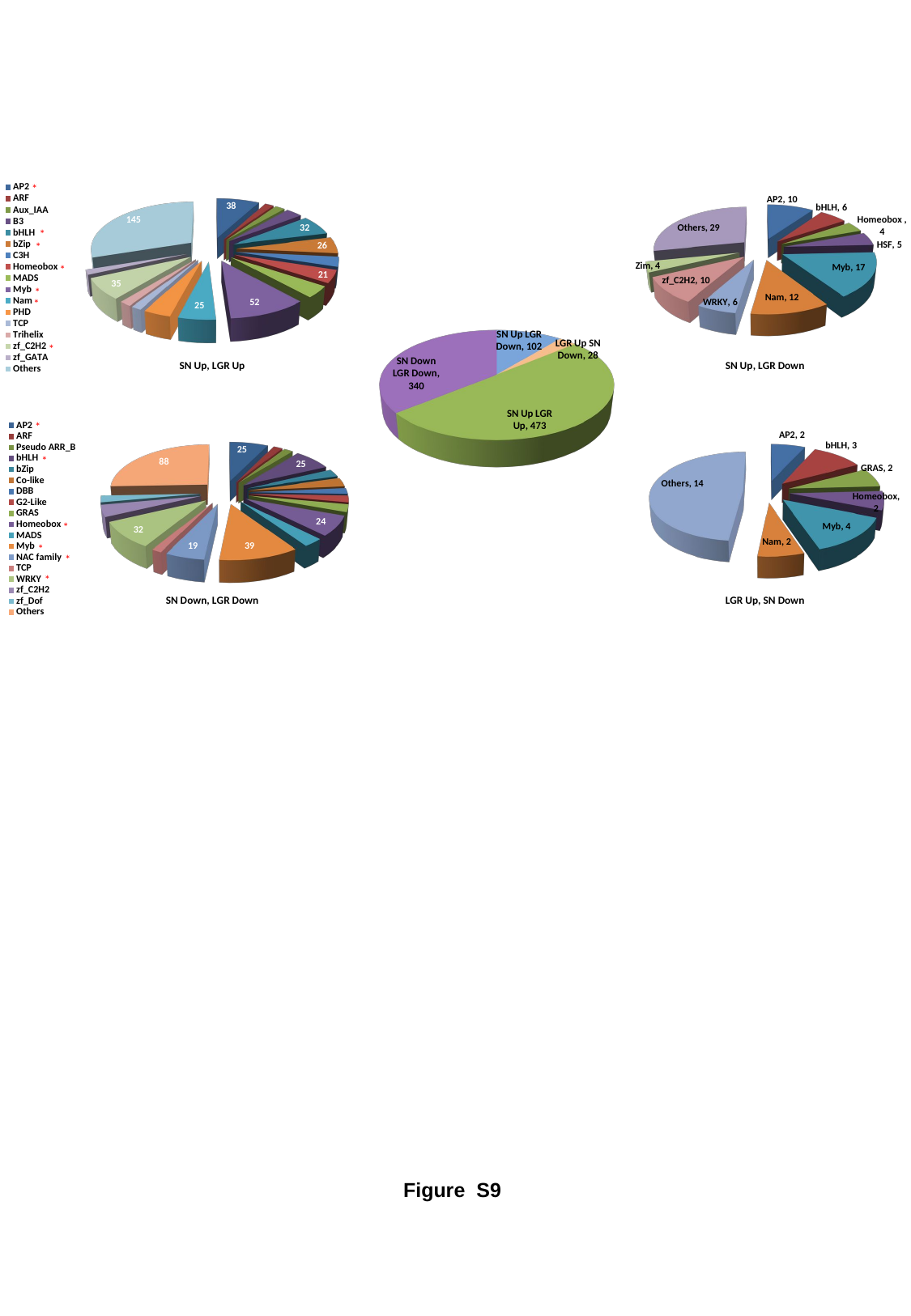

Figure S9

## Slide 13
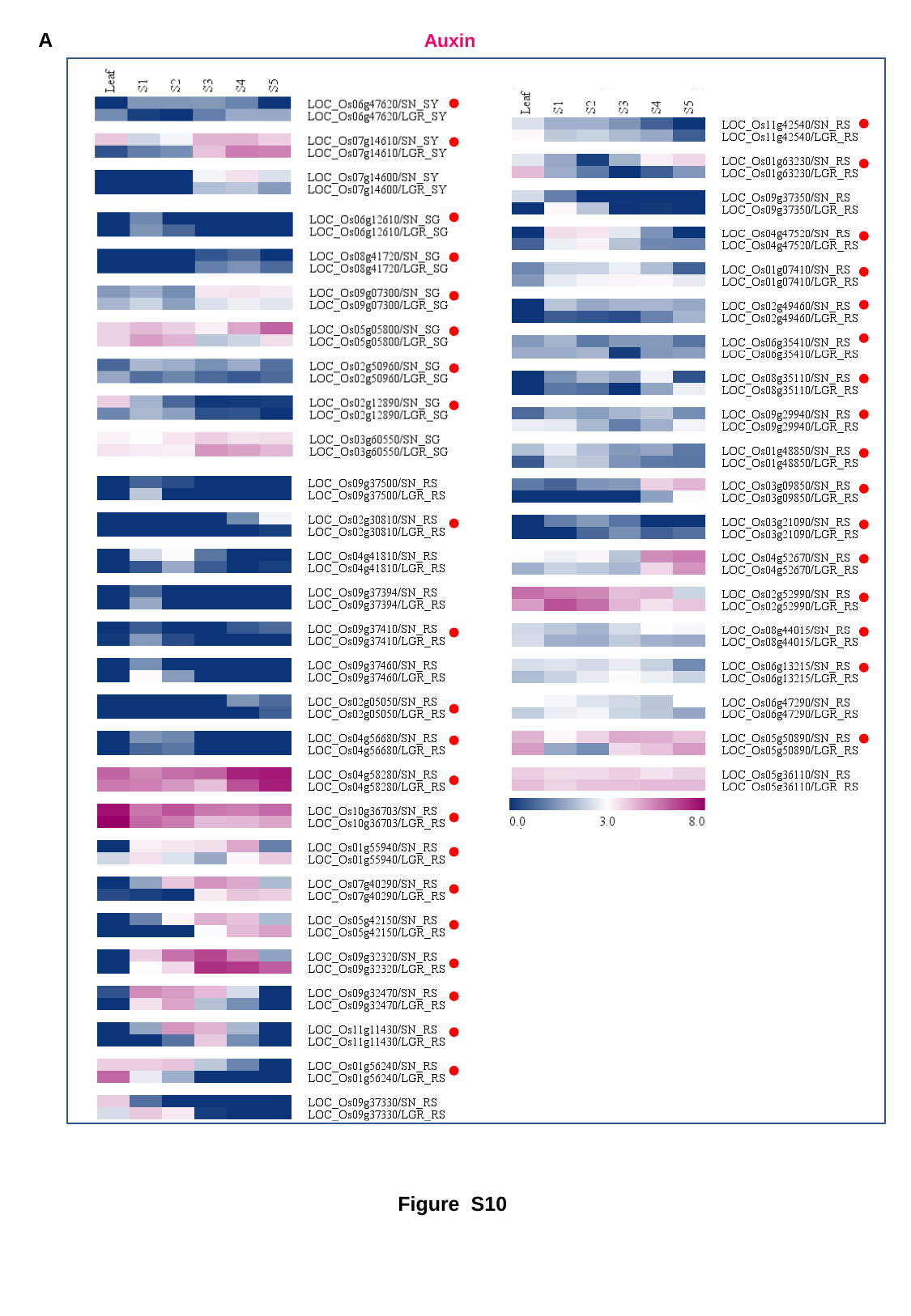

Auxin
A
cv
cv
cv
C
cv
cv
cv
cv
cv
cv
cv
cv
cv
cv
cv
cv
cv
cv
Figure S10

## Slide 14
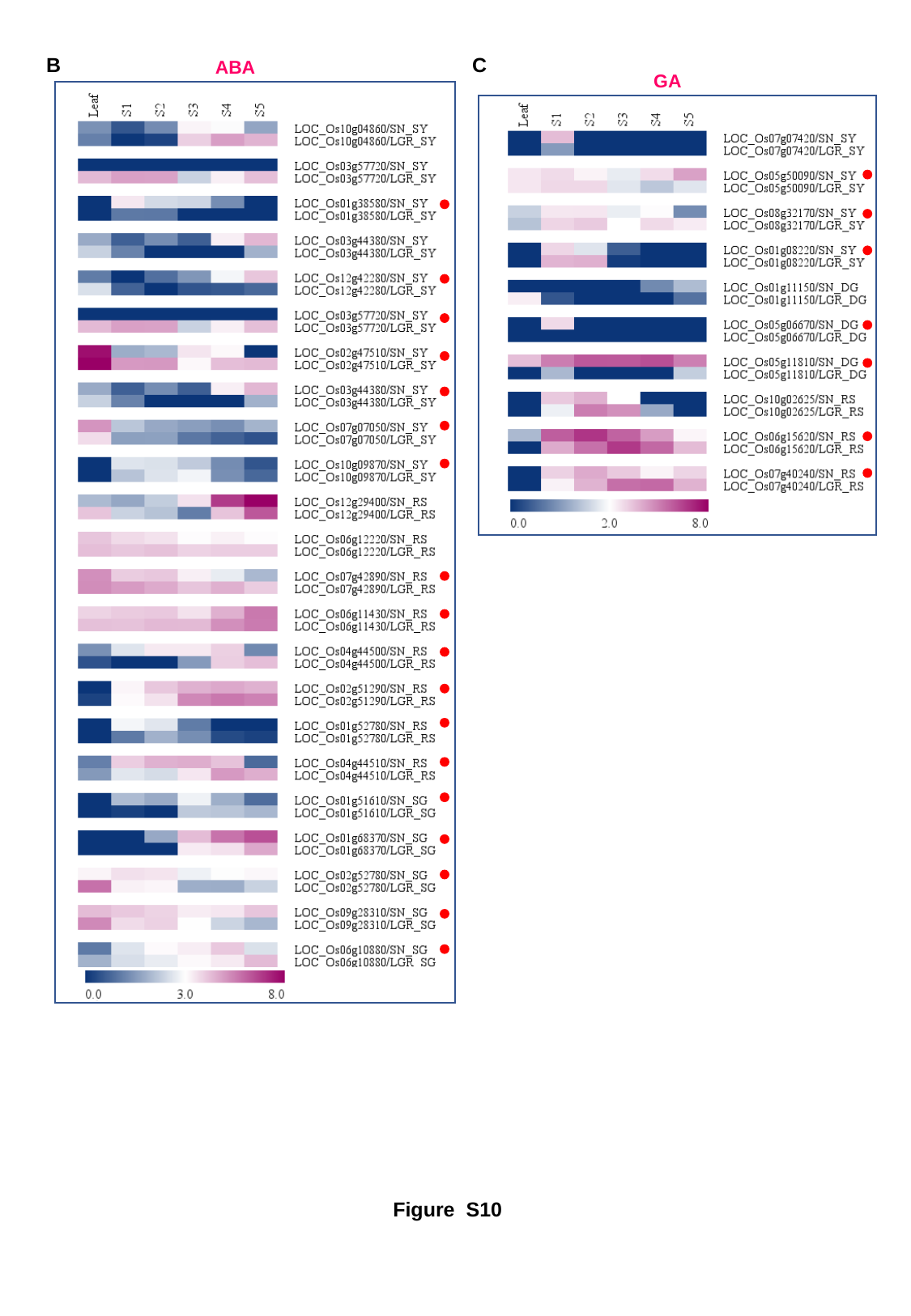

ABA
B
C
GA
Figure S10

## Slide 15
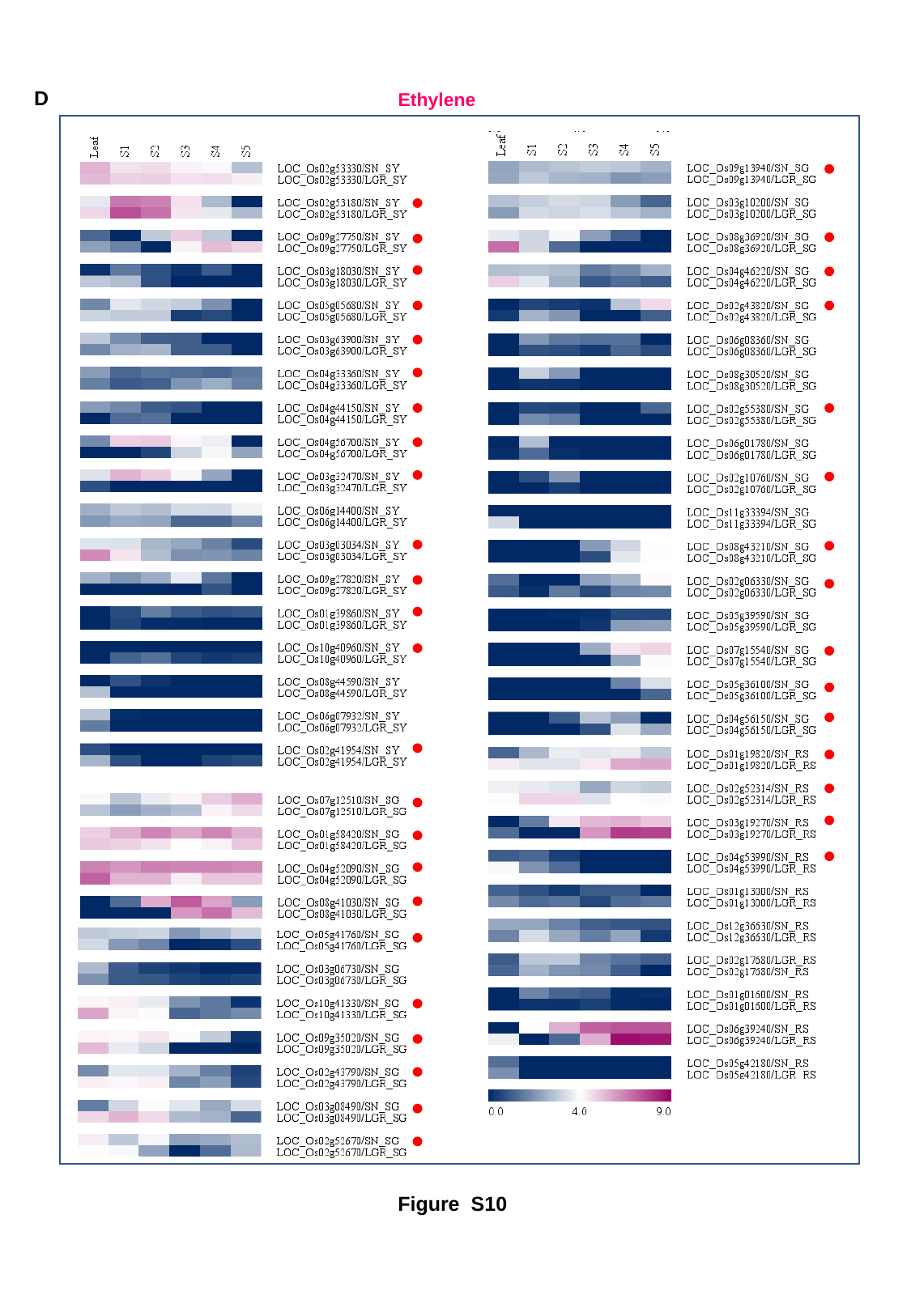

Ethylene
D
cv
cv
cv
cv
Figure S10

## Slide 16
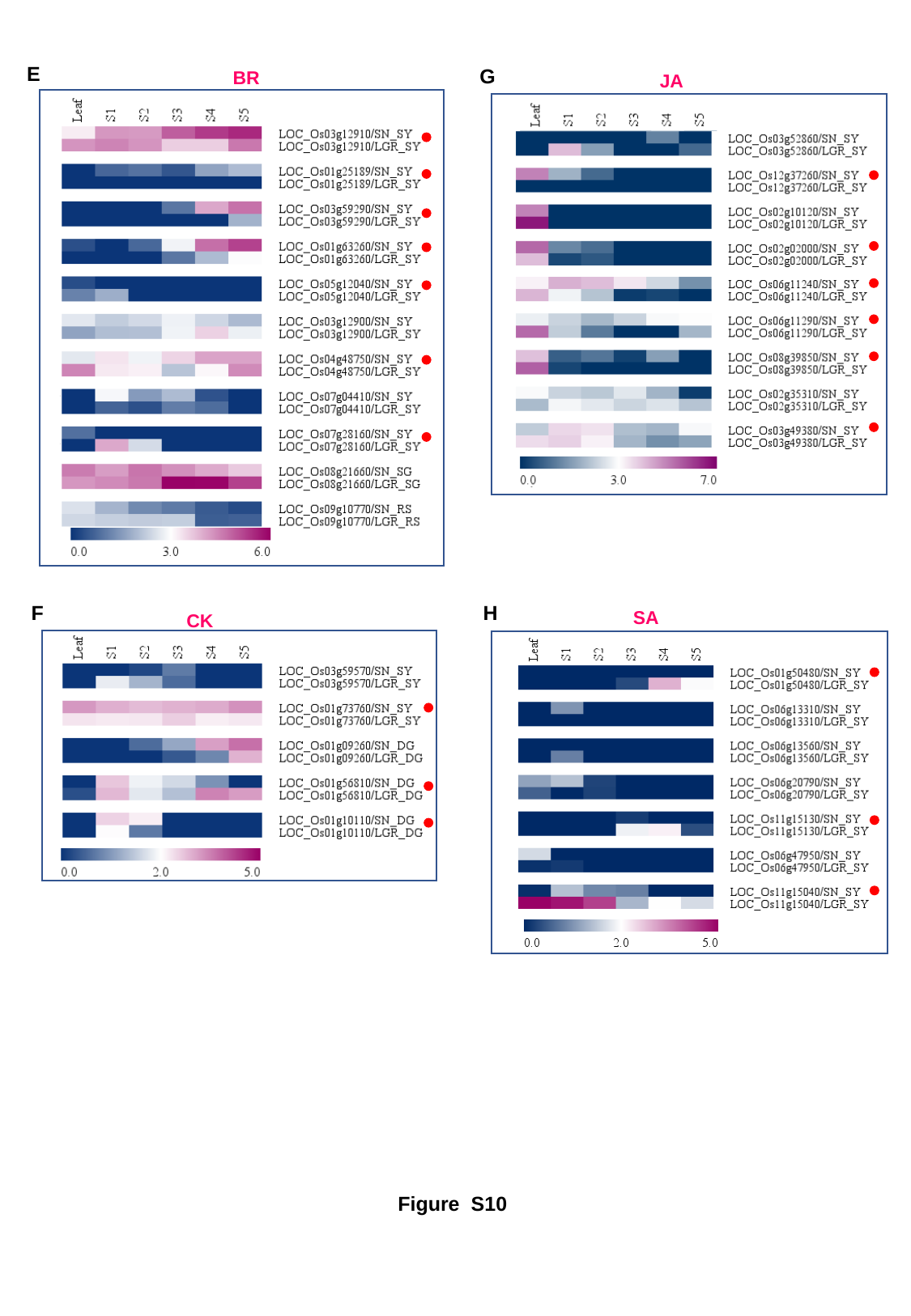

E
BR
G
JA
cv
F
H
CK
SA
Figure S10

## Slide 17
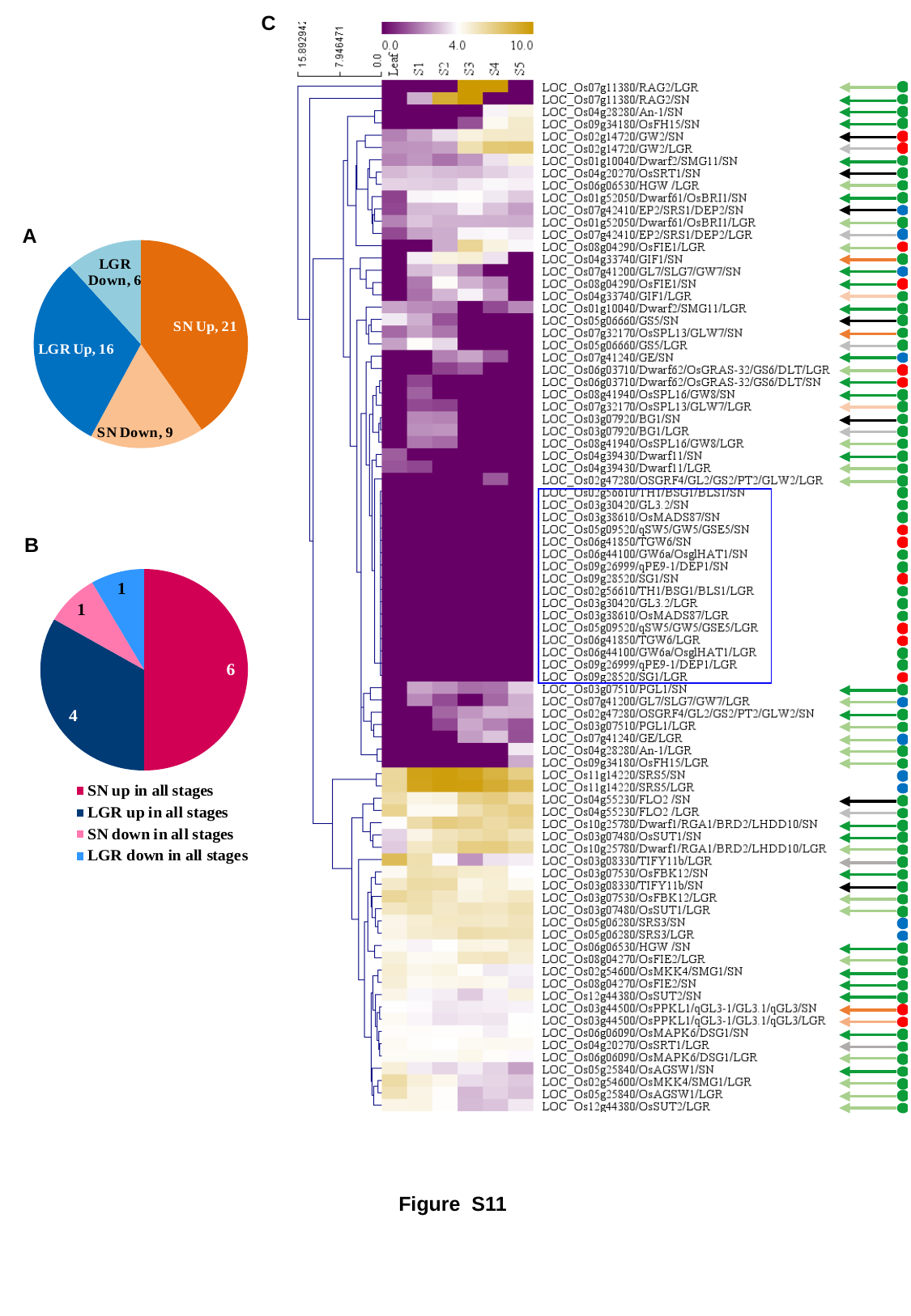

C
A
B
Figure S11

## Slide 18
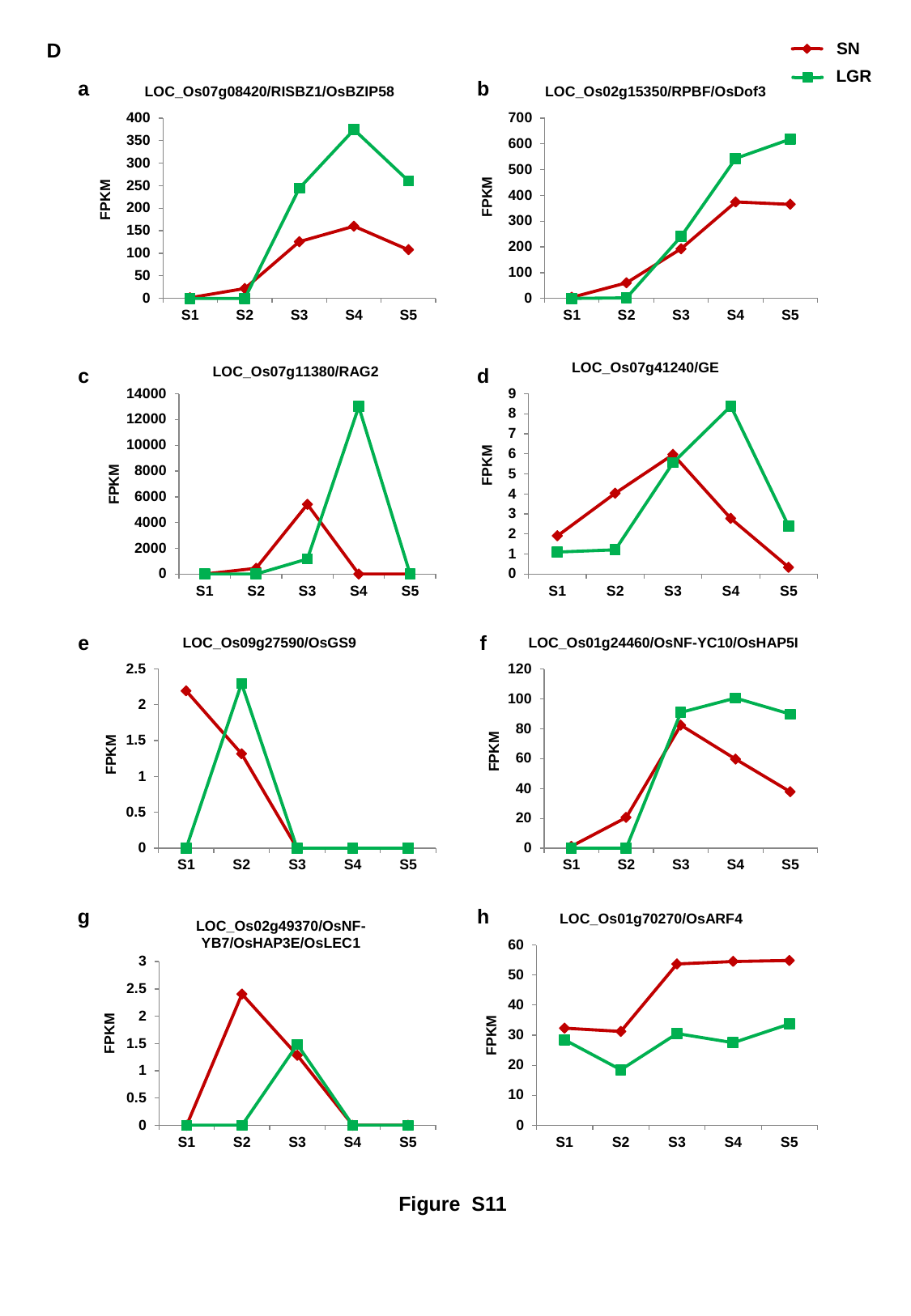

D
SN
LGR
a
b
c
d
e
f
g
h
Figure S11

## Slide 19
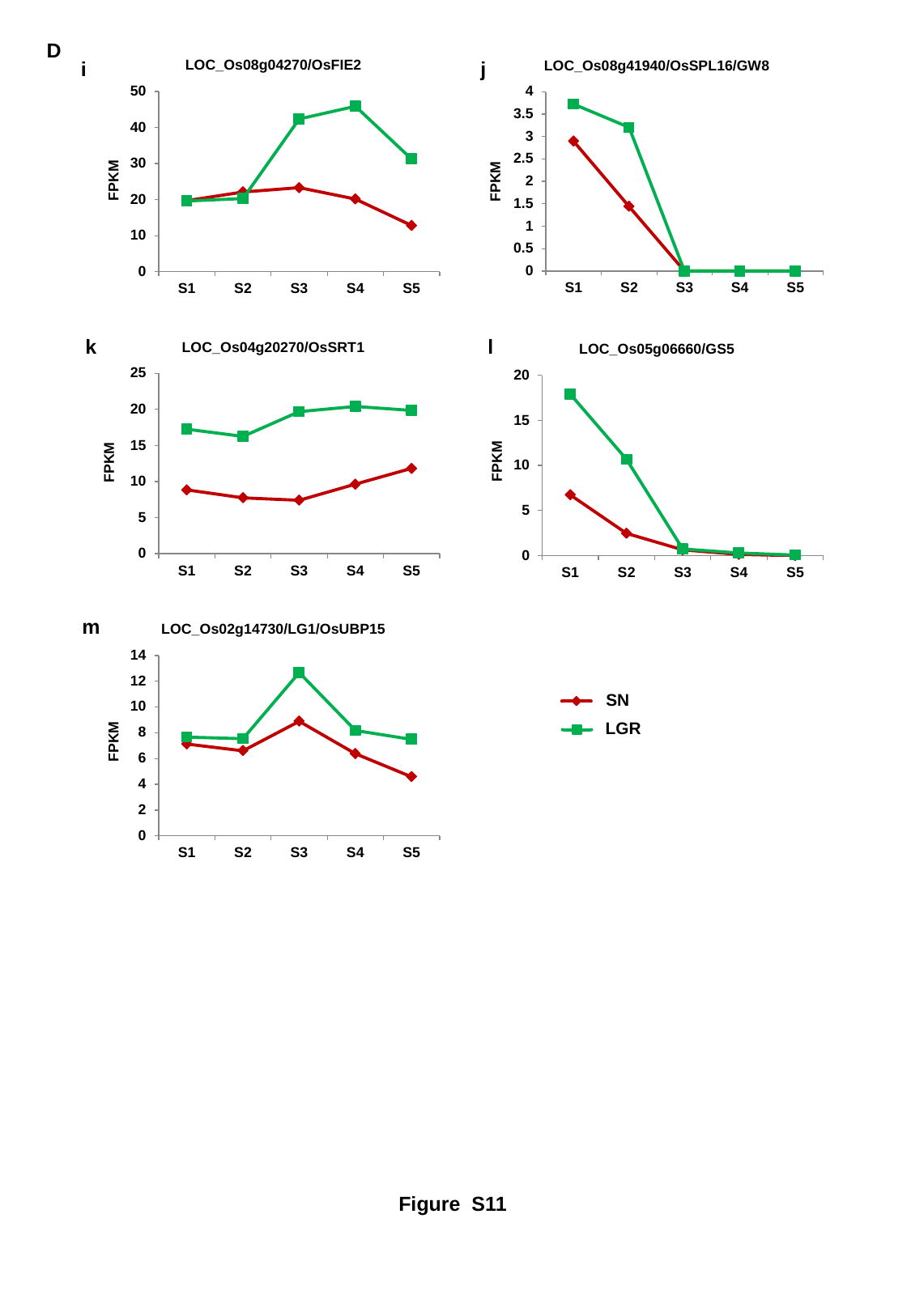

D
i
j
k
l
m
SN
LGR
Figure S11

## Slide 20
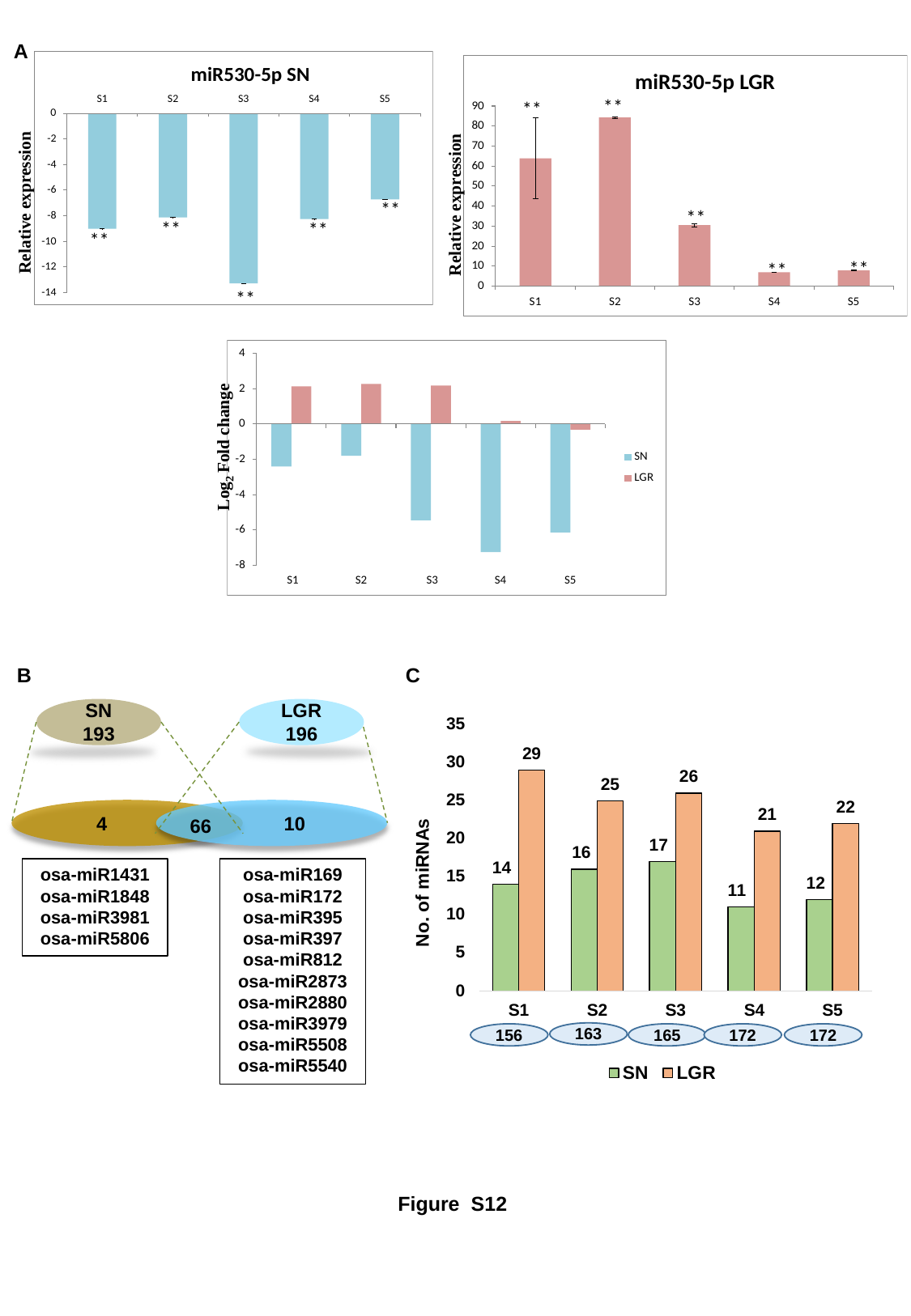

A
B
C
Figure S12

## Slide 21
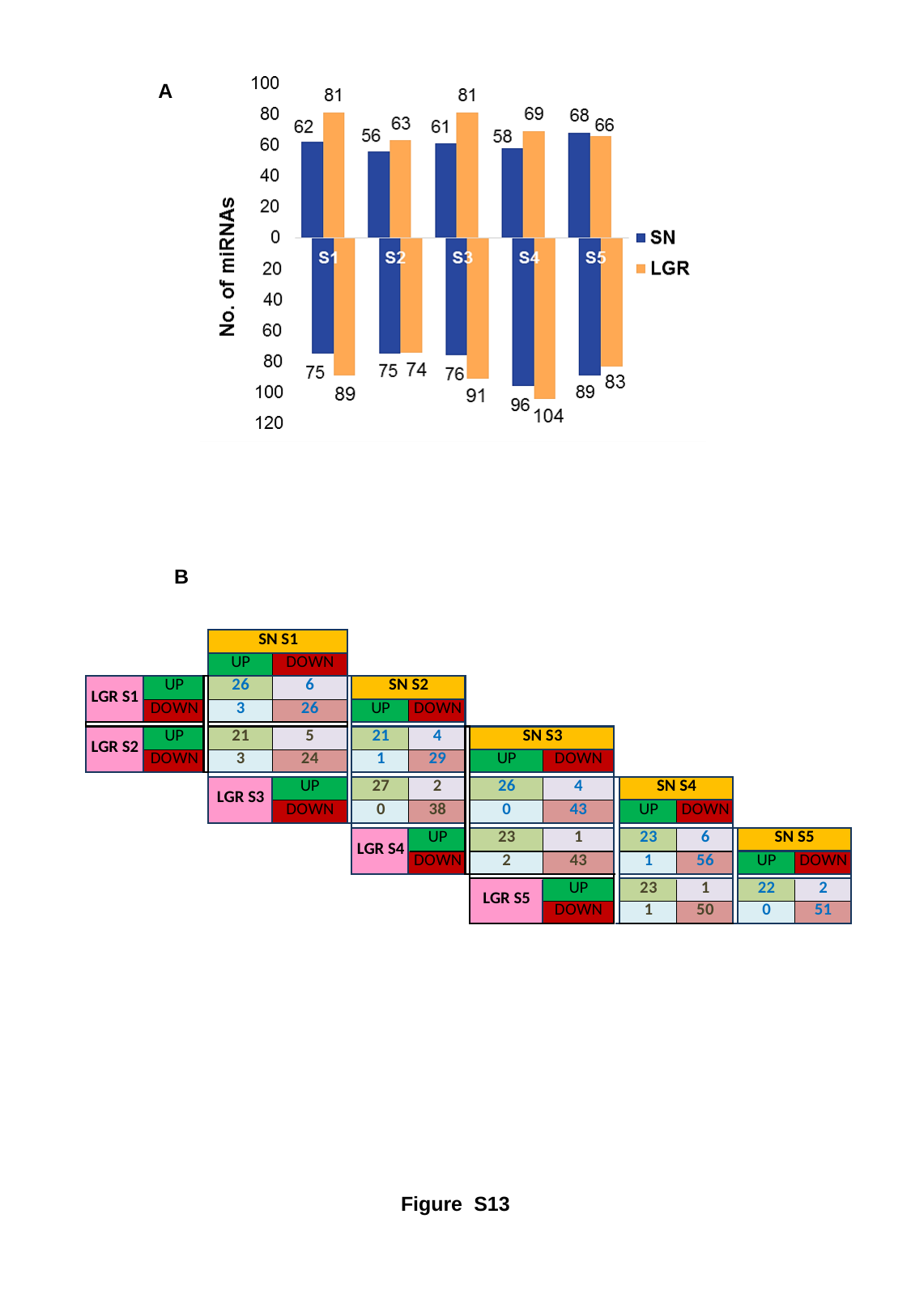

A
B
 Figure S13

## Slide 22
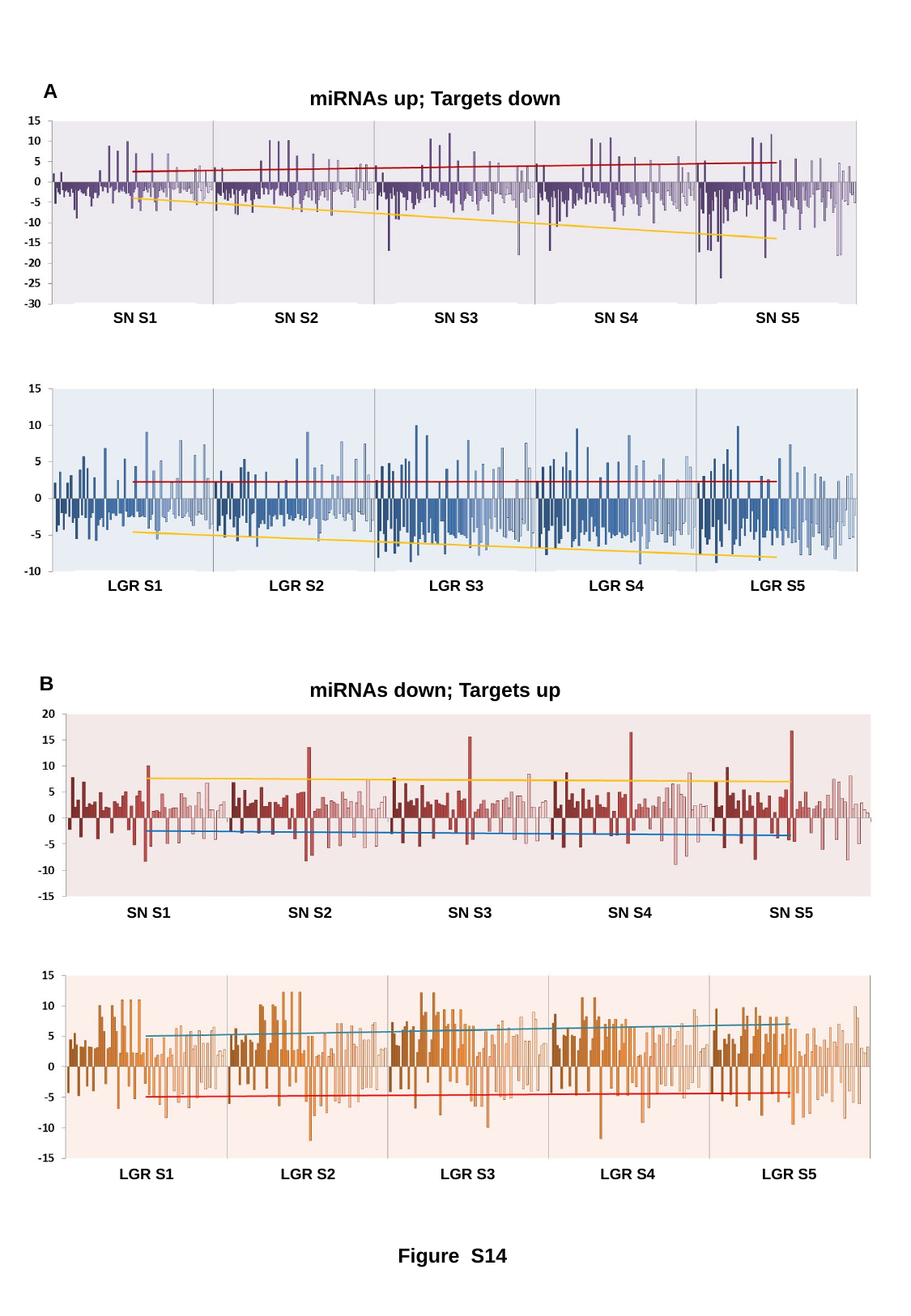

A
miRNAs up; Targets down
SN S1
SN S2
SN S3
SN S4
SN S5
LGR S1
LGR S2
LGR S3
LGR S4
LGR S5
B
miRNAs down; Targets up
SN S1
SN S2
SN S3
SN S4
SN S5
LGR S1
LGR S2
LGR S3
LGR S4
LGR S5
Figure S14

## Slide 23
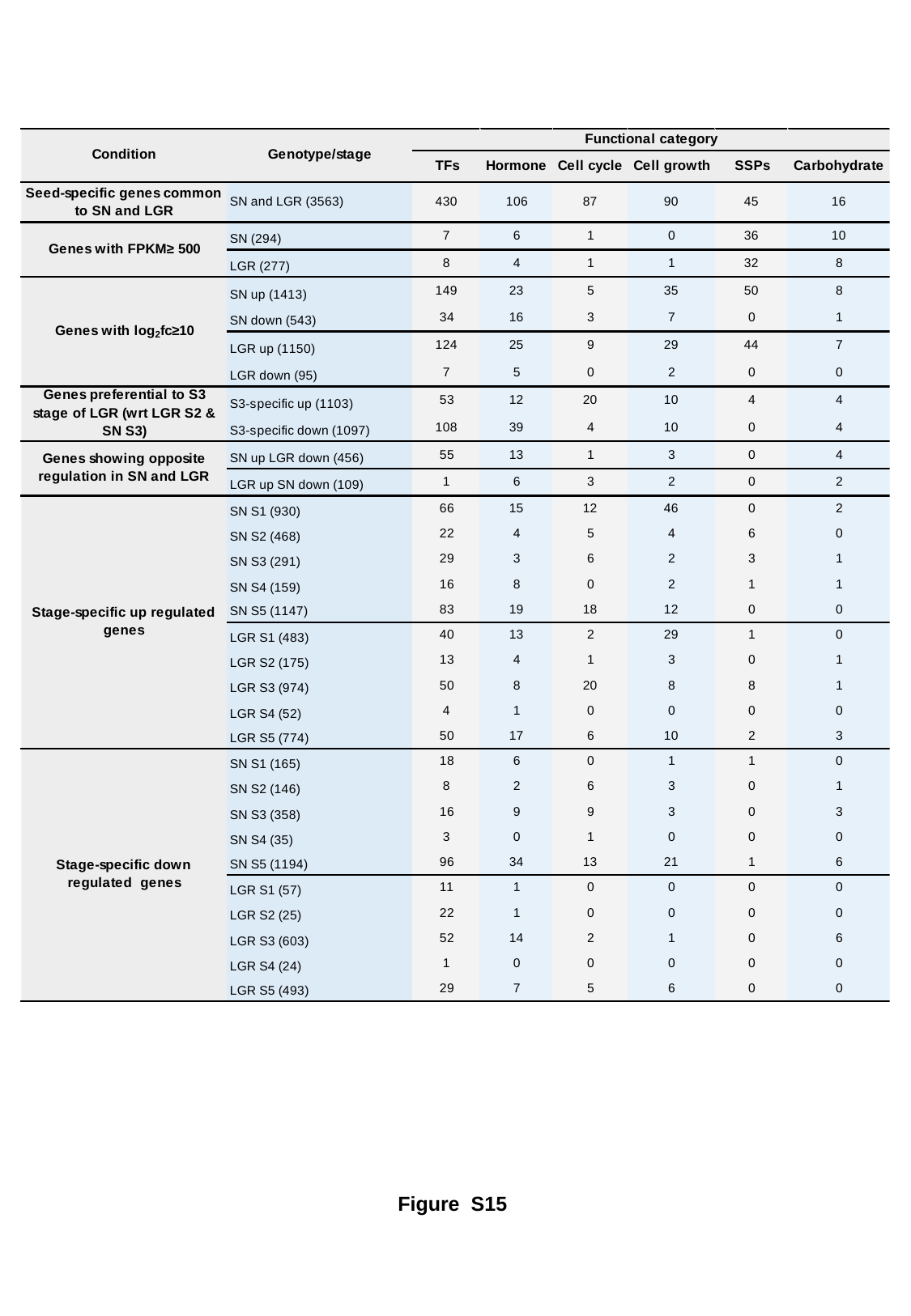

Figure S15

## Slide 24
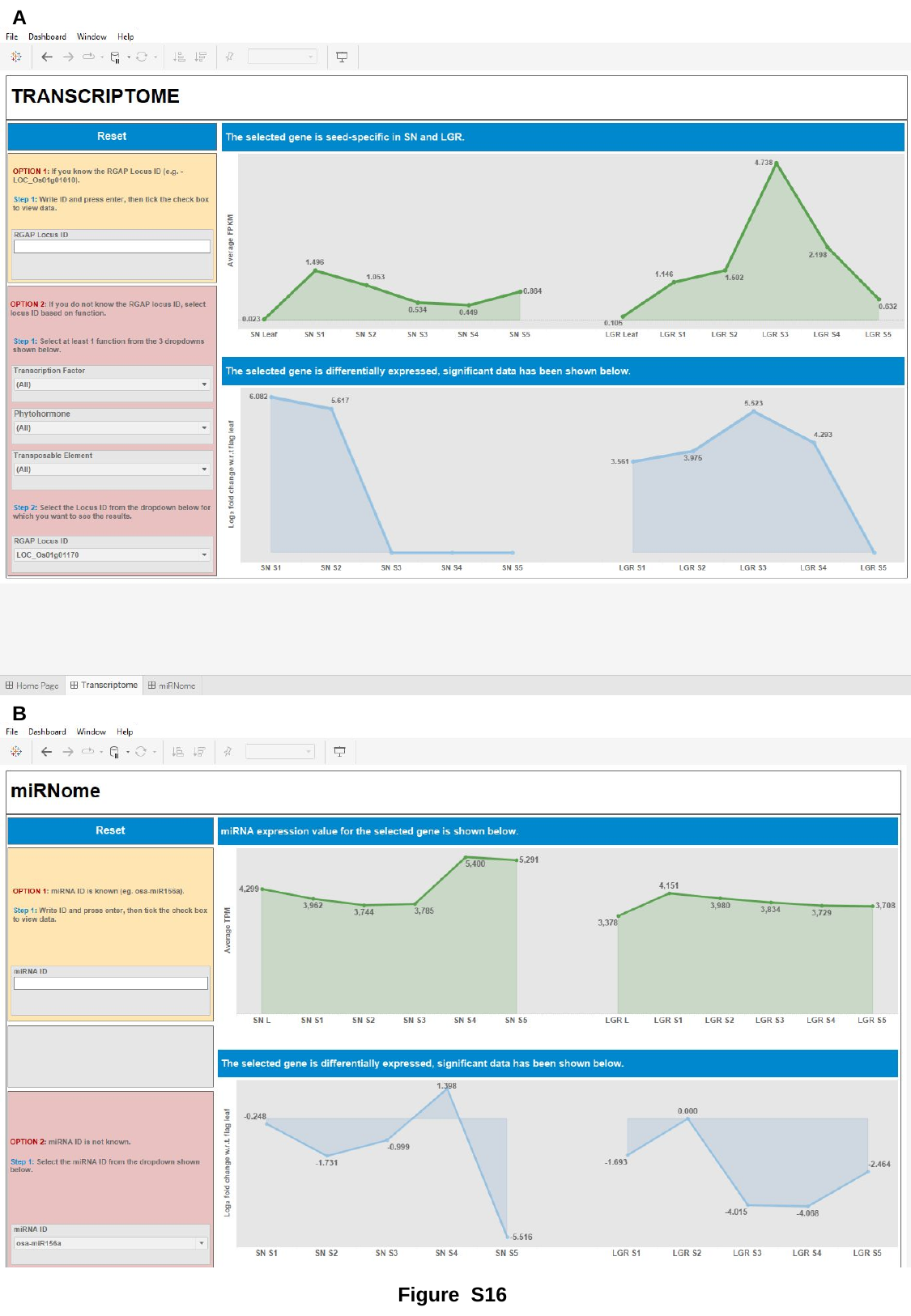

A
B
Figure S16
